# Catalytical nano-immunocomplexes for remote-controlled sono-metabolic checkpoint trimodal cancer therapy

**DOI:** 10.1038/s41467-022-31044-6

**Published:** 2022-06-16

**Authors:** Chi Zhang, Jingsheng Huang, Ziling Zeng, Shasha He, Penghui Cheng, Jingchao Li, Kanyi Pu

**Affiliations:** 1 School of Chemical and Biomedical Engineering, Nanyang Technological University, 70 Nanyang Drive, 637457 Singapore, Singapore; 2 School of Physical and Mathematical Sciences, Nanyang Technological University, 21 Nanyang Link, 637371 Singapore, Singapore; 3 Lee Kong Chian School of Medicine, Nanyang Technological University, 59 Nanyang Drive, 636921 Singapore, Singapore

**Keywords:** Nanoparticles, Nanotechnology in cancer, Cancer immunotherapy, Tumour immunology

## Abstract

Checkpoint immunotherapies have been combined with other therapeutic modalities to increase patient response rate and improve therapeutic outcome, which however exacerbates immune-related adverse events and requires to be carefully implemented in a narrowed therapeutic window. Strategies for precisely controlled combinational cancer immunotherapy can tackle this issue but remain lacking. We herein report a catalytical nano-immunocomplex for precise and persistent sono-metabolic checkpoint trimodal cancer therapy, whose full activities are only triggered by sono-irradiation in tumor microenvironment (TME). This nano-immunocomplex comprises three FDA-approved components, wherein checkpoint blockade inhibitor (anti-programmed death-ligand 1 antibody), immunometabolic reprogramming enzyme (adenosine deaminase, ADA), and sonosensitizer (hematoporphyrin) are covalently immobilized into one entity via acid-cleavable and singlet oxygen-activatable linkers. Thus, the activities of the nano-immunocomplex are initially silenced, and only under sono-irradiation in the acidic TME, the sonodynamic, checkpoint blockade, and immunometabolic reprogramming activities are remotely awakened. Due to the enzymatic conversion of adenosine to inosine by ADA, the nano-immunocomplex can reduce levels of intratumoral adenosine and inhibit A2A/A2B adenosine receptors-adenosinergic signaling, leading to efficient activation of immune effector cells and inhibition of immune suppressor cells in vivo. Thus, this study presents a generic and translatable nanoplatform towards precision combinational cancer immunotherapy.

## Abstract (web summary)

Ultrasound-based therapies in combination with immune checkpoint blockade have been shown to improve the efficacy of cancer immunotherapy. Here the authors report the design of a pH-responsive and sono-irradiation activatable nanosystem functionalized with anti-PD-L1 and adenosine deaminase for sono-metabolic cancer immunotherapy.

## Introduction

Cancer checkpoint immunotherapies that block negative immune regulatory pathways have recently been approved as first- or second-line therapies for a growing list of malignancies (e.g., melanoma, lymphoma, lung cancer, and bladder cancer)^1,2^. However, the patient response rate to checkpoint immunotherapies is generally low (10–30%) which is mainly due to the low tumor immunogenicity and the existence of immunosuppressive tumor microenvironment (TME)^3,4^. To potentiate the tumor for checkpoint immunotherapy, chemotherapy has been applied to induce immunogenic cell death (ICD) to enhance tumor immunogenicity^1^; whereas, targeted therapy that modulates immune activation signaling (e.g., toll-like receptor^5^ and stimulator of interferon gene signaling^6^) or immunosuppressive pathways (e.g., indoleamine 2,3-dioxygenase (IDO)^7^, macrophage-colony-stimulating factor (M-CSF) regulatory pathway^8^, etc.) has been used to reprogram immunosuppressive TME^9^. However, clinical data has revealed that combinational immunotherapies (e.g., programmed death 1 (PD-1) antibodies combined with chemotherapeutic paclitaxel or IDO inhibitors) generally exacerbate immune-related adverse events (irAEs) in patients^10^. This is attributed to the fact that chemotherapy and targeted therapy bring in toxicity to normal cells in addition to excessive activation of self-reactive T cells induced by immune checkpoint inhibitors^11^. Thus, despite their promise for enhanced antitumor efficacy, combinational cancer immunotherapies require to be carefully implemented in a narrow therapeutic window. Strategies for precisely controlled combinational cancer immunotherapy can potentially tackle this issue but remain lacking. Electromagnetic energy including magnetic fields^12^, light^13^, X-rays^14^, and ultrasound (US)^15^ provides a noninvasive and precise way to ablate tumors via localized irradiation, which minimizes the off-target side effects compared to chemotherapy and targeted therapy^16^. Electromagnetic therapies (such as phototherapy, magnetic hyperthermia therapy, and radiotherapy) have been proved to induce ICD for enhanced tumor immunogenicity^17^. Moreover, electromagnetic energy can serve as an exogenous stimulus to activate the pharmaceutical action of therapeutic agents. In comparison to the limited tissue-penetrating light, highly destructive X-ray, and complicatedly manipulated magnet field, US possesses properties of great tissue penetration depth (>10 cm), high spatiotemporal resolution, good controllability, and high safety^18^. By virtue of its clinic translation potential, sonotherapy has also been combined with immune checkpoint blockade (ICB) to improve the treatment efficiency for cancer immunotherapy^19,20^. However, the current sono-activatable strategy generally relies on US-triggered bursts of drug-loaded micro- or nano-bubbles^21,22^, which inevitably causes drug leakage in normal tissues due to the lack of covalent chemical bonding. Consequently, developing a cancer-specific sono-activatable strategy to precisely control drug activity and minimize off-target toxicity is highly desired.

**Fig. 1:**
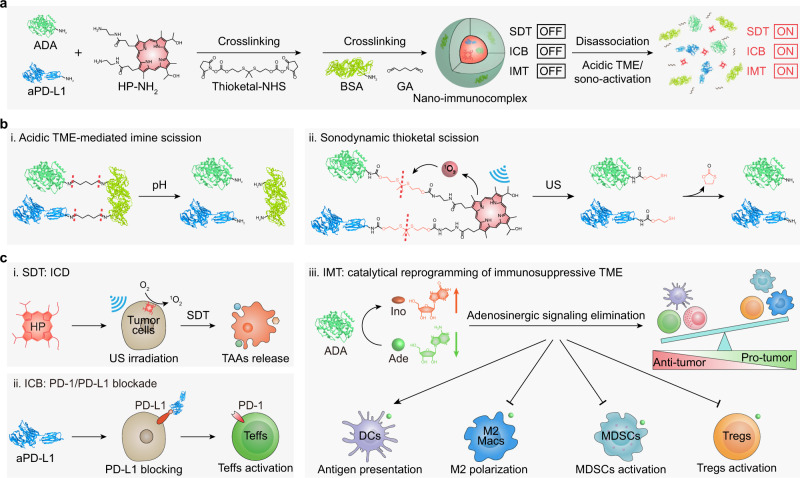
Schematic illustration of the smart catalytical nano-immunocomplex for remote-controlled sono-metabolic checkpoint trimodal cancer therapy. **a** Synthesis and activation of the nano-immunocomplex for synergistic sonodynamic therapy (SDT), immune checkpoint blockade (ICB), and immunometabolic therapy (IMT). **b** Acidic TME/sono-activatable molecular scission mechanisms of the nano-immunocomplex. (i) Acidic TME-mediated scission of imine bond. (ii) Sonodynamic scission of thioketal bond. **c** Acidic TME/sono-activatable cancer therapeutic mechanisms of the nano-immunocomplex. (i) Hematoporphyrin (HP)-mediated SDT to induce ^1^O_2_ generation, immunogenic cell death (ICD), and tumor-associated antigens (TAAs) release. (ii) aPD-L1-mediated ICB for effector T cells (Teffs) activation via blocking PD-1/PD-L1 signaling pathway. (iii) Adenosine deaminase (ADA)-mediated IMT to promote the antigen presentation of dendritic cells (DCs), reduce the protumorigenic polarization of M2 macrophages (M2 Macs), and inhibit the activation of myeloid-derived suppressor cells (MDSCs) and regulatory T cells (Tregs) via the elimination of adenosinergic pathway by catalytical conversion of adenosine (Ade) to inosine (Ino) for immunosuppressive TME reprogramming.

We herein report the development of a catalytical nano-immunocomplex for remote-controlled sono-metabolic checkpoint trimodal cancer therapy (Fig. 1). The nano-immunocomplex is composed of all Food and Drug Administration (FDA)-approved components including hematoporphyrin (HP), anti-programmed death-ligand 1 (PD-L1) antibody (aPD-L1), adenosine deaminase (ADA), and bovine serum albumin (BSA), which are assembled and covalently immobilized into one nanoparticle entity via acidic TME-cleavable imine and sono-activatable thioketal bonds. In physiological conditions, HP, aPD-L1, and ADA are all inert due to the covalent crosslinked immobilization of the nano-immunocomplex. Only in the concurrence of the acidic TME and sono-irradiation, the nano-immunocomplex not only generates singlet oxygen (^1^O_2_) to eliminate tumor cells and induce ICD for improved tumor immunogenicity but also unleashes aPD-L1 and ADA via the scission of imine and thioketal bonds. In this regard, aPD-L1 specifically binds to PD-L1 on the surface of tumor cells to block the PD-1/PD-L1 checkpoint pathway^23^, which can reinvigorate effector T cells (Teffs) activity and inhibit regulatory T cells (Tregs) function. ADA, a purine metabolic enzyme, can irreversibly deaminate adenosine (Ade) and persistently convert it to inosine (Ino) via the substitution of the amino group by a ketal group, leading to the catalytical depletion of the toxic metabolites Ade for immunometabolic therapy (IMT)^24^. This further results in the elimination of adenosinergic signaling (especially A2AR and A2BR signalling) and reprogramming of immunosuppressive TME, which finally promotes the activation of antitumorigenic immune effector cells (including dendritic cells (DCs) and effector T cells (Teffs)) and inhibition of protumorigenic immune suppressor cells (including myeloid-derived suppressor cells (MDSCs), M2-like macrophages (M2 Macs), and Tregs)^25–28^. As a result, the catalytical nano-immunocomplex exerts the synergistic antitumor effects via the cancer-specific and remote-controlled sono-metabolic checkpoint trimodal cancer therapy.

## Results

### Synthesis and characterization

The nano-immunocomplex was synthesized by the crosslinking of different molecules including ethylenediamine-modified HP (HP-NH_2_), aPD-L1, ADA, and BSA via the acidic TME-responsive crosslinker glutaraldehyde (GA) and sono-activatable bis-*N*-hydroxy succinimide (NHS)-conjugated thioketal (thioketal-NHS) crosslinker (Fig. 1a, Supplementary Fig. 1). First, thioketal-NHS and HP-NH_2_ were prepared, and their intermediates were characterized by ^1^H NMR and ESI-MS. Then, a mixture of HP-NH_2_, aPD-L1, and ADA in a 2 M sodium chloride solution was added with thioketal-NHS and stirred at 4 °C overnight. BSA and GA were further added into the solution and stirred at 4 °C for another 12 h. Thereafter, the obtained nano-immunocomplex (HP-proteins-crosslinking nanoparticles, abbreviated as HPNPs) was purified to remove the free crosslinkers and proteins and washed three times with phosphate buffer saline (PBS) solutions via ultrafiltration. For comparison, two control nanoparticles, HP-crosslinking nanoparticles (HNPs) and proteins-crosslinking nanoparticles (PNPs), were synthesized via similar methods. HNPs without the proteins (aPD-L1 and ADA) were fabricated by the crosslinking of HP-NH_2_, thioketal-NHS, BSA, and GA, while PNPs without the sonosensitizer (HP) were prepared by the crosslinking of aPD-L1, ADA, BSA, thioketal-NHS, and GA.

The physical and optical characteristics of these crosslinking NPs (HNP, PNP, and HPNP) were studied. The transmission electron microscopy (TEM) images indicated the similar particle sizes and uniform size distribution of HNP (~47 nm), PNP (~58 nm), and HPNP (~66 nm) (Supplementary Fig. 2a). The dynamic light scattering (DLS) analysis also verified the similar hydrodynamic sizes of HNP, PNP, and HPNP (Supplementary Fig. 2b) and high stability of HPNP in both PBS solutions and cell culture media (Supplementary Fig. 3). The zeta potential further confirmed their similar surface potential owing to the crosslinking of negatively charged BSA on the surface of these NPs (Supplementary Fig. 2c). HNP and HPNP had similar UV-vis absorption spectra with characteristic absorption peaks from the HP unit at 372 nm, 503 nm, 535 nm, 565 nm, and 620 nm (Supplementary Fig. 2d, e). The fluorescence spectra of HNP and HPNP were also similar with the characteristic emission peaks at 624 nm and 687 nm, which exhibited a slight redshift from the HP characteristic emission peaks at 613 nm and 668 nm owing to the existence of a π − π interaction of HP in the crosslinking nanoparticles^29^ (Supplementary Fig. 2f). In contrast, PNP did not show any characteristic absorption or emission peak because of the absence of HP. These data validated that these NPs had similar physical properties, and HNP and HPNP exhibited similar optical characteristics.

**Fig. 2:**
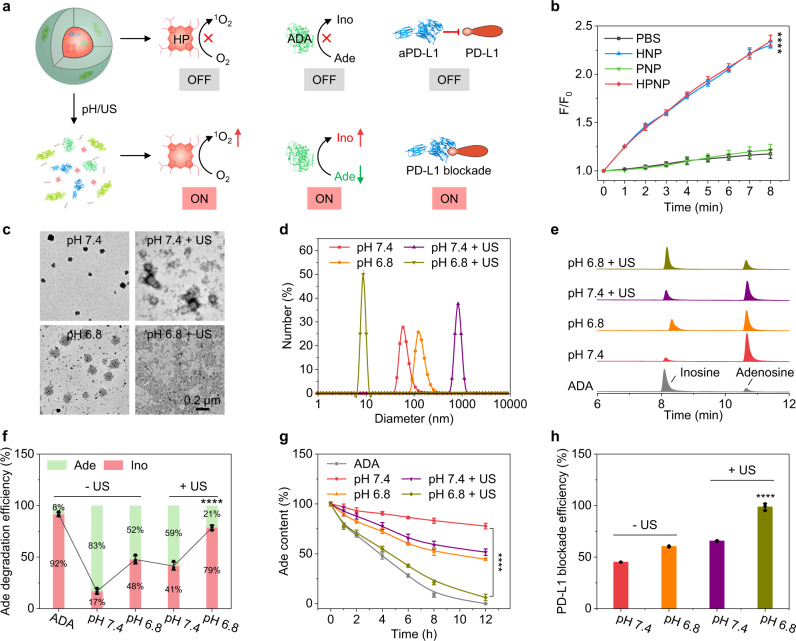
In vitro evaluation of the acidic TME/sono-activation of the nano-immunocomplex. **a** Schematic mechanisms of the acidic TME/sono-activated disassociation of the nano-immunocomplex and the OFF-ON switches of sonodynamic ^1^O_2_ generation, ADA-induced Ade degradation, and aPD-L1-mediated PD-L1 blockade. **b** The generation of ^1^O_2_ in HNP, PNP, and HPNP in 1× PBS buffer (pH 7.4) ([HP] = 20 μmol/L or [ADA] = 800 U/L) as a function of the sono-irradiation (1.0 MHz, 1.2 W/cm^2^, 50% duty cycle) time (*n* = 3). HPNP versus PBS: *p* < 0.0001. **c** TEM images and **d** DLS profiles of HPNPs in different conditions (pH 7.4, pH 7.4 with sono-irradiation, pH 6.8, and pH 6.8 with sono-irradiation). The experiments were repeated independently three times with similar results. **e** HPLC profiles and **f** quantification of Ade and Ino after 8 h incubation of HPNPs ([ADA] = 40 U/L) in PBS solutions containing Ade (20 mmol/L) with different treatments (pH 7.4, pH 7.4 with sono-irradiation, pH 6.8, and pH 6.8 with sono-irradiation) (*n* = 3). pH 6.8 with sono-irradiation versus pH 7.4: *p* < 0.0001. **g** Ade degradation profiles of HPNPs in the presence of Ade at different conditions (pH 7.4, pH 7.4 with sono-irradiation, pH 6.8, and pH 6.8 with sono-irradiation) (*n* = 3). pH 6.8 with sono-irradiation versus pH 7.4: *p* < 0.0001. **h** PD-L1 blockade efficiency after 12 h incubation of HPNPs at different conditions (pH 7.4, pH 7.4 with sono-irradiation, pH 6.8, and pH 6.8 with sono-irradiation) (*n* = 3). pH 6.8 with sono-irradiatio*n* versus pH 7.4: *p* < 0.0001. Sono-irradiation: 1.0 MHz, 1.2 W/cm^2^, 50% duty cycle for 6 min. Statistical significance in **b** and **g** was calculated via a two-tailed Student’s *t*-test. Statistical significance in **f** and **h** was calculated via one-way ANOVA with a Tukey post-hoc test. ****p < 0.0001. The mean values and SD are presented. Source data are provided as a Source Data file.

The cancer-specific and remote-controlled activation of the nano-immunocomplex was studied (Fig. 2a). The generation of ^1^O_2_ of these NPs was first detected by using singlet oxygen sensor green (SOSG) as a fluorescence indicator^30^. The frequency (1.0 MHz), power (1.2 W/cm^2^), duty cycle (50%), and irradiation time (≤8 min) of US were all controlled at a safety range to ensure the reversible permeabilization into the skin without safety concerns^31^. The fluorescence intensity of SOSG at 520 nm gradually increased for HNP and HPNP under sono-irradiation (1.0 MHz, 1.2 W/cm^2^, 50% duty cycle), which demonstrated the generation of ^1^O_2_ (Fig. 2b). The fluorescence intensity of HNP and HPNP exhibited about 2.3-fold increment after sono-irradiation for 8 min. In contrast, the fluorescence intensity of the control and PNP groups did not show an obvious increment. These data validated that the generation of ^1^O_2_ for HNP and HPNP upon sono-irradiation was owing to the existence of HP and the crosslinking of different proteins (aPD-L1 and ADA) did not affect the sonodynamic properties of HP.

Afterward, the morphological changes and functional activation of the smart nano-immunocomplex were studied. The TEM images of HPNP exhibited a spherical morphology and uniform size distribution in pH 7.4 solutions (Fig. 2c). After sono-irradiation or incubation in pH 6.8 solutions, the particle sizes were enlarged and a small portion of HPNP was disassociated owing to the scission of the acidic TME-responsive imine bonds or the sono-activatable thioketal bonds. Notably, the spheric nanoparticles almost disappeared after the simultaneous treatments of sono-irradiation and incubation in pH 6.8 solutions, indicating the complete disassociation of HPNP. The DLS analysis further elucidated the changes of particle sizes after different treatments, which was consistent with the TEM results (Fig. 2d). The generation of ^1^O_2_ in acid conditions exhibited an obvious increase compared to that in pH 7.4 solutions (Supplementary Fig. 4), further indicating the acidic TME-responsive disassociation of HPNP.

To verify the acidic TME/sono-activation of ADA along with the disassociation of nanoparticles, high-performance liquid chromatography (HPLC) analysis was conducted after incubation of HPNP in PBS solutions containing the substrate Ade (Fig. 2e). The elution peaks at 8.1 and 10.6 min were ascribed to Ino and Ade, respectively. The free enzymes ADA exhibited a high catalytic activity with only 8% residues of Ade in pH 7.4 solutions after 8 h incubation (Fig. 2f). Meanwhile, the metabolites Ino relatively increased due to the conversion of Ade to Ino via the catalysis of ADA. After incubation of HPNP in pH 6.8 solutions for 8 h and subsequent sono-irradiation for 6 min, the relative Ade contents greatly decreased to 21%. In contrast, the remaining Ade contents were 83%, 52%, or 59% after the incubation of HPNP in the conditions of pH 7.4, pH 6.8, or pH 7.4 with sono-irradiation. This was owing to the spatially restricted interaction of Ade with ADA and the crosslinking-mediated shielding of the catalytic sites of ADA in the nano-immunocomplex. The Ade degradation profiles further validated the acidic TME/sono-activation of ADA (Fig. 2g, Supplementary Fig. 5). To further study the acidic TME/sono-activation of aPD-L1, the PD-L1 blockade efficiency was detected by enzyme-linked immunosorbent assay (ELISA). The ELISA results exhibited the highest PD-L1 blockade efficiency (99%) after incubation of HPNP in pH 6.8 solutions and sono-irradiation (Fig. 2h). In contrast, the PD-L1 blockade efficiency was only 45%, 60%, or 66% after incubation of HPNP in the conditions of pH 7.4, pH 6.8, or pH 7.4 with sono-irradiation. This is due to the incomplete disassociation of HPNP and partial release of aPD-L1 in the conditions of pH 7.4, pH 6.8, or pH 7.4 with sono-irradiation. These results further validated the acidic TME/sono-activation of the nano-immunocomplex for ADA-induced Ade degradation and aPD-L1-mediated PD-L1 blockade.

### In vitro studies of sono-metabolic checkpoint trimodal cancer therapy

**Fig. 3:**
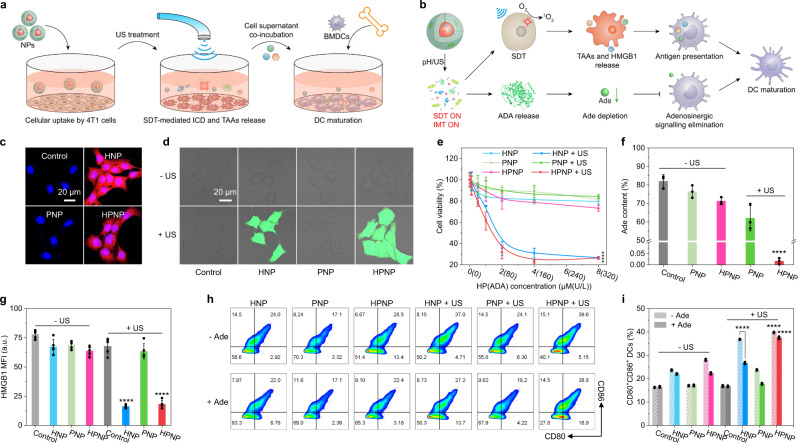
In vitro nano-immunocomplex-mediated activatable sono-metabolic checkpoint trimodal cancer therapy. **a** Schematic illustration of experiment implementation for cellular uptake, SDT, ICD induction, and BMDC maturation. **b** Proposed mechanism of acidic TME/sono-activation of SDT and IMT for antigen presentation and DC maturation. **c** Confocal fluorescence images of 4T1 cancer cells after 12 h incubation with HNP, PNP, or HPNP ([HP] = 20 μmol/L or [ADA] = 800 U/L). **d** Confocal fluorescence images of 4T1 cells after 12 h incubation with HNP, PNP, or HPNP ([HP] = 20 μmol/L or [ADA] = 800 U/L), followed by staining with H_2_DCFDA with or without sono-irradiation (1.0 MHz, 1.2 W/cm^2^, 50% duty cycle) for 6 min. The experiments in **c** and **d** were repeated independently three times with similar results. **e** Relative cell viabilities of 4T1 cells after 24 h incubation with HNP, PNP, or HPNP at different HP or ADA concentrations with or without sono-irradiation for 6 min (*n* = 3). HPNP + US versus HPNP: *p* < 0.0001. **f** Relative Ade content in the cell culture medium after 12 h incubation of 4T1 cells with HNP, PNP, or HPNP ([HP] = 1 μmol/L or [ADA] = 40 U/L) in the presence of additional Ade (10 mmol/L) by HPLC assay (*n* = 3). HPNP + US versus HPNP: *p* < 0.0001. **g** Quantification of HMGB1 expression in 4T1 cell nuclei after 12 h incubation with HNP, PNP, or HPNP ([HP] = 20 μmol/L or [ADA] = 800 U/L) with or without sono-irradiation for 6 min (*n* = 5). HPNP + US versus HPNP: *p* < 0.0001; HNP + US versus HNP: *p* < 0.0001. **h** Flow cytometry assay and **i** quantification of the matured DCs (CD80^+^CD86^+^) after 12 h incubation of BMDCs with the 4T1 cell supernatants with different treatments (*n* = 3). 4T1 cells were incubated with HNP, PNP, or HPNP ([HP] = 20 μmol/L or [ADA] = 800 U/L) with or without Ade addition or sono-irradiation for 6 min. HNP + US with Ade addition versus without Ade addition: *p* < 0.0001; HPNP + US versus HPNP with Ade addition: *p* < 0.0001; HPNP + US versus HPNP without Ade addition: *p* < 0.0001. Statistical significance in **e** was calculated via two-tailed Student’s *t*-test. Statistical significance in **f**, **h**, and **i** was calculated via one-way ANOVA with a Tukey post-hoc test. ****p < 0.0001. The mean values and SD are presented. Source data are provided as a Source Data file.

To evaluate the cellular uptake of NPs, 4T1 murine breast cancer cells were first incubated with HNP, PNP, or HPNP for 12 h and imaged by confocal fluorescence microscopy (Fig. 3a, b). The red fluorescence signals could be detected in HNP- or HPNP-incubated cells (Fig. 3c). The mean fluorescence intensities (MFIs) of HNP- or HPNP-incubated cells were 79.8 or 69.4, indicating their similar cellular uptake by 4T1 cells (Supplementary Fig. 6a). In contrast, the PNP-incubated cells did not show obvious red fluorescence, because the red fluorescence signals came from the sonosensitizer HP. The intracellular lysosome colocalization analysis further indicated the effective endosomal escape of NPs after incubation for 6 h (Supplementary Fig. 7).

The sonodynamic activity and cytotoxicity of NPs were studied in 4T1 cells. The ^1^O_2_ fluorescent turn-on probe 2’,7’-dichlorodihydrofluorescein diacetate (H_2_DCFDA) was used to evaluate the generation of ^1^O_2_ in cells after sono-irradiation^30^. The obvious green fluorescence signals from the ^1^O_2_-oxidized 2’,7’-dichlorofluorescein (DCF) could only be detected in HNP- or HPNP-incubated and sono-irradiated cells (Fig. 3d). The MFIs of HNP- or HPNP-incubated and sono-irradiated cells were 92.7 or 93.5, both of which were about 25-fold higher than that of the unirradiated cells (Supplementary Fig. 6b). Afterward, the cytotoxicity of NPs was studied by using the 5-(3-carboxymethoxyphenyl)-2-(4,5-dimethylthiazolyl)-3-(4-sulfophenyl)-tetrazolium (MTS) assay. Before sono-irradiation, the HNP-, PNP-, or HPNP-incubated cells exhibited relatively low cytotoxicity with the cell viability of above 80% even at a high concentration of NPs (Fig. 3e). This demonstrated the low cytotoxicity of these NPs without sono-irradiation. After sono-irradiation, the HNP- or HPNP-incubated cells showed the increased cytotoxicity in a concentration-dependent manner. The cell viabilities of HNP- or HPNP-incubated and sono-irradiated cells decreased to ~25% at the HP concentration of 8 μmol/L. These data validated the good sonodynamic therapeutic properties of the nano-immunocomplex.

The acidic TME/sono-activated Ade degradation was further studied in 4T1 cells by using HPLC analysis of the Ade contents in the cell supernatant. The relative Ade content was 82.02% without any treatments owing to the autologous metabolism of Ade by 4T1 cells (Fig. 3f). After incubation with HPNP and subsequent sono-irradiation for 6 min, the relative Ade content exhibited a remarkable decrease to 0.02%. However, PNP-incubated cells, HPNP-incubated cells, or PNP-incubated and sono-irradiated cells did not show obvious decrease of the Ade contents (76.40%, 71.27%, or 62.02%). These data validated that sono-irradiation could remotely control the activation of ADA, leading to the degradation of Ade in cell levels after incubation with the nano-immunocomplex.

To investigate the antitumor immunity after the acidic TME/sono-activated degradation of Ade, ICD and DC maturation were further studied (Fig. 3a, b). The release of high-mobility group protein B1 (HMGB1) from the cell nuclei to extracellular supernatants was first detected to evaluate ICD^32^. The HNP- or HPNP-incubated and sono-irradiated 4T1 cells exhibited obvious decrease of green fluorescence signals from FITC-labeled anti-HMGB1 antibodies in the cell nuclei (Supplementary Fig. 8). The MFIs of HNP- or HPNP-incubated and sono-irradiated cells were 16.3 or 18.2 (Fig. 3g), which exhibited 4.1- or 3.5-fold decreases compared to the unirradiated cells. However, PNP-incubated cells with or without sono-irradiation did not exhibit obvious decrease of the green fluorescence signals from FITC-labeled anti-HMGB1 antibodies. This confirmed that ICD was induced by HP-mediated sonodynamic therapeutic activity. Afterward, DC maturation was detected via flow cytometry analysis. Bone marrow-derived dendritic cells (BMDCs) were first extracted from the mice and further cultured in the RPMI 1640 cell culture medium^33^. Then the purified BMDCs were incubated with the 4T1 cell supernatants after different treatments for 12 h. The proportion of matured DCs (CD80^+^CD86^+^) was 23.7%, 17.1%, or 28.1% after treatment with HNP-, PNP-, or HPNP-incubated cell supernatants without sono-irradiation (Fig. 3h, i). After sono-irradiation, HNP- or HPNP-incubated groups exhibited 1.6-fold or 1.4-fold increments relative to that without sono-irradiation. This was owing to the HP-mediated SDT and ICD for enhanced maturation of DCs. Ade acts as an immunosuppressive metabolite, which can impair the antigen presentation function of DCs with decreased expression of CD80 and CD86 via the adenosinergic A2BR signaling pathway^25,34,35^. Thus, DC maturation was further evaluated with the addition of Ade in the culture medium. The proportion of matured DCs greatly decreased by 1.4- and 1.3-fold in HNP- and PNP-incubated and sono-irradiated groups in the presence of additional Ade, respectively. However, the HPNP-incubated and sono-irradiated groups did not show obvious decrease of the matured DCs, which was ascribed to the acidic TME/sono-activated Ade degradation. As a result, these data demonstrated that the nano-immunocomplex could effectively induce ICD and DC maturation for enhanced antigen presentation.

### In vivo sono-metabolic checkpoint trimodal cancer therapy

**Fig. 4:**
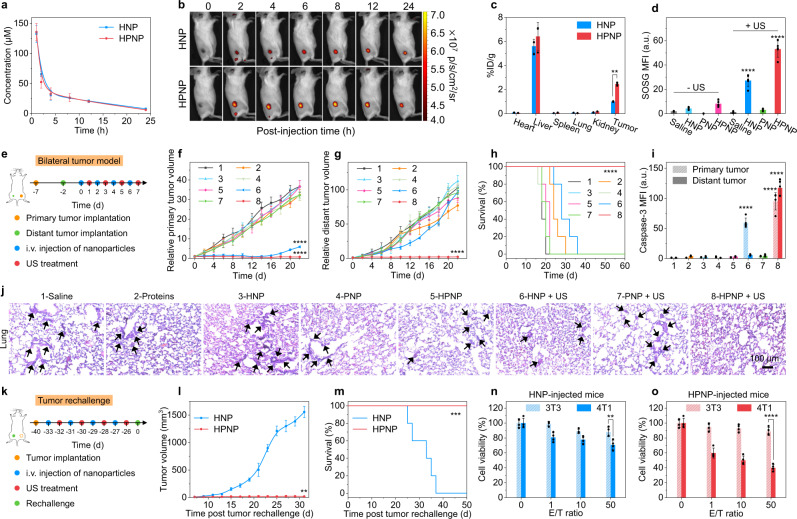
In vivo NIR fluorescence imaging and nano-immunocomplex-mediated activatable sono-metabolic checkpoint trimodal cancer therapy. **a** Pharmacokinetic analysis of blood concentration of HP in BALB/c mice at t = 1, 2, 4, 8, 12, or 24 h post-injection of HNP or HPNP (*n* = 3). **b** NIR fluorescence imaging of 4T1 tumor-bearing BALB/c mice at t = 0, 2, 4, 6, 8, 12, or 24 h post-injection of HNP or HPNP (injection dose: 200 μL, [HP] = 1 mmol/L, or [ADA] = 40 U/mL). **c** Biodistribution of HNP or HPNP in 4T1 tumor-bearing mice at 24 h after systemic administration (*n* = 3). HPNP versus HNP in tumors: *p* = 0.0035. **d** Quantitative analysis of SOSG MFIs in tumor tissues from HNP-, PNP-, or HPNP-injected mice with or without sono-irradiation (1.0 MHz, 1.2 W/cm^2^, 50% duty cycle) for 6 min (*n* = 5). HNP + US versus HNP: *p* < 0.0001; HPNP + US versus HPNP: *p* < 0.0001. **e** Schematic illustration of the schedule for bilateral tumor model implantation and nano-immunocomplex-mediated activatable sono-metabolic checkpoint trimodal cancer therapy. Growth curves of primary tumors (**f**) and distant tumors (**g**) after different treatments (*n* = 5). 6 versus 1 in **f**: *p* < 0.0001; 8 versus 1 in **f**: *p* < 0.0001; 8 versus 1 in **g**: *p* < 0.0001. **h** Survival curves for the mice after different treatments using the Kaplan–Meier method (*n* = 5). *p* < 0.0001. **i** Quantification of caspase-3 expression (*n* = 5). 6 versus 1 in primary tumors: *p* < 0.0001; 8 versus 1 in primary tumors: *p* < 0.0001; 8 versus 1 in distant tumors: *p* < 0.0001. **j** Histological H&E staining of lung in 4T1 tumor-bearing mice. Images are representative of three biologically independent mice. **k** Schematic illustration of the schedule for tumor rechallenge study. **l** Growth curves of the reinoculated tumors (*n* = 5). HPNP versus HNP: *p* = 0.0014. **m** Survival curves for the mice with reinoculated tumors using the Kaplan–Meier method (*n* = 5). *p* = 0.0003. Cell viability of 3T3 and 4T1 cells as target cells (T) after incubation with effector T cells (E) isolated from spleen of the rechallenged and HNP- (**n**) or HPNP-injected (**o**) mice as a function of the E/T ratios (*n* = 4). 4T1 versus 3T3 in **n**: *p* = 0.0027; 4T1 versus 3T3 in **o**: *p* < 0.0001. Statistical significance i*n* **c**, **d**, **i**, **n**, and **o** was calculated via one-way ANOVA with a Tukey post-hoc test. Statistical significance in **f**, **g**, and **l** was calculated via two-tailed Student’s *t*-test. Statistical significance in **h** and **m** was calculated via the log-rank test. **p < 0.01, ***p < 0.001, and ****p < 0.0001. The mean values and SD are presented. Source data are provided as a Source Data file.

Nano-immunocomplex-mediated activatable sono-metabolic checkpoint trimodal cancer therapy was studied in 4T1 tumor-bearing BALB/c mice. The pharmacokinetics of HNP and HPNP were first studied by detecting the fluorescence signals from HP via collecting the blood at different timepoints. HNP- or HPNP-injected mice exhibited similar pharmacokinetic profiles owing to the similar particle sizes and structures (Fig. 4a). To confirm the optimal timepoint for sono-irradiation, the accumulation of NPs in tumor tissues was then evaluated using in vivo NIR fluorescence imaging in 4T1 tumor-bearing mice after intravenous injection of HNP or HPNP. The fluorescence intensities at tumor sites of HNP- or HPNP-injected mice gradually increased and reached the maximum values at 12 h post-injection, which were 4.0- and 5.6-fold higher than that of the background signals, respectively (Fig. 4b, Supplementary Fig. 9a). Meanwhile, the ex vivo fluorescence imaging of major organs and tissue biodistribution analysis of NPs further demonstrated their effective tumor accumulation abilities (Fig. 4c, Supplementary Fig. 9b, c). Notably, the tumor accumulation effect of HPNP was stronger than HNP, which was owing to the enhanced tumor-targeting ability of the activated aPD-L1. The immunofluorescence staining images of the tumor tissues in HNP- or HPNP-treated mice also exhibited obvious red fluorescence signals, which came from the sonosensitizer HP (Supplementary Fig. 10). These data validated that HNP and HPNP could passively accumulate at tumor sites due to their small size and the existence of the hydrophilic corona BSA, and the stronger tumor-targeting ability of HPNP than HNP was owing to the existence of aPD-L1.

To verify the sonodynamic activity of these NPs, the ^1^O_2_ generation was studied by detecting the green fluorescence signals from SOSG in tumor tissues. The sono-irradiation was conducted on the tumors at the maximum accumulation timepoint (12 h post-injection of NPs). The SOSG green fluorescence signals were detected in tumor tissues of HNP- or HPNP-injected and sono-irradiated mice (Supplementary Fig. 10). Their MFIs exhibited 7.5-fold and 10.5-fold increments relative to the unirradiated mice, respectively (Fig. 4d). These data confirmed the effective generation of ^1^O_2_ in tumor tissues of HNP- or HPNP-injected and sono-irradiated mice.

Afterward, the bilateral 4T1 tumor model was established to study the nano-immunocomplex-mediated sono-metabolic checkpoint trimodal cancer therapy (Fig. 4e). 4T1 cells were subcutaneously inoculated in the right flank of BALB/c mice as the primary tumor; 5 days later, the same amount of cells was subcutaneously inoculated in the left flank of mice as the distant tumor. After 2 days, 4T1 tumor-bearing mice were injected with mixed proteins (aPD-L1 and ADA), HNP, PNP, or HPNP and treated with or without sono-irradiation. Then, the growths of the primary and distant tumors and the survival of mice with different treatments were monitored. The primary and distant tumors in HPNP-injected and sono-irradiated mice were completely inhibited (Fig. 4f, g). The HNP-injected and sono-irradiated mice exhibited a short-term inhibition (14-day monitoring period) and an obvious tumor recurrence of the primary tumors with the prolonged monitoring period to 21 days. No obvious regression or inhibition of the primary or distant tumors was observed in the other treatment groups. Moreover, the HPNP-injected and sono-irradiated mice exhibited prolonged survivals relative to the mice with other treatments (Fig. 4h). The therapeutic effects were further confirmed by caspase-3 immunofluorescence and hematoxylin and eosin (H&E) staining analysis. The green fluorescence signals from the FITC-labelled anti-caspase-3 antibodies were detected in primary and distant tumors of HPNP-injected and sono-irradiated mice at 14 days post-injection (Supplementary Fig. 11). The MFIs were 31.7-fold and 37.9-fold higher than that of the unirradiated mice, respectively (Fig. 4i). In contrast, MFIs in primary tumors of HNP-injected and sono-irradiated mice exhibited a 32.9-fold increment relative to the unirradiated mice, while the distant tumors did not show obvious green fluorescence signals. Moreover, obvious dead cells were observed in both the primary and distant tumors of HPNP-injected and sono-irradiated mice in contrast to the other groups according to the H&E staining images (Supplementary Fig. 12). These were also consistent with the caspase-3 immunofluorescence staining results. To further confirm the inhibition of tumor metastasis by nano-immunocomplex-mediated therapy, the H&E staining of lung tissues from the mice with different treatments was conducted. The obvious metastatic niches were found in all groups except for the HPNP-injected and sono-irradiated mice at 22 days post-injection (Fig. 4j and Supplementary Fig. 13). As a result, these data validated that nano-immunocomplex-mediated therapy effectively inhibited tumor growth and metastasis, indicating its strong antitumor effects relative to the other treatments.

The bilateral CT26 tumor model was further established to study the nano-immunocomplex-mediated sono-metabolic checkpoint trimodal cancer therapy (Supplementary Fig. 14a). The experiment details were similar with the bilateral 4T1 tumor model. First, CT26 tumor cells suspended in RPMI 1640 cell culture medium were subcutaneously inoculated into the right flank (primary tumors) of each mouse (1×10^6^ cells/mouse). 5 days later, the same amounts of the cells were subcutaneously inoculated in the left flank (distant tumors) of the same mouse. Then CT26 tumor-bearing BALB/c mice were randomly divided into three groups (*n* = 5). The mice in each group were intravenously injected with 200 μL saline or PBS solutions containing HNP or HPNP. After 12 h post-injection, the primary tumor of each mouse was treated with sono-irradiation (1.0 MHz, 1.2 W/cm^2^, 50% duty cycle) for 6 min. The sizes of primary and distant tumors and the body weights of mice were measured every 2 days for 16 days. The primary and distant tumors in HPNP-injected and sono-irradiated mice were completely inhibited (Supplementary Figs. 14b, c and 15). However, both the primary and distant tumors of HNP-injected and sono-irradiated mice exhibited moderate inhibition effects. In addition, the body weights of mice with these treatments did not show obvious changes (Supplementary Fig. 14d). The H&E staining images of the major organs including heart, liver, lung, and kidney also did not show obvious damages at 16 days post-injection (Supplementary Fig. 16). This indicated the good biosafety and biocompatibility of HNP and HPNP on CT26 tumor-bearing mice. The caspase-3 immunofluorescence and H&E staining images further verified the antitumor effects of the nano-immunocomplex-mediated therapy (Supplementary Fig. 14e–g), which were consistent with the results on 4T1 tumor models.

The tumor rechallenge was further studied to evaluate the long-term antitumor and anti-recurrence effects of nano-immunocomplex-mediated sono-metabolic checkpoint trimodal cancer therapy (Fig. 4k). The 4T1 tumor-bearing mice that survived after multiple HNP or HPNP injections and sono-irradiation on day 40 were challenged with 4T1 cells on the other side of flanks (Supplementary Fig. 17). The tumor growths in HPNP-injected mice were greatly inhibited compared to that of the HNP-injected mice (Fig. 4l). The HPNP-injected mice also exhibited a prolonged survival relative to the HNP-injected mice (Fig. 4m). Moreover, the cancer-specific T-cell immunity was evaluated via the co-incubation of tumor (antigen-specific) or normal (antigen-nonspecific) cells with the isolated Teffs from the spleen of the rechallenged mice in vitro^36^. The extracted Teffs from the HNP-injected mice exhibited negligible cytotoxicity to both 4T1 tumor cells and 3T3 normal cells with cell viabilities of above 70% (Fig. 4n). In contrast, the cell viability of 4T1 cells after incubation with Teffs from HPNP-injected mice was only 40% at an E/T (effector T cells to target cells) ratio of 50 (Fig. 4o), which is much lower than that of 3T3 cells (89%), indicating the activation of specific antitumor immunity. These results validated that HPNP-mediated sono-metabolic checkpoint trimodal cancer therapy exhibited stronger specific antitumor and anti-recurrence effects compared to HNP-mediated therapy.

The biosafety of nano-immunocomplex-mediated sono-metabolic checkpoint trimodal cancer therapy was further evaluated by monitoring the body weight and histologically analyzing the major organs of mice. The body weights of mice with different treatments did not show obvious changes (Supplementary Fig. 18). The H&E staining images of the major organs including heart, liver, spleen, and kidney also did not show obvious damages at 14 days post-injection (Supplementary Fig. 19). This indicated the good biosafety and biocompatibility of these NPs.

### In vivo irAEs evaluation

The immune-related adverse events (irAEs) were studied in 4T1 tumor-bearing mice. 4T1 tumor-bearing mice were intravenously injected with saline, aPD-L1, or HPNP (injection dose: 200 μg per mouse aPD-L1). Then the major organs (including heart, liver, lung, and kidney) and sera were collected and analyzed after 2 days post-injection (Supplementary Fig. 20a). The major indexes for liver function including alanine transaminase (ALT) and aspartate aminotransferase (AST) in aPD-L1-inejcted mice exhibited obvious increase compared to that of saline- or HPNP-injected mice, indicating significant damages to the liver tissues (Supplementary Fig. 20b, c). The inflammatory cytokines (including tumor necrosis factor α (TNF-α), interferon-γ (IFN-γ), and interleukine-6 (IL-6)) in serum of aPD-L1-inejcted mice also exhibited obvious increase compared to that of saline- or HPNP-injected mice (Supplementary Fig. 20d–f). These results validated that aPD-L1 treatment could significantly induce the liver damage and elicit systemic inflammatory response, while our nano-immunocomplex effectively reduced the incidence of irAEs.

To deeply investigate irAEs after aPD-L1 treatment in 4T1 tumor-bearing mice, we detected the infiltrated immune cells (T cells and macrophages) and the inflammatory cytokines (TNF-α, IFN-γ, and IL-6) in major organs (including heart, liver, lung, and kidney) after 2 days post-injection. The populations of CD45^+^ leukocytes, CD45^+^CD3^+^ T cells, CD3^+^CD69^+^ activated T cells, F4/80^+^ macrophages, and F4/80^+^CD80^+^ M1-Macs in heart, liver, lung, or kidney from aPD-L1-injected mice significantly increased compared to the saline- or HPNP-injected mice (Supplementary Figs. 21–24). Meanwhile, the inflammatory cytokines (TNF-α, IFN-γ, and IL-6) in heart, liver, lung, or kidney exhibited significant increase after aPD-L1 treatment in 4T1 tumor-bearing mice relative to that of the saline or HPNP treatments (Supplementary Fig. 25). These results further verified that aPD-L1 treatment could lead to the infiltration of inflammatory immune cells and elevated cytokine levels in major organs of 4T1 tumor-bearing mice, which could not be found in HPNP-injected mice. Thus, our nano-immunocomplex exhibited excellent biosafety and effectively reduced the incidence of irAEs compared to the native aPD-L1.

### In vivo mechanistic studies of sono-metabolic checkpoint trimodal cancer therapy

**Fig. 5:**
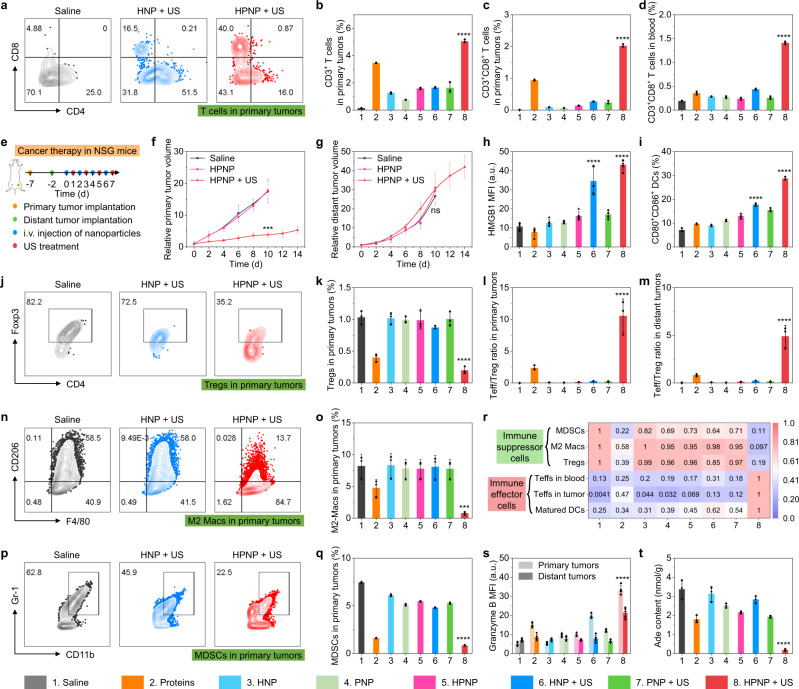
In vivo mechanistic study of nano-immunocomplex-mediated activatable sono-metabolic checkpoint trimodal cancer therapy. **a** Flow cytometry assay of tumor-infiltrating T lymphocytes (CD8^+^ and CD4^+^) and quantification of CD3^+^ T cells (**b**) and CD3^+^CD8^+^ Teffs (**c**) in primary tumors (*n* = 3). 8 versus other groups in **b** and **c**: *p* < 0.0001. **d** Flow cytometry quantification of CD3^+^CD8^+^ Teffs in blood (*n* = 3). 8 versus other groups: *p* < 0.0001. **e** Schematic illustration of the schedule for implantation and treatment of 4T1 tumor-bearing immunodeficient NSG mice. Growth curves of primary tumors (**f**) and distant tumors (**g**) after different treatments (*n* = 5). HPNP + US versus saline in **f**: *p* = 0.0002; HPNP + US versus saline in **g**: not significant (ns). **h** Quantification of HMGB1 expression in primary tumors (*n* = 5). 8 versus 5: *p* < 0.0001; 6 versus 3: *p* < 0.0001. **i** Flow cytometry quantification of matured DCs (CD80^+^CD86^+^) in TDLNs (*n* = 3). 8 versus 5: *p* < 0.0001; 6 versus 3: *p* < 0.0001. Flow cytometry assay (**j**) and quantification (**k**) of CD4^+^Foxp3^+^ Tregs in primary tumors (*n* = 3). 8 versus 1: *p* < 0.0001. Quantification of Teff/Treg ratio in primary (**l**) and distant (**m**) tumors (*n* = 3). 8 versus other groups in **l** and **m**: *p* < 0.0001. Flow cytometry assay and quantification of F4/80^+^CD206^+^ M2 Macs (**n** and **o**) and CD11b^+^Gr-1^+^ MDSCs (**p** and **q**) in primary tumors (*n* = 3). 8 versus 1 in **o**: *p* = 0.0004; 8 versus 1 in **q**: *p* < 0.0001. **r** The relative populatio*n*s of immune effector cells and immune suppressor cells after different treatments (*n* = 3). **s** Quantification of granzyme B expression in primary tumors (*n* = 5). 8 versus 5 in primary and distant tumors: *p* < 0.0001. **t** The Ade content in primary tumors after different treatments (*n* = 3). 8 versus other groups: *p* < 0.0001. Injection dose: 200 μL, [HP] = 1 mmol/L, or [ADA] = 40 U/mL; sono-irradiation: 1.0 MHz, 1.2 W/cm^2^, 50% duty cycle for 6 min. Statistical significance in **b**–**d**, **h**, **i**, **k**–**m**, **o**, **q**, **s**, and **t** was calculated via one-way ANOVA with a Tukey post-hoc test. Statistical significance in **f** and **g** was calculated via a two-tailed Student’s *t*-test. ***p < 0.001, and ****p < 0.0001. The mean values and SD are presented. Source data are provided as a Source Data file.

To study the mechanism of nano-immunocomplex-mediated sono-metabolic checkpoint trimodal cancer therapy, Teffs were first detected by collecting the primary and distant tumor tissues, blood, and spleens by flow cytometry analysis. The populations of CD3^+^ tumor-infiltrating T lymphocytes (TILs), Teffs (CD8^+^), and activated Teffs (CD8^+^CD69^+^) in primary and distant tumors of HPNP-injected mice were higher than that of proteins-, HNP-, or PNP-injected mice after sono-irradiation (Fig. 5a–c, Supplementary Figs. 26–30). The frequencies and activation of Teffs in blood and spleen of HPNP-injected mice also increased relative to that of proteins-, HNP-, or PNP-injected mice after sono-irradiation (Fig. 5d, Supplementary Figs. 31, 32). These results indicated that nano-immunocomplex-mediated therapy greatly enhanced antitumor Teffs immunity. To further verify the important roles of Teffs in nano-immunocomplex-mediated antitumor immunity, the antitumor effects were evaluated in 4T1 tumor-bearing immunodeficient NOD-Scid *IL2rg*^*−/−*^ (NSG) mice, which lack functional lymphocytes (Fig. 5e). The growths of primary tumors in HPNP-injected and sono-irradiated mice were partially inhibited owing to the sonodynamic antitumor activity of HPNP, while the distant tumors exhibited negligible inhibition effects compared to that of the unirradiated mice (Fig. 5f, g, Supplementary Fig. 33). These data validated that nano-immunocomplex-mediated therapy was dependent on the acidic TME/sono-activation of Teff-mediated antitumor immunity.

To gain insight into the high antitumor Teffs immunity of nano-immunocomplex-mediated therapy, the corresponding immunological processes including ICD induction, DC maturation, and cytokine release of NPs-injected 4T1 tumor-bearing mice were investigated and compared. The green fluorescence signals from FITC-labeled anti-HMGB1 antibodies were detected in primary tumors of HNP- or HPNP-injected and sono-irradiated mice at 7 days post-injection, which exhibited a similar 2.7-fold increase relative to that of the unirradiated mice (Fig. 5h, Supplementary Fig. 34a). DC maturation was also studied by detecting the population of matured DCs (CD80^+^CD86^+^) in tumor-draining lymph nodes (TDLNs) by flow cytometry analysis. The frequency of matured DCs in HNP- or HPNP-injected and sono-irradiated mice increased by 2.0- or 2.2-fold compared to that of the unirradiated mice (Fig. 5i, Supplementary Fig. 35). The inflammatory cytokines of the serum were further detected via ELISA at 5, 7, or 14 days post-injection. Both the serum concentration of IL-6 and TNF-α in HPNP-injected and sono-irradiated mice reached maximum values at 7 days post-injection, which increased by 11.6- and 23.6-fold relative to the unirradiated mice, respectively (Supplementary Fig. 34b, c). These results confirmed that HNP- or HPNP-mediated therapy enhanced tumor immunogenicity to a similar level via inducing ICD, promoting DC maturation and antigen presentation, and stimulating inflammatory cytokines release owing to their analogical sonodynamic activity.

Afterward, the immunosuppressive TME was studied to explore the difference between HNP- and HPNP-mediated therapy. The immune suppressor cells including Tregs, M2 Macs, and MDSCs play key roles in developing immunosuppressive TME against antitumor Teffs immunity^37^, which were analyzed in this study. The populations of Tregs (CD4^+^Foxp3^+^) in primary and distant tumors of HPNP-injected and sono-irradiated mice decreased by 80.6% and 72.5% compared to that of the saline-injected mice (Fig. 5j, k, Supplementary Figs. 36, 37). Notably, the ratios of Teff to Treg in primary and distant tumors of HPNP-injected and sono-irradiated mice were ~10.6 and ~4.9, which exhibited 75.5- and 37.7-fold increments compared to that of the unirradiated mice, respectively (Fig. 5l, m). Moreover, the populations of the immunosuppressive M2 Macs and MDSCs in primary tumors of HPNP-injected and sono-irradiated mice were lower than the mice in other groups, which showed 9.3- and 7.9-fold decreases relative to that of the saline-injected mice, respectively (Fig. 5n–q, Supplementary Figs. 38–41). The amounts of M2 Macs and MDSCs exhibited similar declining trends in distant tumors of HPNP-injected and sono-irradiated mice. The slight decreases of these immune suppressor cells (including Tregs, M2 Macs, and MDSCs) and increases of immune effector cells (Teffs) were also found in primary and distant tumors of proteins-injected mice. This is because aPD-L1-mediated ICB and ADA-mediated IMT can partially improve the activity of Teffs and reprogram immunosuppressive TME. The relative populations of immune effector cells (including matured DCs, Teffs in tumor, and Teffs in blood) and immune suppressor cells (including Tregs, M2 Macs, and MDSCs) further validated that nano-immunocomplex-mediated therapy could leverage the immune balance to the antitumor orientation (Fig. 5r). As a result, the expression of granzyme B could be obviously detected in primary and distant tumors of HPNP-injected and sono-irradiated mice, and the MFIs were 3.4- and 3.0-fold higher than that of the unirradiated mice, respectively (Fig. 5s, Supplementary Fig. 42). Then the aPD-L1 release in tumor tissues was studied via immunofluorescence staining to verify the aPD-L1-mediated ICB. The immunofluorescence staining images of the tumor tissues in HPNP-injected and sono-irradiated mice exhibited obvious green fluorescence signals, indicating the effective release of aPD-L1 upon sono-irradiation (Supplementary Fig. 43). To further confirm the immunosuppressive TME reprogramming by IMT, the Ade content and immunosuppressive cyclic adenosine 3′,5′-monophosphate (cAMP) level in tumor tissues were investigated via HPLC analysis and ELISA, respectively^38^. After injection of HPNP and subsequent sono-irradiation, the Ade contents in primary tumors decreased by 94.5%, 91.3%, and 89.6% relative to that of the saline-injected, unirradiated, and proteins-injected mice, respectively (Fig. 5t). Meanwhile, the cAMP levels in primary tumors of HPNP-injected and sono-irradiated mice exhibited obvious decreases relative to that of the saline-injected (69.0%), unirradiated (62.5%), and proteins-injected (37.9%) mice (Supplementary Fig. 44). These results indicated that nano-immunocomplex could induce more effective Ade and cAMP depletion than the other groups via acidic TME/sono-activation of IMT, leading to better immunosuppressive TME reprogramming and stronger antitumor immunity. Although the intratumoral Ino contents should increase with the continuous catalytic conversion of Ade by ADA, the exact mechanism of Ino action on the immune system is still under investigation. Prior data supports an immunosuppressive role for Ino, which can activate A2AR signaling to inhibit inflammation and antitumor immunity^39^. However, inosine supplementation was recently reported to enhance the antitumor efficacy of checkpoint blockade therapy in specific mouse tumors that unable to catabolize Ino to support cell growth^40^; Mager et al. also demonstrated context-dependent actions of inosine on T cell immunity, which boosted or inhibited Th1 differentiation of naive T cells in the presence or absence of exogenous interferon-γ, respectively^41^. Thus, whether the inosine action is relevant to antitumor immunity deserves scrutiny.

To further study the mechanism of nano-immunocomplex-mediated sono-metabolic checkpoint trimodal cancer therapy on CT26 tumor-bearing mice, CD8^+^ Teffs were first detected by collecting the primary and distant tumor tissues, blood, and spleens by flow cytometry analysis. The populations of activated CD69^+^ T cells in primary and distant tumors, blood, and spleens of HPNP-injected mice were obviously higher than that of HNP-injected mice after sono-irradiation (Supplementary Fig. 45). These results confirmed that nano-immunocomplex-mediated therapy greatly enhanced antitumor Teffs immunity, which were consistent with that on 4T1 tumor models. Then the corresponding immunological processes including ICD induction and DC maturation were investigated and compared. The green fluorescence signals from FITC-labeled anti-HMGB1 antibodies were detected in primary tumors of HNP- or HPNP-injected and sono-irradiated mice at 7 days post-injection, which exhibited 2.1- and 2.5-fold increases relative to that of the unirradiated mice, respectively (Supplementary Fig. 46a, b). DC maturation was further studied by detecting the population of matured DCs (CD80^+^CD86^+^) in TDLNs by flow cytometry analysis. The frequency of matured DCs in HNP- or HPNP-injected and sono-irradiated mice increased by 1.2- or 2.0-fold compared to that of the unirradiated mice (Supplementary Fig. 46c).

Afterward, the immunosuppressive TME was studied to investigate the mechanism of nano-immunocomplex-mediated cancer therapy. The populations of Tregs (CD4^+^Foxp3^+^) in primary and distant tumors of HPNP-injected and sono-irradiated mice decreased by 68.1% and 63.1% compared to that of the saline-injected mice, respectively (Supplementary Fig. 47a). However, HNP-treated group only exhibited moderate decreases of Tregs in primary (33.5%) and distant (38.9%) tumors relative to that of the saline-injected mice. Moreover, the populations of the immunosuppressive M2 Macs and MDSCs in primary tumors of HPNP-injected and sono-irradiated mice were lower than the mice in other groups, which decreased by 26.3% and 24.9% relative to that of the saline-injected mice, respectively (Supplementary Fig. 47b, c). Meanwhile, the amounts of M2 Macs and MDSCs exhibited similar declining trends in distant tumors of HPNP-injected and sono-irradiated mice. The expression of granzyme B exhibited an obvious increase in primary and distant tumors of HPNP-injected and sono-irradiated mice (Supplementary Fig. 47d). The MFIs were 3.2- and 5.4-fold higher than that of the saline-injected mice, respectively. These results indicated that nano-immunocomplex could induce more effective antitumor immune responses than the other groups via enhancing tumor immunogenicity and reprogramming immunosuppressive TME in CT26 tumor model, which were consistent with that of 4T1 tumor model.

**Fig. 6:**
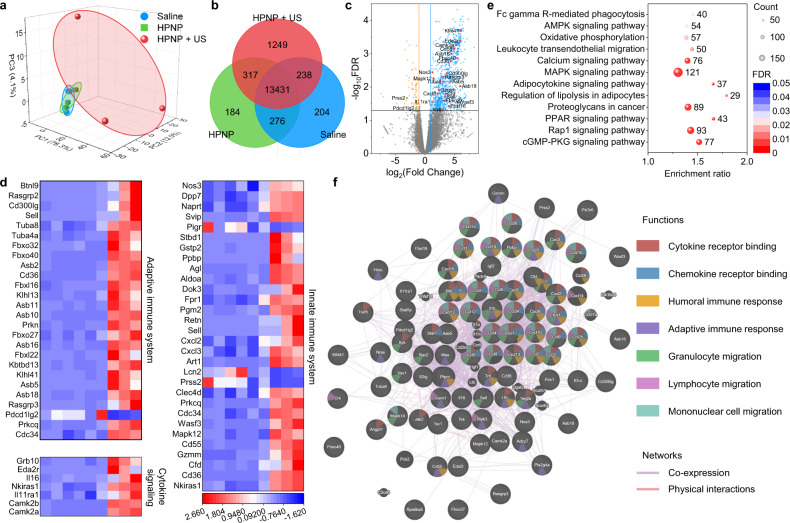
Transcriptome analysis of nano-immunocomplex-mediated sono-metabolic checkpoint trimodal cancer therapy. **a** Principal component analysis (PCA) score plot of the expressed genes in TME of saline-injected, HPNP-injected, or HPNP-injected and sono-irradiated mice (*n* = 3). **b** Venn diagram of the identified differentially expressed genes. **c** Volcano plot showing 938 differentially expressed genes (26703 total genes) in TME from HPNP-injected and sono-irradiated mice compared to TME from saline-injected mice. 866 upregulated genes and 72 downregulated genes were differentially expressed in TME in HPNP-injected and sono-irradiated mice. **d** Expression of selected genes related to the adaptive immune system, cytokine signaling, and innate immune system in TME from HPNP-injected and sono-irradiated mice compared to TME from saline-injected mice. **e** KEGG enrichment analysis of the identified differentially expressed genes for studying the pathways of immune responses. **f** GeneMANIA analysis for predicting gene interactions between differential genes in the immunological processes. Source data are provided as a Source Data file.

To deeply investigate the impact of nano-immunocomplex-mediated therapy on TME, the transcriptome landscape of the tumor tissues at 14 days post-injection was profiled. A total of 26703 genes were identified. Nano-immunocomplex (HPNP-injected and sono-irradiated groups) treatment promoted a dramatic shift of the transcription program in TME relative to the saline-injected or unirradiated HPNP-injected groups. Both the principal component analysis (PCA) and Venn diagram indicated the significant discrepancy of the transcriptome landscape between the nano-immunocomplex and saline treatment groups (Fig. 6a, b). Compared to the saline treatment, 938 differentially expressed genes were found under a threshold with absolute fold changes >2 and *p* values < 0.05 after nano-immunocomplex treatment. Specifically, 866 upregulated genes and 72 downregulated genes were identified in TME in nano-immunocomplex treatment relative to saline treatment (Fig. 6c). Thereafter, these differentially expressed genes associated with immune functions were sorted out (Fig. 6d). Nano-immunocomplex treatment induced the upregulated expression of genes associated with the adaptive immune system (for example, *Klhl41* and *Asb18* for antigen presentation; *Cd300lg* and *Sell* for immunoregulatory interactions; *Cdc34* and *Prkcq* for T cell receptor signaling), cytokine signaling (including *Camk2a* and *Camk2b* for interferon signaling; *Il16* and *Il11ra1* for interleukin signaling; *Eda2r* and *TNFR2* for non-canonical NF-kB (nuclear factor kappa-light-chain-enhancer of activated B cells) pathway), and innate immune system (for instance, *Wasf3* for Fcγ receptor (FCGR) dependent phagocytosis; *Cd55* and *Cfd* for complement cascade; *Cd36* and *Nkiras1* for Toll-like receptor cascades). Moreover, immunosuppressive genes (such as *Pdcd1lg2* for negative regulation of the adaptive immune response) were downregulated after nano-immunocomplex treatment. Afterward, Kyoto Encyclopedia of Genes and Genomes (KEGG) pathway enrichment analysis of these differentially expressed genes validated that several immune-associated signaling pathways (for example, mitogen-activated protein kinase (MAPK) signaling pathway for regulating Th1- and Th2-type immune responses; Ras-associated protein 1 (Rap1) signaling pathway for regulating T cell functions; Fc gamma R-mediated phagocytosis signaling pathway for regulating innate immune system) were obviously affected after nano-immunocomplex treatment (Fig. 6e). To further predict the functional interactions of these differentially expressed genes in immunological processes, GeneMANIA, a multiple association network integration algorithm, was performed^42^ (Fig. 6f). The complex gene networks indicated the co-expression of these differentially expressed genes, followed by physical interactions. GeneMANIA analysis of the gene sets associated with cytokine receptor binding (45 detected genes of 287 genes), chemokine receptor binding (36 of 71), humoral immune response (28 of 199), adaptive immune response (18 of 294), granulocyte migration (40 of 153), lymphocyte migration (31 of 99), and mononuclear cell migration (26 of 86) further confirmed that multiple immunological processes were activated and interacted with each other to promote antitumor immunity. As a result, these transcriptome data provided evidence that nano-immunocomplex-mediated therapy had the ability to promote a cascade of transcriptional events in multiple immunological processes to reshape immunosuppressive TME and activate antitumor immunity.

## Discussion

Precision combinational immunotherapy is a highly desired cancer therapeutic modality to increase patient response rate and minimize irAEs, but it remains lacking. Our nano-immunocomplex represents an example of catalytical nanomedicine whose sonodynamic, checkpoint blockade, and immunometabolic reprogramming activities are dual-locked and can be remotely activated by sono-irradiation in TME. As traditional sono-irradiation strategy utilizes micro-/nanobubble loaded nanoparticles to induce instant drug burst at tumor site via US-mediated cavitation^22^, it still encounters nonspecific releases at normal tissue and thus off-target toxicity. In contrast, our design addresses this issue by covalently immobilizing the therapeutic agents into one entity via acid-cleavable and ^1^O_2_-activatable linkers followed by precise sonodynamic scission. This covalently immobilized nanostructure was found to silence the activities of both aPD-L1 and ADA in physiological conditions, which however can be awakened in acidic TME under sono-irradiation: increased PD-L1 blockade efficiency from 45 to 99% and Ade degradation efficiency from 17 to 79% (Fig. 2e–h). Notably, all the major components (HP, aPD-L1, and ADA) of the nano-immunocomplex are FDA-approved, natural-derived, or self-originated, which ensures the high biosafety and in turn high clinical translation potential.

Enzymatic therapy has been utilized to afford more durable response than small-molecule drugs for a broad range of diseases^43–45^. Our nano-immunocomplex represents a remote-controlled enzymatic nanomedicine to reprogram the immunosuppressive TME precisely and persistently for cancer immunotherapy. Although several phase I/phase II clinical trials combining Ade blockade therapy with chemotherapy or ICB have been launched (e.g., NCT03549000, NCT03884556, NCT02754141, NCT02740985, etc.)^46,47^, preclinical results demonstrated their limited therapeutic efficacy in mouse models^48^. The major reason is the existence of multiple Ade production sources (including the CD39/CD73 axis, alkaline phosphatase, and prostatic acid peptidase) and diverse purinergic receptors (A2AR and A2BR)^25^. By virtue of the durable conversion of Ade to Ino via the catalysis of ADA, our nano-immunocomplex can potently and completely deplete Ade to eliminate a spectrum of immunosuppressive adenosinergic signaling pathways. It is validated by the higher Ade depletion efficiency (ca. 94%) of the nano-immunocomplex relative to that of free proteins (ca. 47%) and unirradiated group (ca. 36%) in 4T1 tumor mouse model (Fig. 5t). Notably, this Ade depletion efficiency (ca. 99%) in 4T1 cells with additional Ade is also higher than the reported studies using various anti-CD39/CD73 antibodies (ca. 60%) in vitro^49^. Such a durable and broad elimination of adenosinergic signaling pathways also leads to 10-times lower dosage (ADA, 2 mg/kg) for the nano-immunocomplex relative to that of the reported studies on anti-CD39/CD73 antibodies (10–20 mg/kg). In comparison with bimodal therapies with limited immunomodulation, the trimodal therapy based on adenosinergic signaling elimination in combination with SDT and ICB modulates the whole immunological processes (including ICD induction, TAAs release, DC maturation, immunosuppressive TME reprogramming, and immune effector cells activation). Thus, the trimodal therapy (nano-immunocomplex treatment) promotes obvious increments of immune effector cells (1.9- and 1.1-folds for matured DCs and effector TILs respectively, Fig. 5a–c, i) and remarkable decreases of immune suppressor cells (51.3, 83.3, and 50.0% for Tregs, M2 Macs and MDSCs respectively, Fig. 5j–q) compared to the bimodal therapy (synergistic aPD-L1 and ADA treatment), leading to the effective inhibition of tumor growth, metastasis, and recurrence (Fig. 4).

In summary, we report a catalytical nano-immunocomplex equipped with cancer-specific and sono-activatable activities for precision sono-metabolic checkpoint trimodal cancer therapy. Apart from its easy nano-formulation and FDA-approved safe composition, the nano-immunocomplex possesses long-desired advantages including whole immunological modulation, durable and amplified reprogramming of immunosuppressive TME, and dual-lock induced tumor specificity, holding a high promise for translation. Such an effective design can be generalized for precise intratumoral modulation of other immune-associated metabolic pathways (such as glycolysis, glutaminolysis, hypoxia, lipid metabolism, etc.)^50,51^, permitting precision multimodal cancer immunotherapy.

## Methods

### Chemicals

All the chemicals were purchased from Sigma-Aldrich unless otherwise specific indicated. Hematoporphyrin was purchased from MedChemExpress. Anti-mouse PD-L1 antibodies were purchased from Bio X Cell. Trypsin–EDTA (0.05%), penicillin–streptomycin (10,000 U/mL), fetal bovine serum (FBS), RPMI 1640 medium, ACK lysis, type I collagenase, and type IV collagenase were purchased from Gibco. The mouse PD-1[Biotinylated]:PD-L1 inhibitor screening assay kit was purchased from BPS Bioscience, Inc. Mouse granulocyte macrophage-colony-stimulating factor (GM-CSF) was purchased from i-DNA Biotechnology Pte Ltd. SOSG was purchased from Molecular Probes Inc. (Carlsbad, CA, USA). 3-(4,5-Dimethylthiazol-2-yl)-5-(3-carboxymethoxyphenyl)-2-(4-sulfophenyl)-2H-tetrazolium, inner salt (MTS) solution was purchased from Promega Corp. (Madison, WI, USA). Granzyme B antibody (ab255598, dilution: 1:200) and secondary antibody Alexa Fluor 488 conjugated goat anti-rabbit IgG H&L (ab150077, dilution: 1:500) were purchased from Abcam Inc. (Cambridge, CA, USA). ELISA kits for cAMP detection, HMGB1 antibody (Catalog no. 3935 S, dilution: 1:100), and cleaved caspase-3 antibody (Catalog no. 9661 L, dilution: 1:500) were purchased from Cell Signaling Technology. ELISA kits for IL-6 and TNF-α detection, Zombie UV™ fixable viability kit (Catalog no. 423108, dilution: 1:500), purified anti-mouse CD16/32 (Catalog no. 156604, dilution: 1:200), AF700 anti-mouse CD45 antibody (Catalog no. 147716, dilution: 1:200), FITC anti-mouse CD3 (Catalog no. 100204, dilution: 1:50), APC anti-mouse CD8a (Catalog no. 100712, dilution: 1:80), PE anti-mouse CD4 (Catalog no. 130310, dilution: 1:80), BV605 anti-mouse CD69 (Catalog no. 104530, dilution: 1:40), APC anti-mouse CD11c (Catalog no. 117310, dilution: 1:80), FITC anti-mouse CD80 (Catalog no. 104706, dilution: 1:50), PE anti-mouse CD86 (Catalog no. 105008, dilution: 1:20), PerCP anti-mouse CD4 (Catalog no. 100432, dilution: 1:80), AF647 anti-mouse Foxp3 (Catalog no. 126408, dilution: 1:50), PE anti-mouse CD11b (Catalog no. 101208, dilution: 1:80), AF488 anti-mouse F4/80 (Catalog no. 123120, dilution: 1:50), AF647 anti-mouse CD206 (Catalog no. 141712, dilution: 1:100), BV605 anti-mouse Gr-1 (Catalog no. 108440, dilution: 1:40), and were purchased from Biolegend.

### Material characterization

Electrospray ionization-mass spectrometry (ESI-MS) spectra were conducted with a ThermoFinnigan LCQ quadrupole ion trap mass spectrometer (Thermo Fisher Corporation) equipped with a standard ESI source. Proton nuclear magnetic resonance (^1^H NMR) spectra were conducted on a Bruker BBFO 400 MHz system (Bruker Physik AG, Germany). TEM images were captured using a JEM 1400 transmission electron microscope (JEOL, Tokyo, Japan). DLS and Zeta potential measurements were performed on a Malvern Nano-ZS Particle Sizer (Malvern Instruments, Southborough, UK). Absorption and fluorescence spectra were measured on a UV-2450 spectrophotometer (Shimadzu, Japan) and a Fluorolog 3-TCSPC spectrofluorometer (Horiba Jobin Yvon), respectively. HPLC analyses and purification were performed on an Agilent 1260 system using methanol (MeOH)/water (H_2_O) as the eluent. Confocal images were captured using an LSM800 confocal laser scanning microscope (Carl Zeiss, Germany). Flow cytometry assay was performed on Fortessa X20 (BD Biosciences) and analyzed by FlowJo v10. The absorbance or chemiluminescence intensities of each well in a 96-well plate were measured using a SpectraMax M5 microplate reader. In vivo animal fluorescence images were captured using an IVIS imaging system (IVIS-CT machine, PerkinElmer). Tissues were cut into sections using a cryostat (Leica). The tissue sections were examined on a Nikon ECLIPSE 80i microscope (Nikon Instruments). NMR spectra were analyzed using Mestre Nova LITE v5.2.5-4119 software (Mestre lab Research S.L.).

### Synthesis of HP-NH_2_

HP was purchased from MedChemExpress company and characterized by ^1^H NMR. ^1^H NMR (DMSO-d_6_, 400 MHz): δ 10.49–11.00 (m, 4H), 6.59 (d, *J* = 3.6 Hz, 2H), 4.41 (s, 4H), 3.67–3.76 (m, 12H), 3.19 (t, *J* = 6.6 Hz, 4H), 2.07 (s, 6H).

Then, a mixture of HP (335.81 mg, 0.5 mmol), o-benzotriazole-N,N,N’,N’-tetramethyluroniumhexafluoro-phosphate (HBTU, 455.10 mg, 1.2 mmol), 1-hydroxybenzotriazole (HOBt, 162.16 mg, 1.2 mmol), diisopropylethylamine (DIPEA, 198.3 μL, 1.2 mmol), and *N*-Fmoc-ethylenediamine hydrobromide (435.90 mg, 1.2 mmol) was dissolved in DMF under N_2_ atmosphere and stirred at room temperature for 6 h. Purification of the residue by silica gel column chromatography gave the compound HP-NHFmoc (purple solid, 461.9 mg, yield 82%). ^1^H NMR (DMSO-d_6_, 400 MHz): δ 10.35–10.85 (m, 4H), 7.77 (t, *J* = 6 Hz, 4H), 7.54 (t, *J* = 6.8 Hz, 4H), 7.29–7.34 (m, 4H), 7.20–7.24 (m, 4H), 6.54 (s, 2H), 6.54 (s, 2H), 4.36–4.37 (m, 4H), 4.07–4.15 (m, 8H), 3.62–3.72 (m, 12H), 3.06 (t, *J* = 6.2 Hz, 4H), 2.95–2.97 (m, 4H), 2.70–2.75 (m, 2H), 2.11–2.16 (m, 6H). ESI (m/z): calcd for C_68_H_7_0N_8_O_8_, 1126.5 [M]; found, 1149.6 [M + Na]^+^.

The obtained HP-NHFmoc (60 mg, 0.053 mmol) was further stirred in 4 mL of piperidine/DMF (20%, V/V) solution at room temperature for 2 h. Purification of the residue by HPLC using methanol/water as eluents gave the compound HP-NH_2_ (purple solid, 18.8 mg, yield 52%). ^1^H NMR (DMSO-*d*_*6*_, 400 MHz): δ 10.25–10.76 (m, 4H), 6.51–6.56 (m, 2H), 6.54 (s, 2H), 4.23–4.44 (m, 12H), 3.67–3.71 (m, 12H), 3.00–3.08 (m, 4H), 2.95–2.97 (m, 4H), 2.14–2.18 (m, 6H). ESI (m/z): calcd for C_38_H_50_N_8_O_4_, 682.4 [M]; found, 342.1 [1/2(M + 2H)]^+^.

### Synthesis of thioketal-NHS

A mixture of mercaptoacetic acid (9.2 g, 100 mmol), acetone (3.2 g, 55 mmol), and 20 μL trifluoroacetic acid (TFA) was stirred at 0 °C for 3 h. The reaction mixture was washed by diethyl ether for three times to give the compound thioketal-COOH (white solid 21.1 g, yield 95%). ^1^H NMR (DMSO-*d*_*6*_, 400 MHz): δ 12.60 (s, 2H), 3.56 (s, 4H), 1.53 (s, 6H). ESI (m/z): calcd for C_7_H_12_O_4_S_2_, 224.0 [M]; found, 225.1 [M + H]^+^.

Then, a solution of obtained thioketal-COOH (2.5 g, 11.1 mmol) in anhydrous tetrahydrofuran (THF, 60 mL) was slowly added with NaBH_4_ (2.5 g, 66 mmol) in an ice bath. Subsequently, I_2_ (10 g, 28.5 mmol) in anhydrous THF (50 mL) were added dropwise in the mixture and was heated to reflux for 24 h and cooled to room temperature. Methanol (~40 ml) was added dropwise until the reaction became clear. The organic solvent was removed by vacuum pump and the residue was directly purified by silica gel column chromatography using dichloromethane/ethyl acetate to give compound thioketal-OH (colorless liquid, 1.2 g, yield 55%).^1^H NMR (DMSO-*d*_*6*_, 400 MHz): δ 3.52 (*t*, *J* = 7.2 Hz, 4H), 2.65 (*t*, *J* = 7.0 Hz, 4H), 1.52 (s, 6H). ESI (m/z): calcd for C_7_H_16_O_2_S_2_, 196.1 [M]; found, 197.0 [M + H]^+^.

The mixture of triethylamine (2.13 mL, 15.3 mmol) and obtained thioketal-OH (1 g, 5.1 mmol) in anhydrous THF (15 mL) was added dropwise into a solution of triphosgene (4.54 g, 15.3 mmol) in anhydrous THF (10 mL) at 0 ^o^C under N_2_ atmosphere. Then, the mixture was stirred for 4 h and monitored by TLC. After completing the reaction, *N*-hydroxy succinimide (NHS, 1.76 g, 15.3 mmol) in anhydrous THF (20 mL) and triethylamine (2.13 mL, 15.3 mmol) were added into the reaction followed by the removal of excess triphosgene. Then, the reaction was stirred for 6 h at 0 ^o^C. The solvent was removed under vacuum and directly purified by flash silica gel column chromatography to give obtained the compound thioketal-NHS (white solid, 778 mg, yield 32%). ^1^H NMR (CDCl_3_, 400 MHz): δ 4.57 (m, 4H), 3.76 (m, 4H), 2.84 (s, 8H), 1.63 (s, 6H). ESI (m/z): calcd for C_17_H_22_N_2_O_10_S_2_, 478.1 [M]; found, 500.1 [M + Na]^+^.

### Synthesis of the nano-immunocomplex

A mixture of HP-NH_2_ (500 equiv.), aPD-L1 (1 equiv.), and ADA (2.5 equiv.) in 2 M sodium chloride solution was added slowly with thioketal-NHS (1000 equiv.) and stirred overnight at 4 °C. Then, BSA (20 equiv.) and GA (500 equiv.) were added into the solution and stirred at 4 °C for another 12 h. The obtained nano-immunocomplex was purified by washing thrice with PBS to remove free crosslinkers and proteins using 300k MWCO centrifugal concentrator (ThermoFisher). Two control nanoparticles (HNPs and PNPs) were synthesized via similar methods.

### In vitro sonodynamic studies

PBS solutions (1 mL) containing HNP, PNP, or HPNP ([HP] = 20 μmol/L, [ADA] = 800 U/L, or [aPD-L1] = 6 mg/L) were mixed with 1 μL SOSG probe (500 μM). Then the solutions were treated with sono-irradiation (1.0 MHz, 1.2 W/cm^2^, 50% duty cycle) for 8 min. The fluorescence intensities at 520 nm were then recorded every 1 min during sono-irradiation using a Fluorolog 3-TCSPC spectrofluorometer termed as F. The fluorescence intensities at 520 nm without sono-irradiation were also recorded as F_0_. Finally, the increments of fluorescence intensities of each sample were calculated as F/F_0_.

### In vitro ADA activity studies

The PBS solutions (1 mL, pH = 7.4 or 6.8) containing HPNP ([HP] = 1 μmol/L, [ADA] = 40 U/L, or [aPD-L1] = 0.3 mg/L) were treated with sono-irradiation (1.0 MHz, 1.2 W/cm^2^, 50% duty cycle) for 6 min, followed by incubation with Ade (20 mmol/L) for 12 h. Then the solutions were collected at different timepoints and further diluted for HPLC analysis to quantify the Ade and Ino contents.

### In vitro PD-L1 blockade efficiency assay

The PD-L1 blockade experiments were conducted by using the mouse PD-1[Biotinylated]:PD-L1 inhibitor screening assay kit according to the manufacture’s protocols. First, the PBS solutions (1 mL, pH = 7.4 or 6.8) containing HPNP ([HP] = 1 μmol/L or [ADA] = 40 U/L) were treated with sono-irradiation (1.0 MHz, 1.2 W/cm^2^, 50% duty cycle) for 6 min. Then the solutions together with mouse PD-1-biotin were added into the mouse PD-L1-precoated 96-well plate and incubated at room temperature for 2 h. Afterward, streptavidin-HRP was added into each well and incubated for 1 h at room temperature. Finally, the PD-L1 blockade efficiency was further calculated by detecting the chemiluminescent intensity of each well after adding the ELISA ECL substrate.

### In vitro cellular uptake assay

4T1 and CT26 cancer cells or 3T3 normal cells were purchased from American Type Culture Collection (ATCC) and cultured in RPMI 1640 or DMEM cell culture medium supplemented with 10% FBS and 1% antibiotics (penicillin−streptomycin, 10,000 U/mL) at 37 °C and 5% CO_2_. The cells were seeded into confocal cell culture dishes at a density of 5 × 10^4^ cells/dish and cultured overnight for cell attachment. Then the cells were incubated with HNP, PNP, or HPNP ([HP] = 20 μmol/L, [ADA] = 800 U/L, or [aPD-L1] = 6 mg/L) for 12 h. After washing thrice with PBS to remove free nanoparticles, the cells were stained with Hoechst 33342, and the fluorescence images of cells were captured using an LSM800 confocal laser scanning microscope (Carl Zeiss, Germany).

### In vitro lysosome colocalization assay

4T1 cells were seeded in confocal cell culture dishes at a density of 5 × 10^4^ cells/dish and cultured overnight for cell attachment. Then the cells were incubated with HNP, PNP, or HPNP ([HP] = 20 μmol/L, [ADA] = 800 U/L, or [aPD-L1] = 6 mg/L) for 6 h. After washing thrice with PBS to remove free nanoparticles, the cells were stained with Hoechst 33342 and the lysosome tracker (Green DND-26), and the fluorescence images of cells were captured using an LSM800 confocal laser scanning microscope (Carl Zeiss, Germany).

### In vitro Ade content measurement

4T1 cancer cells were seeded in 6-well cell culture plates at a density of 3 × 10^5^ cells/well and then cultured overnight for cell attachment. Then the cells were treated with free Ade (10 mmol/L), followed by incubation with HNP, PNP, or HPNP ([HP] = 1 μmol/L, [ADA] = 40 U/L, or [aPD-L1] = 0.3 mg/L) for 4 h. After treated with or without sono-irradiation (1.0 MHz, 1.2 W/cm^2^, 50% duty cycle for 6 min), the cells were further incubated for 8 h. Then the supernatant in each well was collected for HPLC analysis to quantify the Ade and Ino contents.

### Intracellular ROS generation measurement

4T1 cancer cells were seeded in confocal cell culture dishes at a density of 5 × 10^4^ cells/dish and then cultured overnight for cell attachment. Then the cells were incubated with HNP, PNP, or HPNP ([HP] = 20 μmol/L, [ADA] = 800 U/L, or [aPD-L1] = 6 mg/L) for 12 h. Afterward, the cells were incubated with the H_2_DCFDA probe for 30 min, followed by sono-irradiation (1.0 MHz, 1.2 W/cm^2^, 50% duty cycle) for 6 min. The cells were then washed thrice with PBS, and the fluorescence images of cells were captured using an LSM800 confocal laser scanning microscope (Carl Zeiss, Germany).

### In vitro cell viability assay

4T1 cancer cells were seeded in 96-well cell culture plates at a density of 1 × 10^4^ cells/well and then cultured overnight for cell attachment. Then the cells were incubated with HNP, PNP, or HPNP at different concentrations ([HP] = 1, 2, 4, 6, and 8 μmol/L or [ADA] = 40, 80, 160, 240, or 320 U/L) for 12 h. The cells without NPs incubation were used as control. Then the cells were treated with or without sono-irradiation (1.0 MHz, 1.2 W/cm^2^, 50% duty cycle) for 6 min, followed by incubation for another 12 h. Finally, the cells were incubated with MTS in RPMI 1640 cell culture medium for 4 h. The absorbance at 490 nm (A) of each well was measured using a SpectraMax M5 microplate. The relative cell viability was calculated as follows: cell viability = (A in treated group/A in control group) × 100%.

### In vitro DC maturation

BMDCs were isolated from the bone marrow of BALB/c mice according to established protocols. Femur bones were isolated from healthy BALB/c mice. Then, both ends of the femur were cut, and the bone marrow was flushed out by slowly injecting RPMI 1640 culture medium. The collected bone marrow was filtered through a 70 μm cell strainer (Falcon^®^) to a 50 mL centrifuge tube, followed by centrifugation at 250 g for 8 min and subsequent lysis of red cells. The obtained cells were resuspended in RPMI 1640 culture medium (10 mL) supplemented with 20 ng/mL GM-CSF and incubated at 37 °C and 5% CO_2_. On day 3, an additional fresh culture medium (10 mL) supplemented with 20 ng/mL GM-CSF was added and incubated for another 3 days. Afterward, BMDCs were harvested by collecting the non-adherent and loosely adherent cells from the suspension.

To study DC maturation, 4T1 cancer cells were first seeded in confocal cell culture dishes at a density of 5 × 10^4^ cells/dish and then cultured overnight for cell attachment. Then the cells were incubated with HNP, PNP, or HPNP ([HP] = 20 μmol/L, [ADA] = 800 U/L, or [aPD-L1] = 6 mg/L) for 12 h. Afterward, the cells were treated with sono-irradiation (1.0 MHz, 1.2 W/cm^2^, 50% duty cycle) for 6 min. After incubation for another 12 h, the cell supernatants of different treatments were collected and added to the BMDCs culture dishes. BMDCs were then cultured for 12 h in the presence or absence of additional Ade (10 mmol/L), followed by staining with anti-CD11c, anti-CD80, and anti-CD86 antibodies for 30 min. After washing three times, the cells were analyzed by Fortessa X20 (BD Biosciences).

### Mouse tumor model implantation

Animal experiments were performed in compliance with Guidelines for Care and Use of Laboratory Animals of the Nanyang Technological University-Institutional Animal Care and Use Committee (NTU-IACUC) and approved by the Institutional Animal Care and Use Committee (IACUC) for Animal Experiment, Singapore. Six-week-old female BALB/c mice and immunodeficient NSG mice were purchased from InVivos (Singapore). Mice were housed in a temperature-controlled (22 °C) room with 12 h dark-light cycles (0700 h on and 1900 h off) and 40–70% humidity. The maximal tumour size/burden of 20 mm was permitted by these ethics committees and the maximal tumour size/burden in this study was not exceeded.

For in vivo fluorescence imaging, ex vivo biodistribution, intratumoral ^1^O_2_ generation, or transcriptome analysis, 4T1 cancer cells suspended in RPMI 1640 cell culture medium were subcutaneously inoculated into the right flank of each mouse (1 × 10^6^ cells/mouse). The mice were then used after 7 days of growth of tumors when the tumor volume reached about 50 mm^3^.

For bilateral tumor model in BALB/c or immunodeficient NSG mice, 4T1 or CT26 cancer cells suspended in RPMI 1640 cell culture medium were subcutaneously inoculated into the right flank (primary tumors) of each mouse (1 × 10^6^ cells/mouse). 5 days later, the same amounts of the cells were subcutaneously inoculated in the left flank (distant tumors) of the same mouse. The mice were used for therapy after 7 days of growth of primary tumors when the primary tumor volume reached about 50 mm^3^.

### Pharmacokinetic analysis

4T1 tumor-bearing BALB/c mice were intravenously injected with 200 μL PBS solutions containing HNP or HPNP ([HP] = 1 mmol/L). Then the blood was collected at 1, 2, 4, 8, 12, and 24 h post-injection of HNP or HPNP. Collected blood samples were stored in an icebox to prevent clotting before centrifugation at 1000 g for 10 min. The concentration of HP was quantified using fluorescence spectra.

### In vivo tumor NIR fluorescence imaging

4T1 tumor-bearing BALB/c mice were randomly divided into three groups (*n* = 3). Mice in each group were intravenously injected with 200 μL PBS solutions containing HNP or HPNP ([HP] = 1 mmol/L). At before (0 h) and different post-injection timepoints, the mice were imaged using an IVIS fluorescence imaging system with the excitation wavelength at 640 nm and the emission wavelength at 700 nm. The fluorescence intensity of the tumor in each mouse was further quantified using Living Image software (*n* = 3).

### Ex vivo biodistribution

At 24 h post-injection of HNP or HPNP ([HP] = 1 mmol/L), 4T1 tumor-bearing mice were euthanized, then hearts, livers, spleens, lungs, kidneys, and the tumors were extracted and imaged using an IVIS fluorescence imaging system with the excitation wavelength at 640 nm and the emission wavelength at 700 nm. Fluorescence intensity quantification of these tissues was further performed using Living Image software (*n* = 3).

### In vivo intratumoral ^1^O_2_ generation

4T1 tumor-bearing mice were randomly divided into three groups (*n* = 3). The mice in each group were intravenously injected with 200 μL PBS solutions containing HNP, PNP, or HPNP ([HP] = 1 mmol/L, [ADA] = 40 U/mL, or [aPD-L1] = 0.3 mg/mL). At 12 h post-injection, the tumor of each mouse was locally injected with SOSG probe (20 μL, 100 μM) and subsequently treated with or without sono-irradiation (1.0 MHz, 1.2 W/cm^2^, 50% duty cycle) for 6 min. After that, the mice were euthanized, and the tumors were collected, fixed with 4% paraformaldehyde, and cut into 10-μm sections. Then the tumor sections were stained with 4’,6-diamidino-2-phenylindole (DAPI), and the fluorescence images of the stained sections were captured using LSM800 confocal laser scanning microscope. The fluorescence intensity of SOSG in each image was quantified using the software ImageJ.

### In vivo sono-metabolic checkpoint trimodal cancer therapy

4T1 tumor-bearing BALB/c mice were randomly divided into seven groups (*n* = 5). The mice in each group were intravenously injected with 200 μL saline or PBS solutions containing mixed proteins (aPD-L1 and ADA), HNP, PNP, or HPNP ([HP] = 1 mmol/L, [ADA] = 40 U/mL, or [aPD-L1] = 0.3 mg/mL). At 12 h post-injection, the primary tumor of each mouse was treated with or without sono-irradiation (1.0 MHz, 1.2 W/cm^2^, 50% duty cycle) for 6 min. Then the sizes of primary and distant tumors and the body weights of mice were measured every 2 days for 22 days. The tumor volume was calculated as follows: volume = (tumor length) × (tumor width)^2^/2. After 14 days post-injection, the mice in each group were euthanized, and the tumor tissues were collected for subsequent H&E and immunofluorescence staining (caspase 3 and granzyme B). For bilateral CT26 tumor model, the mice were randomly divided into three groups (*n* = 5). The mice in each group were intravenously injected with 200 μL saline or PBS solutions containing HNP or HPNP ([HP] = 1 mmol/L, [ADA] = 40 U/mL, or [aPD-L1] = 0.3 mg/mL). Then the sono-metabolic checkpoint trimodal cancer therapy and corresponding experiments were conducted as similar as that for bilateral 4T1 tumor model.

In addition, 4T1 tumor-bearing NSG mice were randomly divided into three groups (*n* = 5). The mice in each group were intravenously injected with 200 μL saline or PBS solutions containing HPNP ([HP] = 1 mmol/L). At 12 h post-injection, the primary tumor of each mouse was treated with or without sono-irradiation (1.0 MHz, 1.2 W/cm^2^, 50% duty cycle) for 6 min. Then the sizes of primary and distant tumors and the body weights of mice were measured every 2 days for 14 days. The tumor volume was calculated as follows: volume = (tumor length) × (tumor width)^2^/2.

### In vivo tumor rechallenge study

4T1 cancer cells suspended in RPMI 1640 cell culture medium were subcutaneously inoculated into the right flank of each mouse (1 × 10^6^ cells/mouse). 4T1 tumor-bearing BALB/c mice were randomly divided into 2 groups (*n* = 5). After 7, 9, 11, and 13 days, the mice in each group were intravenously injected with 200 μL PBS solutions containing HNP or HPNP ([HP] = 1 mmol/L). At 12 h post-injection, the tumor of each mouse was treated with sono-irradiation (1.0 MHz, 1.2 W/cm^2^, 50% duty cycle) for 6 min. After 40 days, 4T1 cancer cells suspended in RPMI 1640 cell culture medium were subcutaneously inoculated into the left flank of each mouse (1 × 10^6^ cells/mouse). Then the sizes of the rechallenged tumors and the body weights of mice were measured every 2 days for 32 days. The tumor volume was calculated as follows: volume = (tumor length) × (tumor width)^2^/2.

### Histological studies

After 14 days of different treatments, 4T1 or CT26 tumor-bearing BALB/c mice in each group (*n* = 3) were euthanized, and the major organs (heart, liver, spleen, and kidney) were collected and fixed with 4% paraformaldehyde for H&E staining to evaluate the biosafety of NPs. After 22 days of different treatments, 4T1 tumor-bearing BALB/c mice in each group (*n* = 3) were euthanized, and the lung tissues were collected and fixed with 4% paraformaldehyde for H&E staining to evaluate tumor metastasis. The H&E-stained tissue sections were examined on a Nikon ECLIPSE 80i microscope (Nikon Instruments).

### In vivo evaluation of ICD, DC maturation, and cytokine release

After 7 days of different treatments, the mice in each group were euthanized. The tumor tissues were collected for subsequent immunofluorescence staining (HMGB1). And the TDLNs were collected and directly ground. Then the obtained single cells were incubated with the anti-CD16 antibody for 10 min and then stained with Zombie UV™ dye, anti-CD11c, anti-CD80, and anti-CD86 antibodies for another 30 min. After washing three times, the cells were analyzed by Fortessa X20 (BD Biosciences).

After 5, 7, or 14 days of different treatments, the mice in each group were euthanized, and the blood samples were collected for the detection of TNF-α and IL-6 by ELISA.

### In vivo evaluation of immune effector and suppressor cells

After 14 days of different treatments, the mice in each group were euthanized, and the tumor tissues, blood, and spleens were collected. For tumor tissues, 1 mg/mL type I collagenase, 100 μg/mL type IV collagenase, and 100 μg/ml DNase I were added and incubated for 2 h at 37 °C, then the tissues were filtered through a 70 μm nylon cell strainer. Then the red blood cells of the obtained tumor single-cell suspension were removed using ACK lysis, and the cells were washed thrice with cell staining buffer for subsequent staining of various antibodies. For blood, the red blood cells were first removed using ACK lysis, and the obtained cells were washed thrice with cell staining buffer for subsequent staining of various antibodies. For the spleen, the tissues were directly ground and washed thrice with cell staining buffer for subsequent staining of various antibodies.

After that, the obtained single-cell suspensions were incubated with the anti-CD16 antibody for 10 min and subsequent Zombie UV™ dye for 30 min. For the cell surface antigen staining, the cells were stained with anti-CD45, anti-CD3, anti-CD4, anti-CD8a, anti-CD69, anti-CD11b, anti-F4/80, anti-CD206, and anti-Gr-1 antibodies for another 30 min. For the intracellular antigen staining, the cells were fixed and permeabilized, then stained with anti-Foxp3 antibodies for another 30 min. After washing thrice with cell staining buffer, the cells were analyzed by Fortessa X20 (BD Biosciences).

### In vivo evaluation of Ade and cAMP contents

After 14 days of different treatments, the mice in each group were euthanized, and the tumor tissues were collected to detect the Ade and cAMP content. Then the tumor tissues were homogenized using a homogenizer. Then the supernatant was collected to quantify the Ade and cAMP contents via HPLC analysis and ELISA, respectively.

### Transcriptome analysis

4T1 tumor-bearing BALB/c mice were randomly divided into three groups (*n* = 3). The mice in each group were intravenously injected with 200 μL saline or PBS solutions containing HPNP ([HP] = 1 mmol/L, [ADA] = 40 U/mL, or [aPD-L1] = 0.3 mg/mL). At 12 h post-injection, the primary tumor of each mouse was treated with or without sono-irradiation (1.0 MHz, 1.2 W/cm^2^, 50% duty cycle) for 6 min. After 14 days of different treatments, 4T1 tumor-bearing BALB/c mice in each group (*n* = 3) were euthanized, and the tumor tissues were collected. The high-throughput sequencing and data analysis were performed in NovogeneAIT Genomics Singapore Pte Ltd.

### Statistical analysis

The data in all experiments were expressed as mean ± SD. Statistical calculation of experimental data was performed using the one-way ANOVA with a Tukey posthoc test or two-tailed Student’s *t*-test. Kaplan–Meier analysis was used to plot survival curves, and the significance of differences was assessed using the log-rank test. For all tests, *p* values less than 0.05 were considered statistically significant; *p < 0.05, **p < 0.01, ***p < 0.001, and ****p < 0.0001. All statistical calculations were performed using GraphPad Prism 8.0.

### Reporting summary

Further information on research design is available in the Nature Research Reporting Summary linked to this article.

## Supplementary information

Supplementary Information

Reporting Summary

## Data Availability

The transcriptomic data are available at NCBI under Project PRJNA778004. The remaining data are available within the Article, Supplementary Information or Source Data file. Source data are provided with this paper.

## Source data

Source Data
