# Supplementary material for: Catalytical nano-immunocomplexes for remote-controlled sono-metabolic checkpoint trimodal cancer therapy
Source: Nat Commun. 2022 Jun 16;13:3468. doi: 10.1038/s41467-022-31044-6 (PMC9203767; doi:10.1038/s41467-022-31044-6)
Supplement: Supplementary file 1 — Supplementary Information [file 41467_2022_31044_MOESM1_ESM.docx]

Supplementary Information for

**Catalytical Nano-Immunocomplexes for Remote-Controlled Sono-Metabolic Checkpoint Trimodal Cancer Therapy**

*Chi Zhang^1^, Jingsheng Huang^1^, Ziling Zeng^1^, Shasha He^1^, Penghui Cheng^1^, Jingchao Li^1^, and Kanyi Pu^1,2,3^**

^1^ School of Chemical and Biomedical Engineering, Nanyang Technological University, 70 Nanyang Drive, 637457, Singapore.

^2^ Division of Chemistry and Biological Chemistry, School of Physical and Mathematical Sciences, Nanyang Technological University, 21 Nanyang Link, 637371, Singapore.

^3^ Lee Kong Chian School of Medicine, Nanyang Technological University, 59 Nanyang Drive, 636921, Singapore

*E-mail: [kypu@ntu.edu.sg](mailto:kypu@ntu.edu.sg)


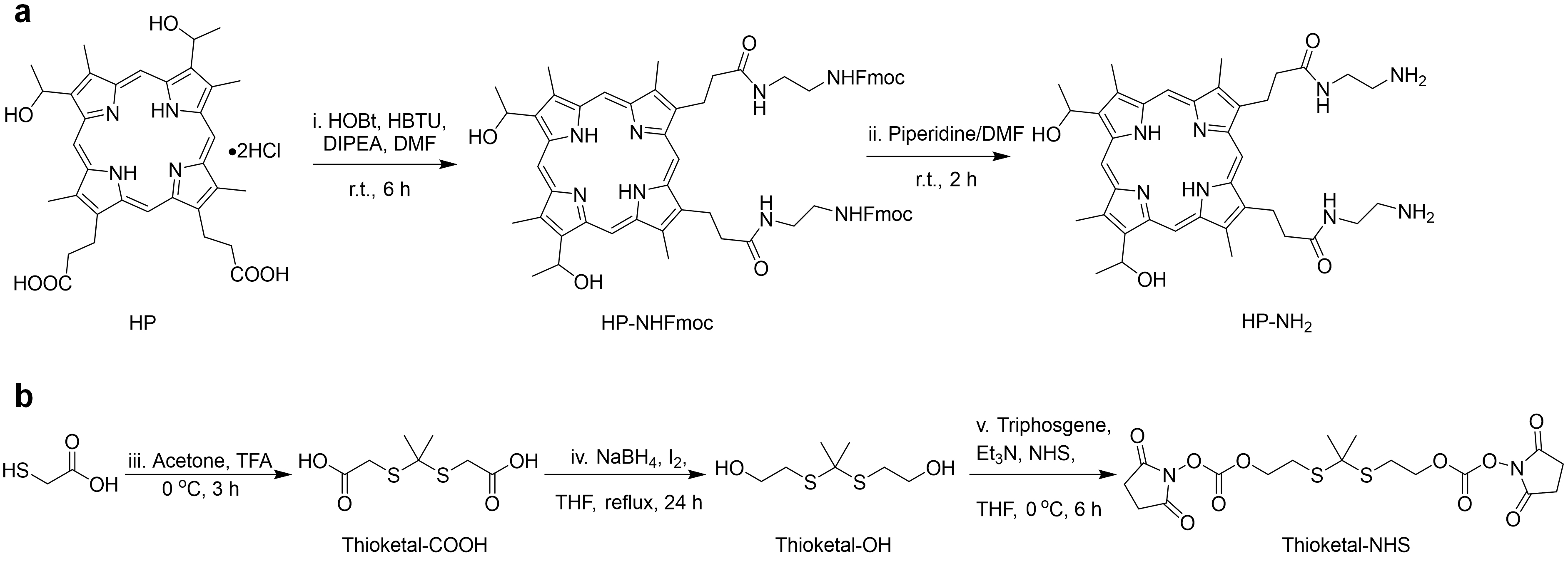


**Supplementary Figure 1.** Synthetic routes of the ethylenediamine-modified hematoporphyrin (HP-NH_2_) and the crosslinker thioketal-NHS. **a** Synthesis of HP-NH_2_: i) HOBT, HBTU, DIPEA, DMF, r.t., 6 h; ii) piperidine/DMF (20%, V/V), 2 h. **b** Synthesis of thioketal-NHS: iii) acetone, TFA, 3 h; iv) NaBH_4_, I_2_, THF, reflux, 24 h; v) triphosgene, Et_3_N, NHS, THF, 0 ^o^C, 6 h.


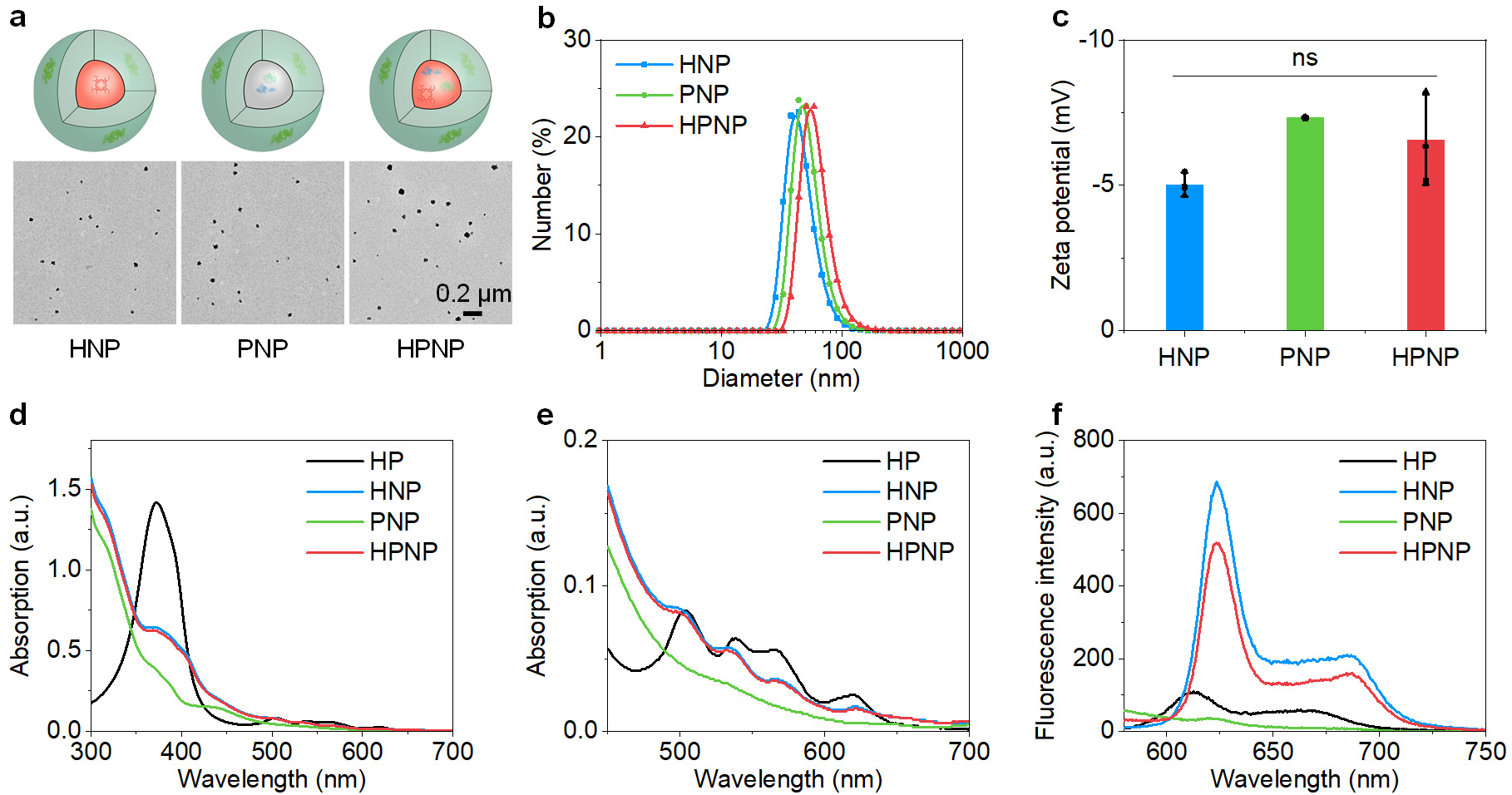


**Supplementary Figure 2.** Characterization of HNP, PNP, and HPNP. **a** The schematic illustration and TEM images of HNP, PNP, and HPNP. The experiments were repeated independently three times with similar results. **b** DLS profiles of HNP, PNP, and HPNP in 1× PBS buffer (pH 7.4). **c** Zeta potential of HNP, PNP, and HPNP in 1× PBS buffer (pH 7.4) (*n*=3 independent experiments). HNP versus PNP versus HPNP: not significant (ns). **d** UV/Vis absorption spectra of HNP, PNP, and HPNP in 1× PBS buffer (pH 7.4). **e** UV/Vis absorption spectra of HNP, PNP, and HPNP at an amplified wavelength range from 450 nm to 700 nm. **f** Fluorescence spectra of HNP, PNP, and HPNP in 1× PBS buffer (pH 7.4) with an excitation wavelength at 410 nm. Statistical significance in **c** was calculated via one-way ANOVA with a Tukey post-hoc test. The mean values and SD are presented.


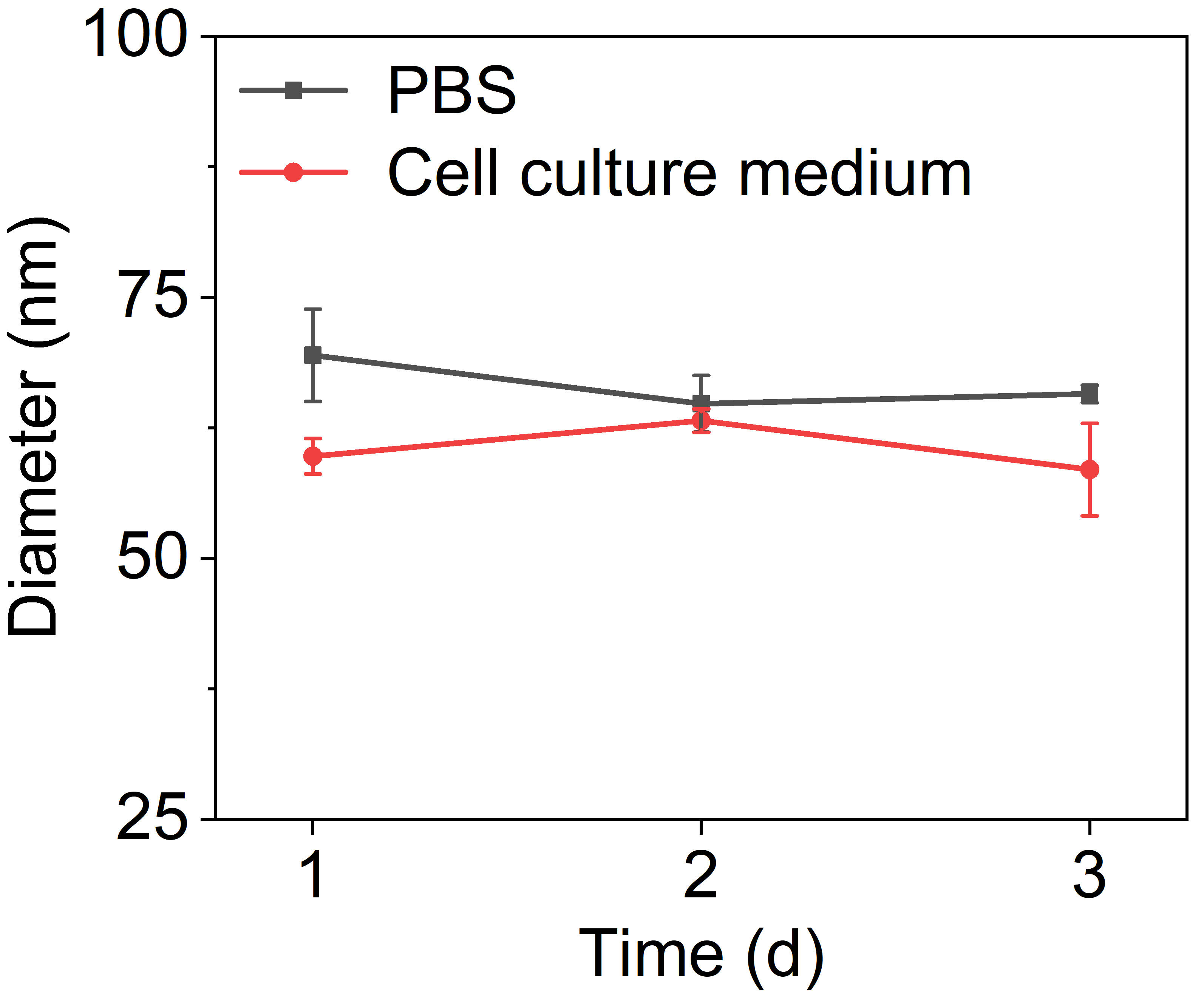


**Supplementary Figure 3.** DLS profiles of HPNP in 1× PBS buffer (pH 7.4) and RPMI 1640 cell culture medium for 3 days (*n*=3 independent experiments). The mean values and SD are presented.


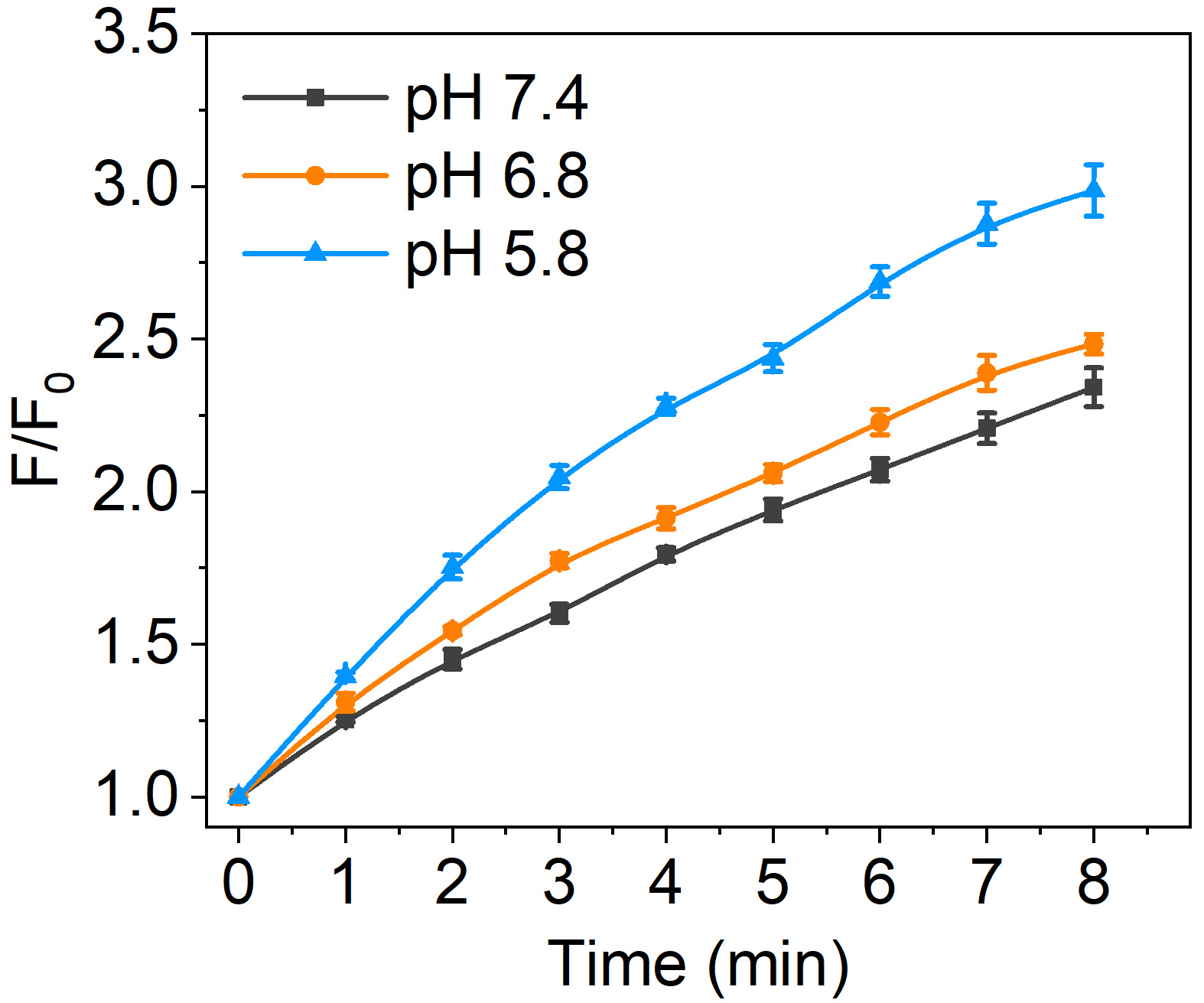


**Supplementary Figure 4.** The generation of ^1^O_2_ in HNP, PNP, and HPNP in 1× PBS buffer (pH 7.4) ([HP]=20 μmol/L or [ADA]=800 U/L) as a function of the sono-irradiation (1.0 MHz, 1.2 W/cm^2^, 50% duty cycle) time (*n*=3 independent experiments). HPNP versus PBS: p < 0.0001. The mean values and SD are presented.


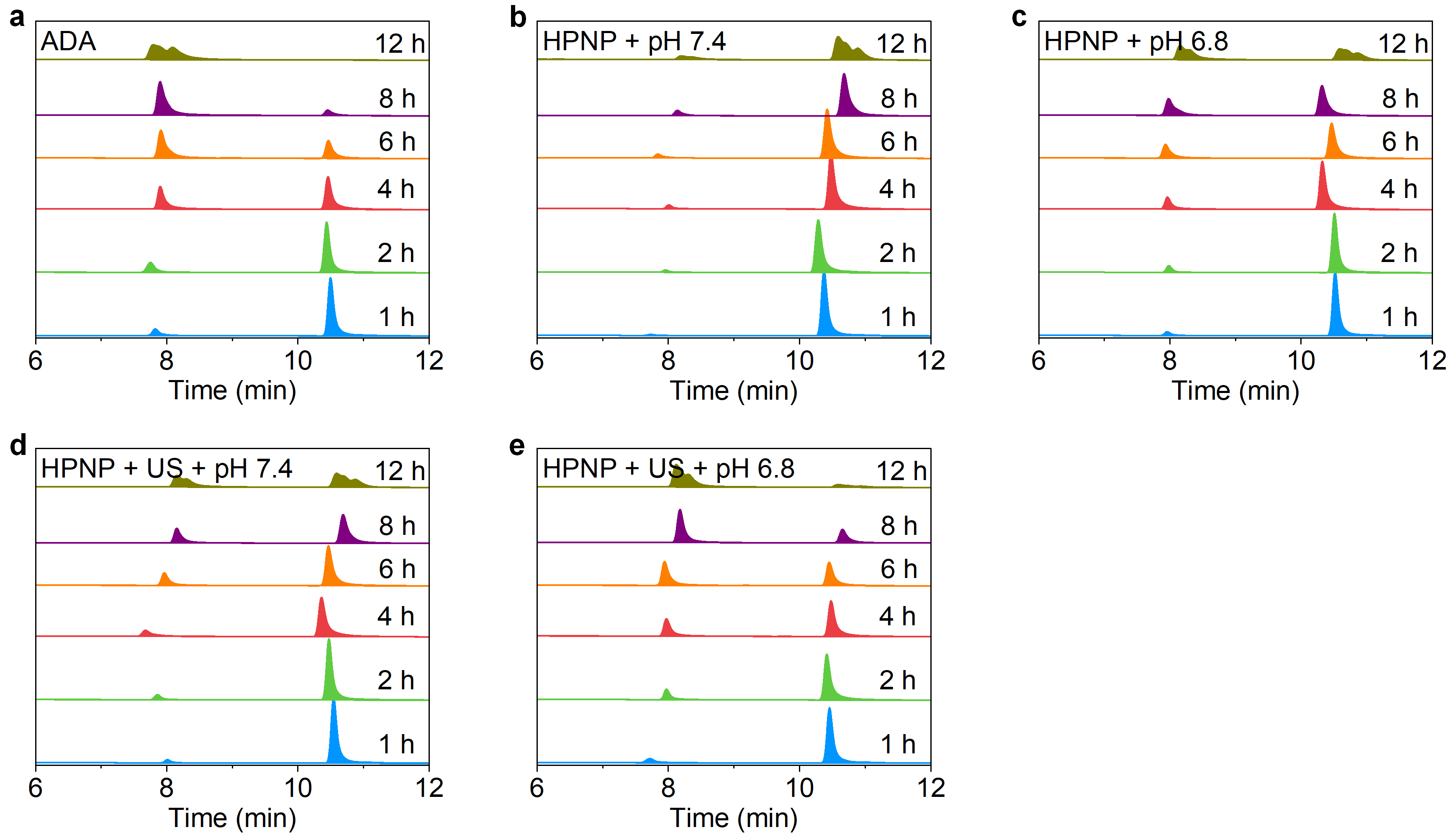


**Supplementary Figure 5.** HPLC profiles of Ade and Ino after incubation of (**a**) ADA or HPNPs in PBS solutions containing Ade with different treatments, including (**b**) pH 7.4, (**c**) pH 7.4 with sono-irradiation, (**d**) pH 6.8, and (**e**) pH 6.8 with sono-irradiation for various times (1, 2, 4, 6, 8, and 12 h).


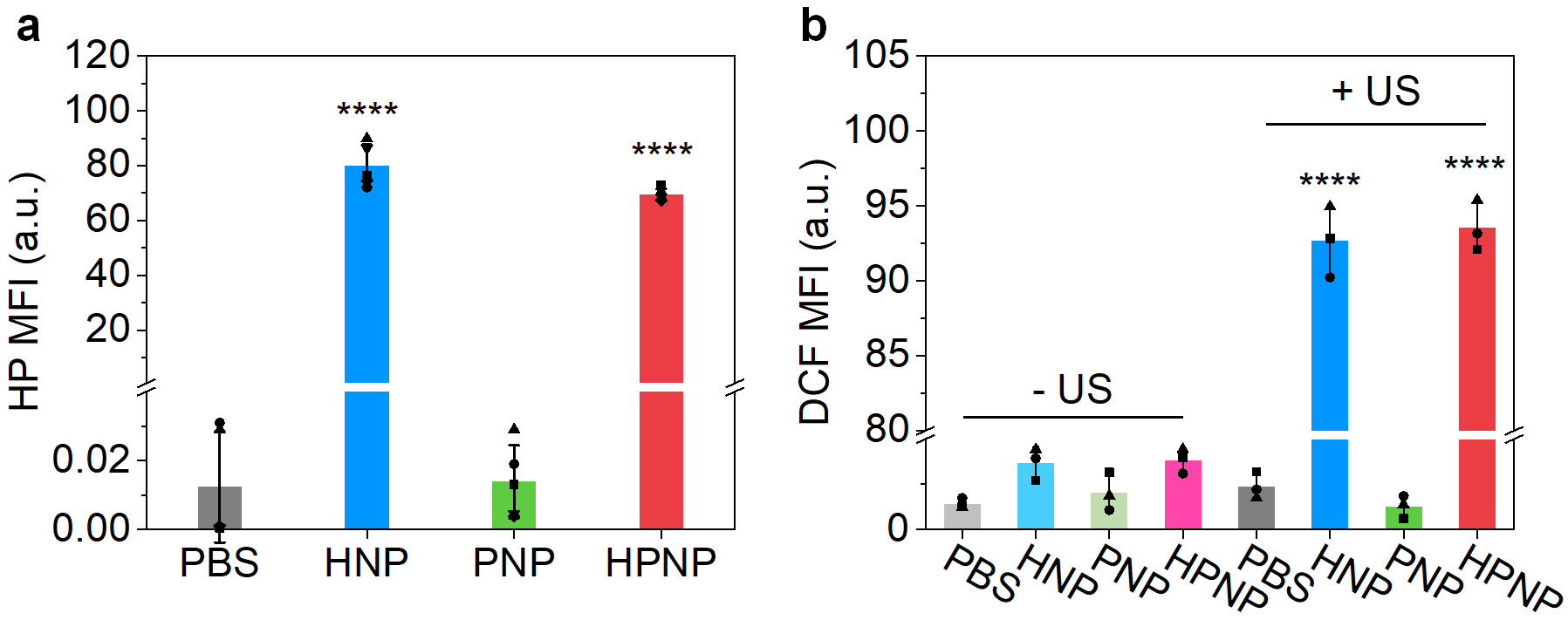


**Supplementary Figure 6.** **a** Quantitative analysis of HP MFIs in HNP-, PNP-, or HPNP-incubated 4T1 cancer cells after 12 h incubation ([HP]=20 μmol/L or [ADA]=800 U/L) (*n*=5 independent images). HPNP versus PBS: *p* < 0.0001; HNP versus PBS: *p* < 0.0001. **b** Quantitative analysis of DCF MFIs in HNP-, PNP-, or HPNP-incubated 4T1 cancer cells after 12 h incubation with or without sono-irradiation ([HP]=20 μmol/L or [ADA]=800 U/L) (*n*=3 independent images). HPNP + US versus HPNP: *p* < 0.0001; HNP + US versus HNP: *p* < 0.0001. Statistical significance was calculated via one-way ANOVA with a Tukey post-hoc test. *****p* < 0.0001. The mean values and SD are presented.


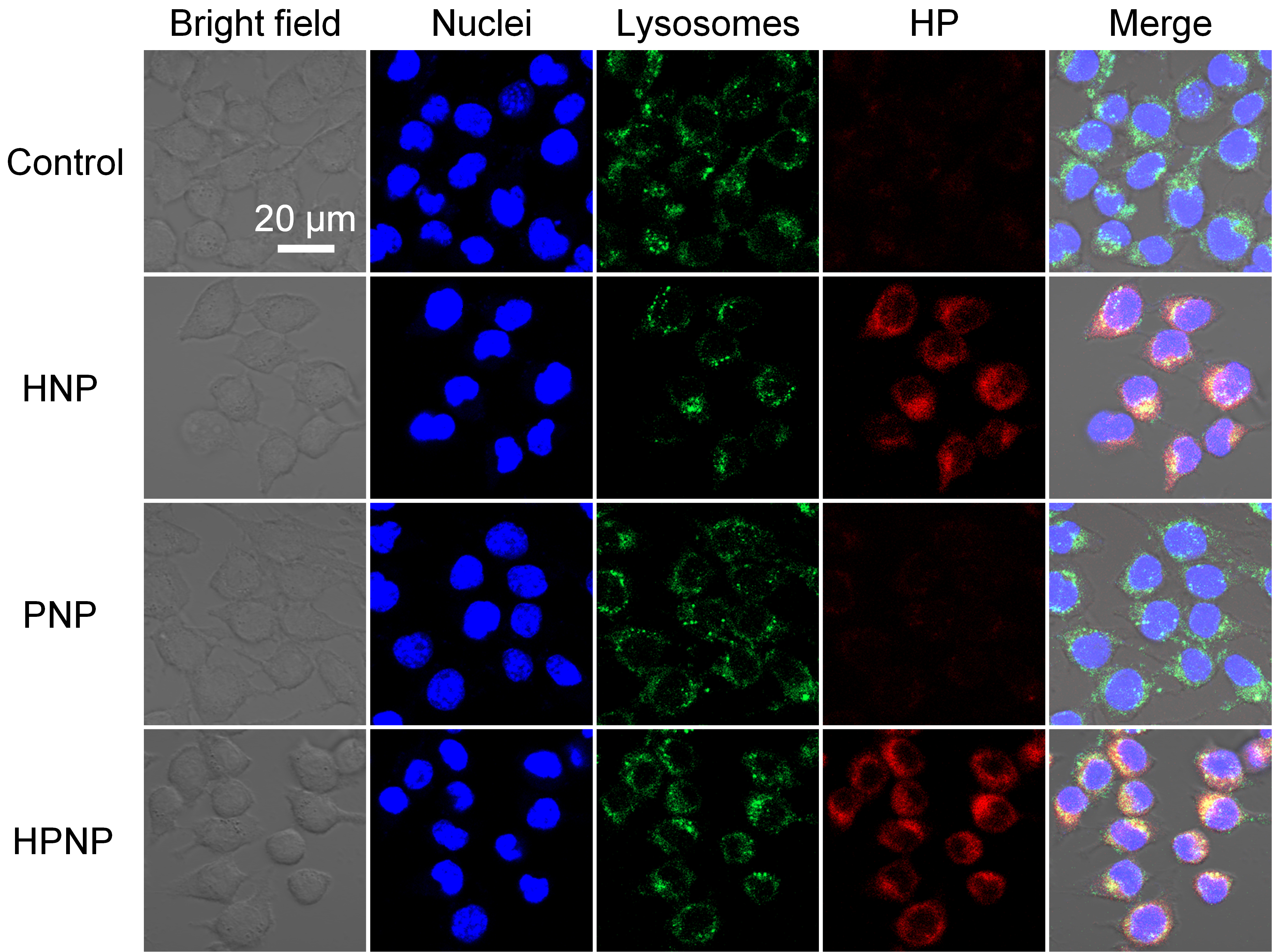


**Supplementary Figure 7.** Confocal fluorescence images of 4T1 cells after 6 h incubation with HNP, PNP, or HPNP ([HP]=20 μmol/L or [ADA]=800 U/L), followed by staining with lysosome tracker (Green DND-26) and cell nuclei dye (Hoechst 33342). The experiments were repeated independently three times with similar results.


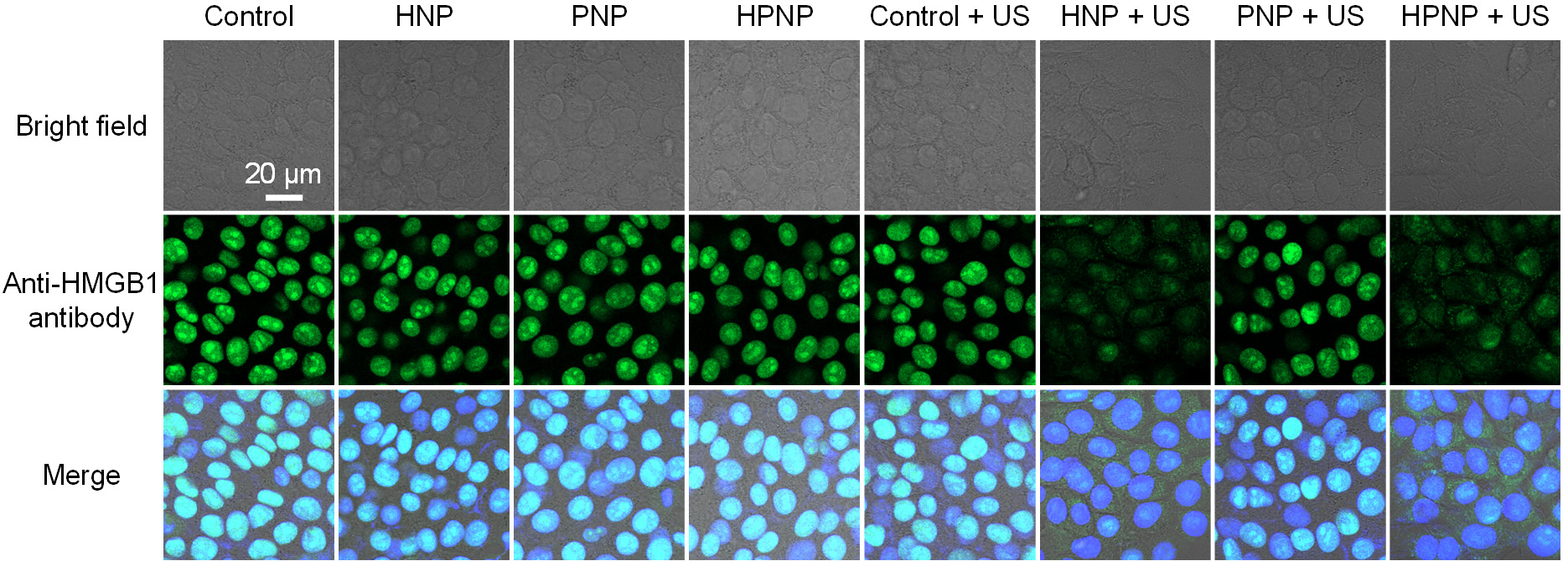


**Supplementary Figure 8.** Confocal fluorescence images of HNP-, PNP-, or HPNP-4T1 cells ([HP]=20 μmol/L or [ADA]=800 U/L) after 12 h incubation with or without sono-irradiation (1.0 MHz, 1.2 W/cm^2^, 50% duty cycle) for 6 min, followed by staining with FITC-labelled anti-HMGB1 antibodies and cell nuclei dye (Hoechst 33342). The experiments were repeated independently three times with similar results.


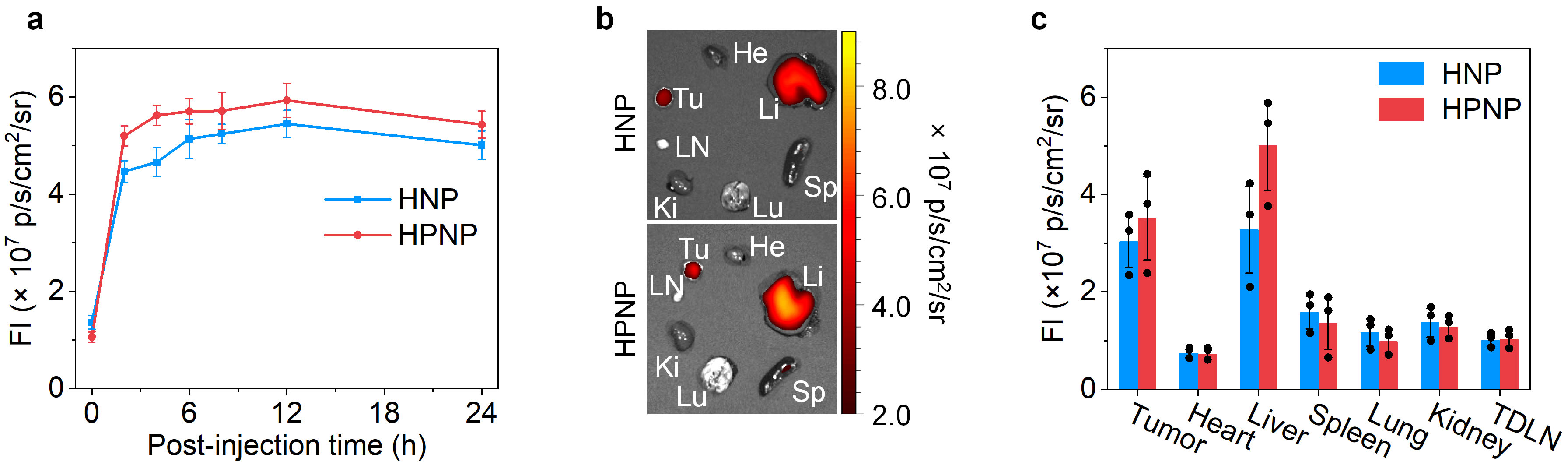


**Supplementary Figure 9.** **a** Quantitative NIR fluorescence intensity (FI) of tumor tissues in 4T1 tumor-bearing mice at t=0, 2, 4, 6, 8, 12, or 24 h after the intravenous injection of HNP or HPNP (200 μL, [HP]=1 mmol/L, or [ADA]=40 U/mL) (*n*=3). Ex vivo NIR fluorescence images (**b**) and quantitative NIR FI (**c**) of tumors and major organs in 4T1 tumor-bearing mice at 24 h after intravenous injection of HNP or HPNP (200 μL, [HP]=1 mmol/L, or [ADA]=40 U/mL) (*n*=3). The mean values and SD are presented.


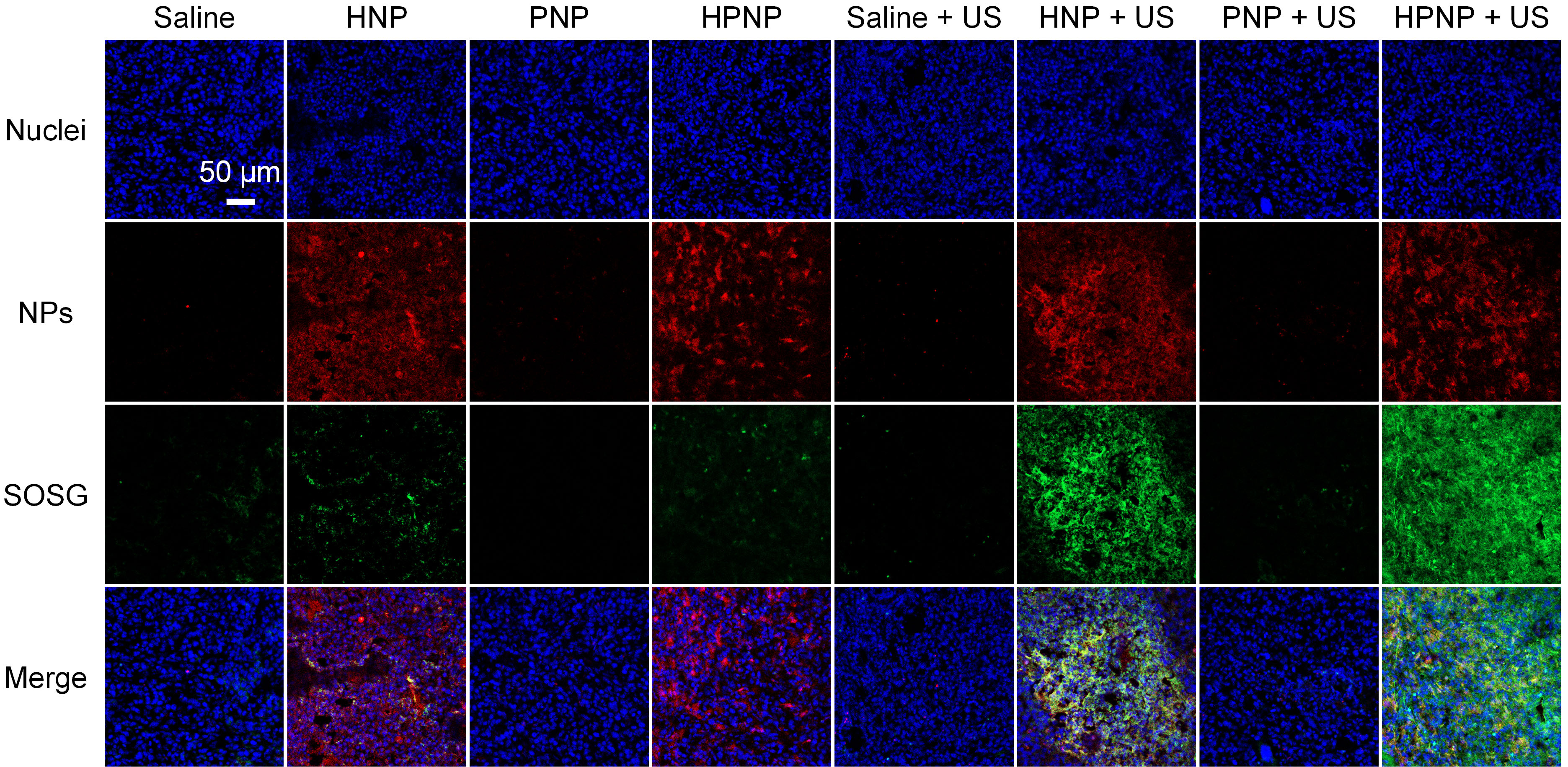


**Supplementary Figure 10.** Confocal fluorescence images of tumor tissues from saline-, HNP-, PNP-, or HPNP-injected (200 μL, [HP]=1 mmol/L, or [ADA]=40 U/mL) 4T1 tumor-bearing mice with or without sono-irradiation (1.0 MHz, 1.2 W/cm^2^, 50% duty cycle) for 6 min. Blue fluorescence showed the cell nucleus stained with DAPI, red fluorescence showed the signals from HP, and green fluorescence showed the signals from SOSG. Images are representative of three biologically independent mice.


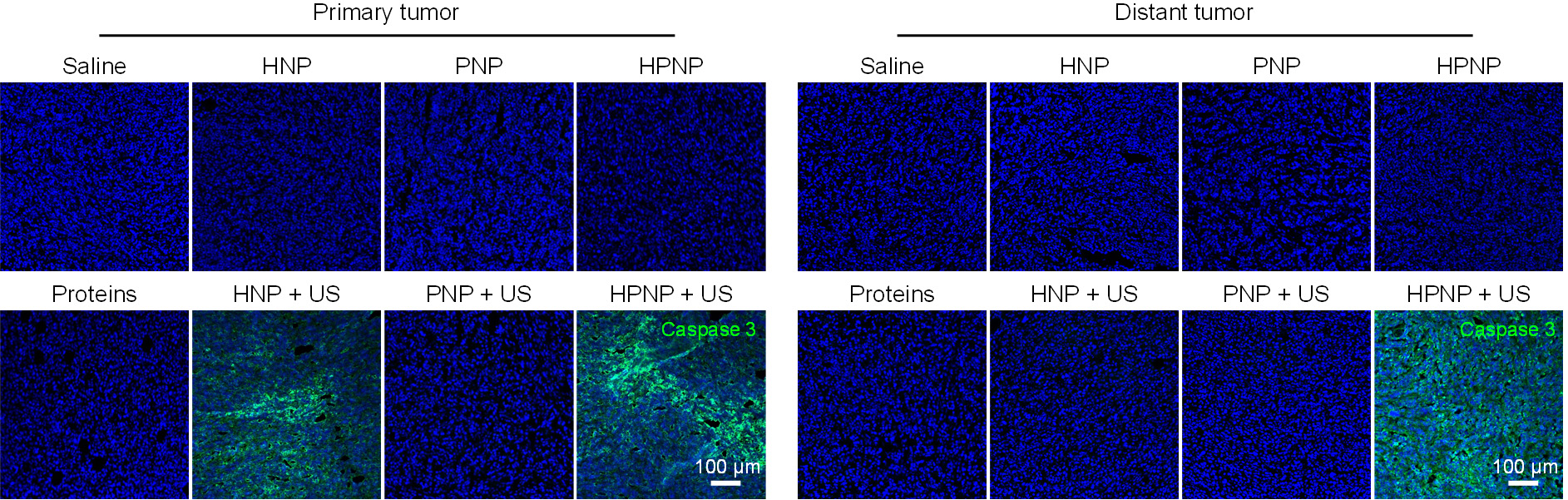


**Supplementary Figure 11.** Immunofluorescence staining images of caspase-3 in primary and distant tumor tissues of 4T1 tumor-bearing mice after different treatments. The cell nucleus stained with DAPI and caspase-3 stained with antibodies showed blue and green fluorescence signals, respectively. Images are representative of three biologically independent mice.

**
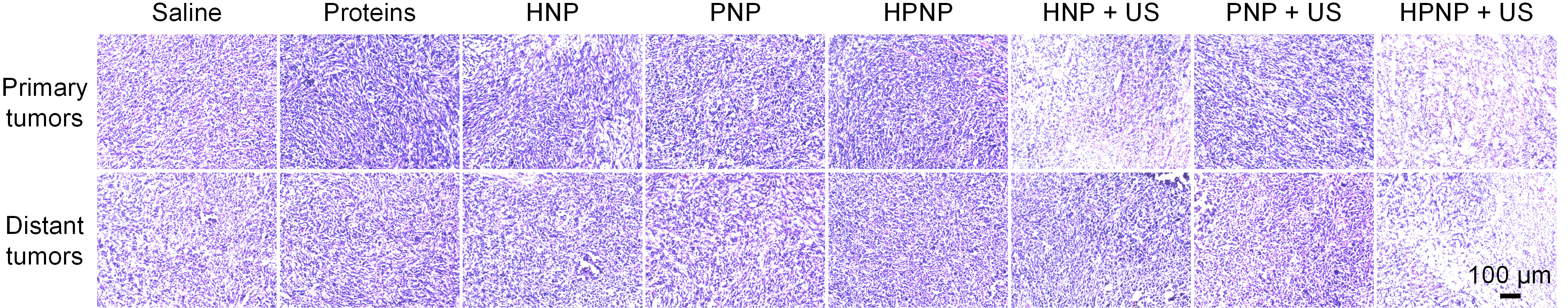
**

**Supplementary Figure 12.** Histological H&E staining of primary and distant tumors in 4T1 tumor-bearing mice after different treatments. Images are representative of three biologically independent mice.

**
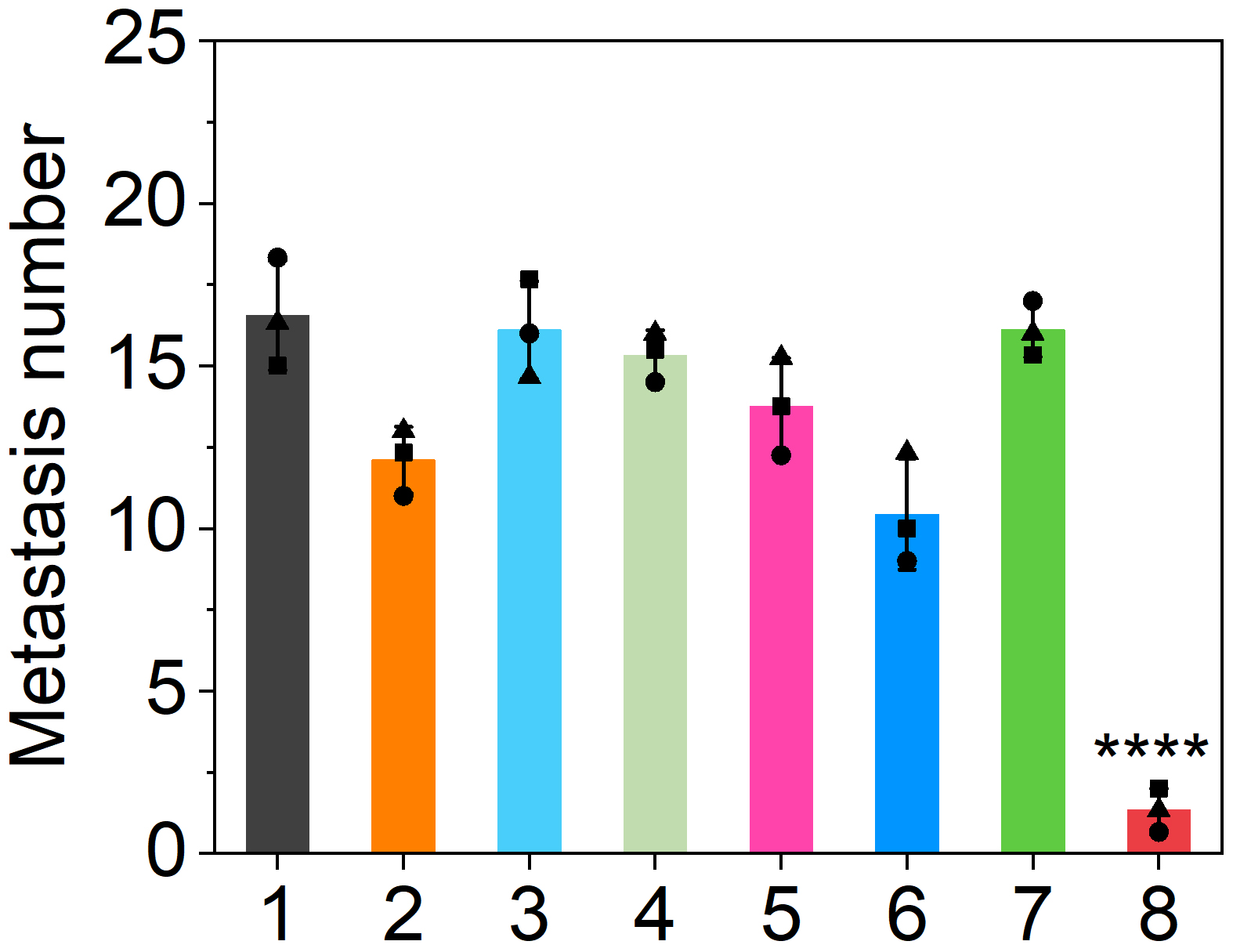
**

**Supplementary Figure 13.** Number of metastatic nodules in the histological images of the whole lung tissues from 4T1 tumor-bearing mice after different treatments (*n*=3). 8 versus other groups: *p* < 0.0001. Statistical significance was calculated via one-way ANOVA with a Tukey post-hoc test. *****p* < 0.0001. The mean values and SD are presented.

**
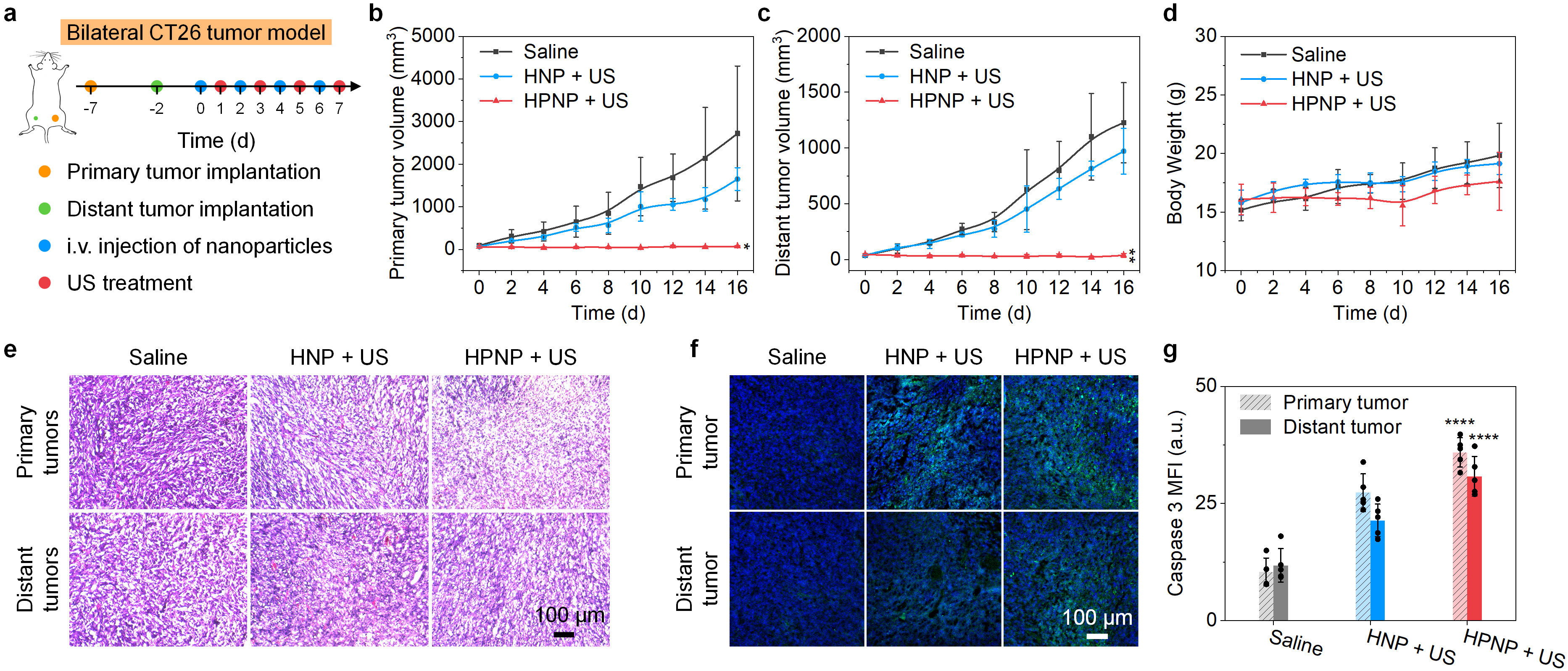
**

**Supplementary Figure 14.** In vivo NIR fluorescence imaging and nano-immunocomplex-mediated activatable sono-metabolic checkpoint trimodal cancer therapy in CT26 tumor-bearing mice. **a** Schematic illustration of the schedule for bilateral CT26 tumor model implantation and nano-immunocomplex-mediated activatable sono-metabolic checkpoint trimodal cancer therapy. Growth curves of primary tumors (**b**) and distant tumors (**c**) in CT26 tumor-bearing mice after different treatments (injection dose: 200 μL, [HP]=1 mmol/L; sono-irradiation: 1.0 MHz, 1.2 W/cm^2^, 50% duty cycle for 6 min; *n*=5). HPNP + US versus Saline in **b**: *p* = 0.0441; HPNP + US versus Saline in **c**: *p* = 0.0069. **d** Body weights of CT26 tumor-bearing mice after different treatments. **e** Histological H&E staining of primary and distant tumors in CT26 tumor-bearing mice after different treatments. Immunofluorescence staining images (**f**) and quantification (**g**) of caspase-3 expression in primary and distant tumor tissues of CT26 tumor-bearing mice after different treatments (*n*=5). The cell nucleus stained with DAPI and caspase-3 stained with antibodies showed blue and green fluorescence signals, respectively. HPNP + US versus Saline in primary tumors: *p* < 0.0001; HPNP + US versus Saline in distant tumors: *p* < 0.0001. Statistical significance in **b** and **c** was calculated via two-tailed Student’s t-test. Statistical significance in **g** was calculated via one-way ANOVA with a Tukey post-hoc test. **p* < 0.05; ***p* < 0.01; *****p* < 0.0001. The mean values and SD are presented.

**
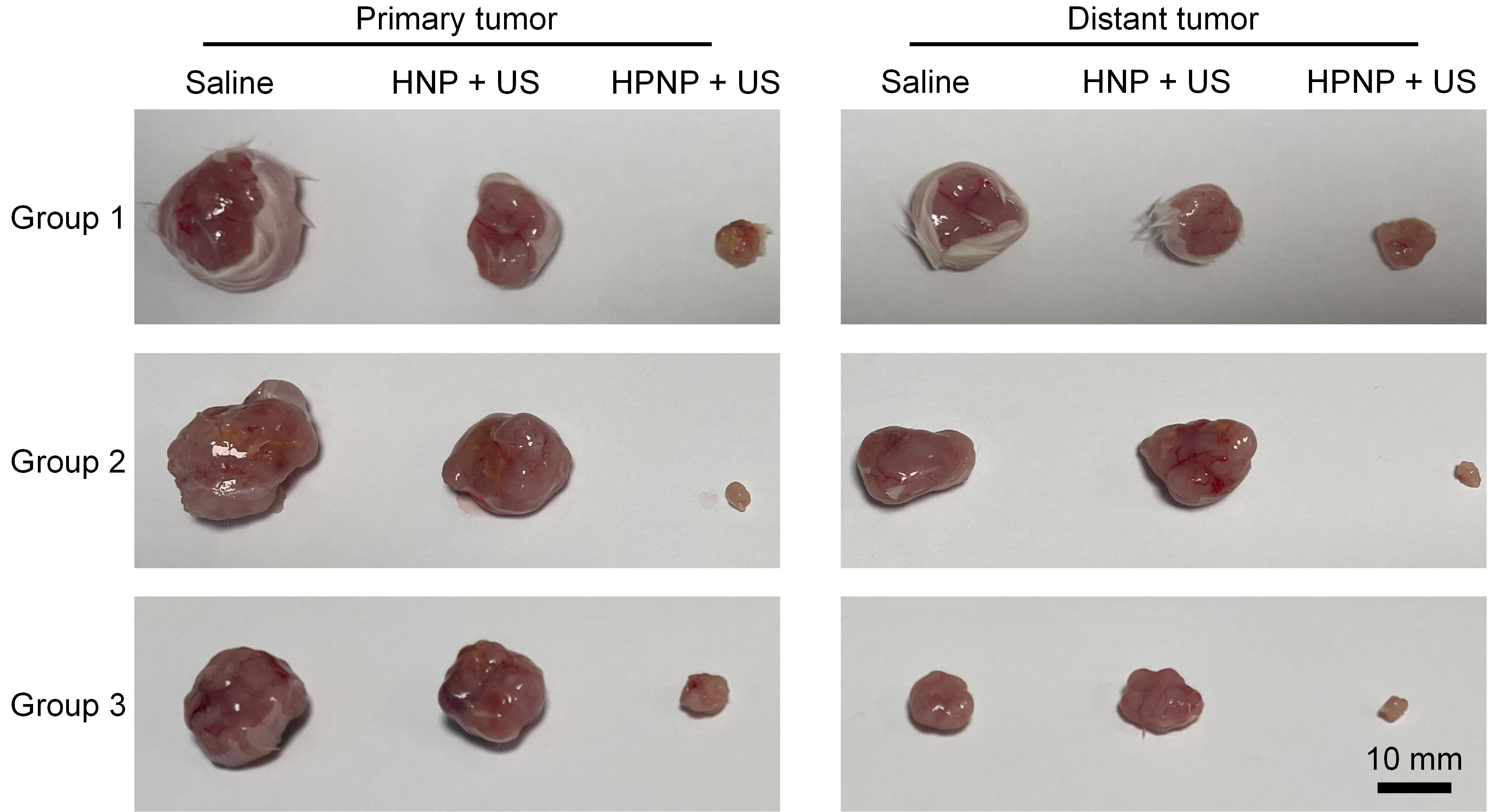
**

**Supplementary Figure 15.** Representative tumor images from CT26 tumor-bearing mice at 16 days post-injection of saline, HNP, or HPNP (200 μL, [HP]=1 mmol/L, or [ADA]=40 U/mL) with sono-irradiation (1.0 MHz, 1.2 W/cm^2^, 50% duty cycle) for 6 min.

**
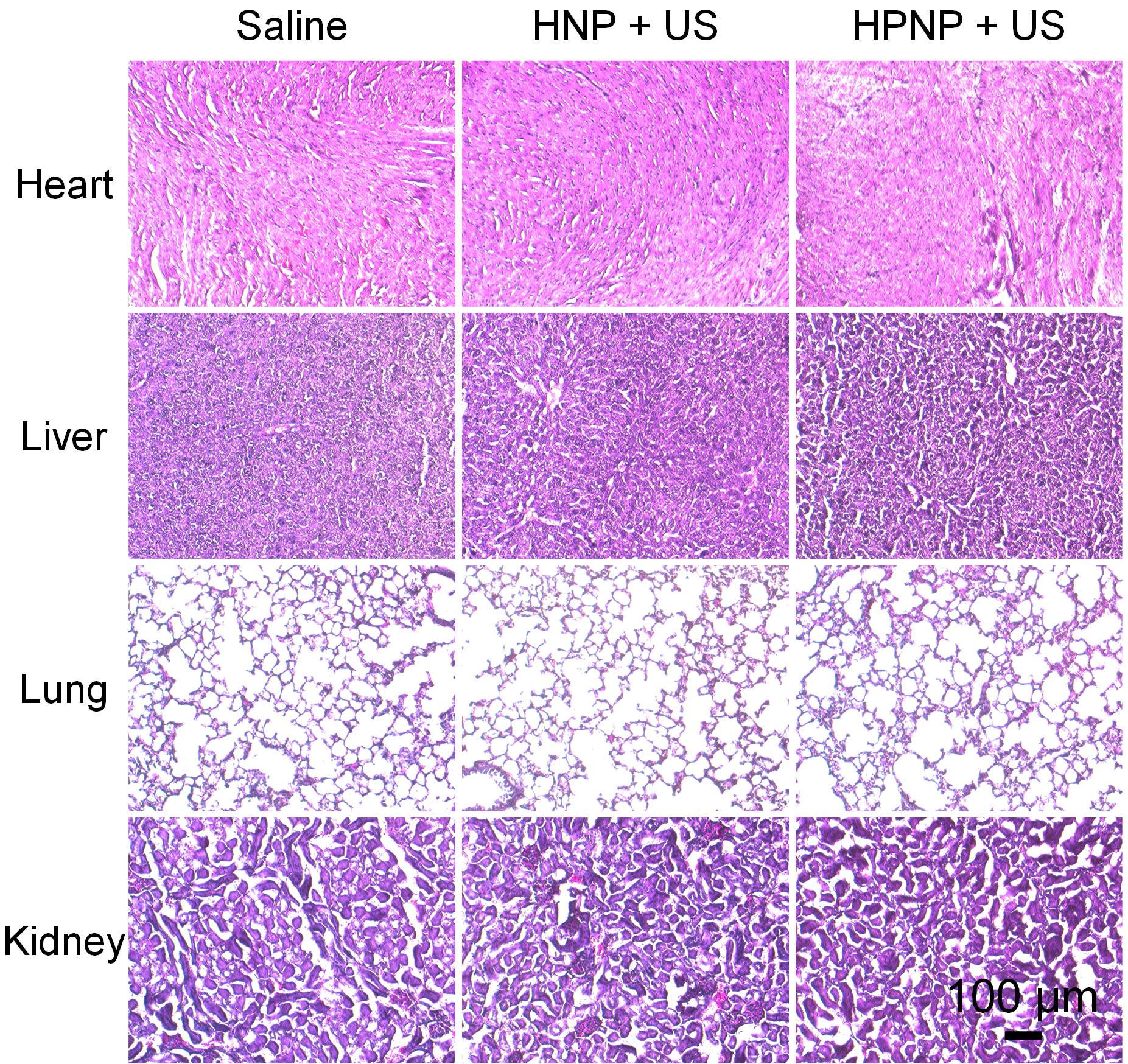
**

**Supplementary Figure 16.** Histological H&E staining of the major organs (heart, liver, lung, and kidney) from CT26 tumor-bearing mice at day 16 after intravenous injection of saline, HNP, or HPNP (200 μL, [HP]=1 mmol/L, or [ADA]=40 U/mL) with sono-irradiation (1.0 MHz, 1.2 W/cm^2^, 50% duty cycle) for 6 min. Images are representative of three biologically independent mice.

**
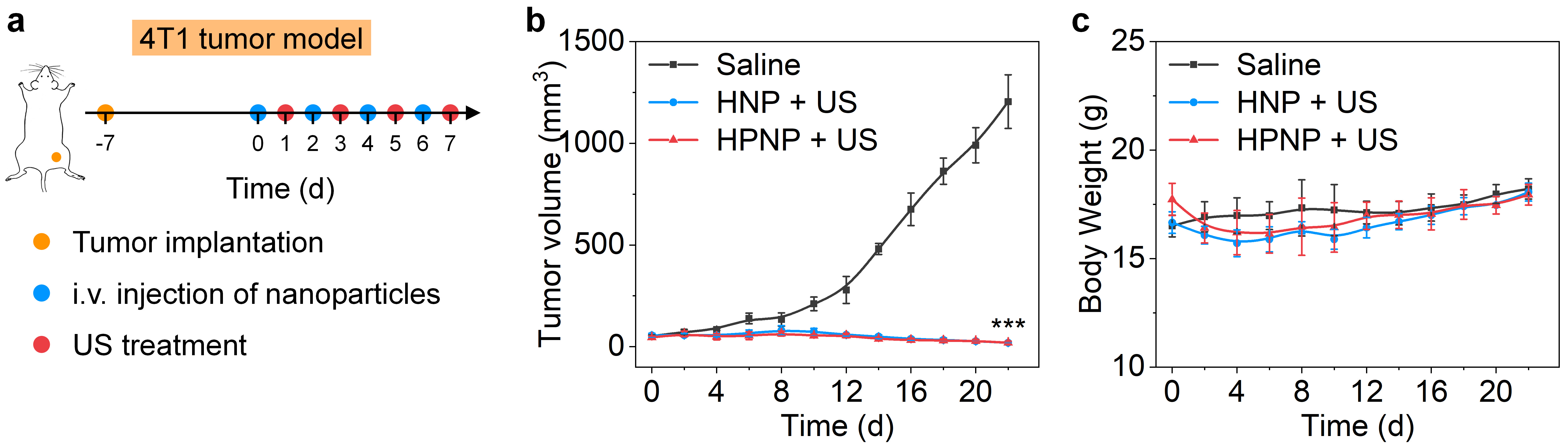
**

**Supplementary Figure 17.** In vivo nano-immunocomplex-mediated activatable sono-metabolic checkpoint trimodal cancer therapy in 4T1 tumor-bearing mice. **a** Schematic illustration of the schedule for unilateral tumor model implantation and nano-immunocomplex-mediated activatable sono-metabolic checkpoint trimodal cancer therapy. **b** Growth curves of original tumors in 4T1 tumor-bearing mice after different treatments (injection dose: 200 μL, [HP]=1 mmol/L; sono-irradiation: 1.0 MHz, 1.2 W/cm^2^, 50% duty cycle for 6 min; *n*=5). HPNP + US versus Saline: *p* = 0.0006; HNP + US versus Saline: *p* = 0.0006. **c** Body weights of 4T1 tumor-bearing mice after different treatments (*n*=5). Statistical significance in **b** was calculated via two-tailed Student’s t-test. ****p* < 0.001. The mean values and SD are presented.


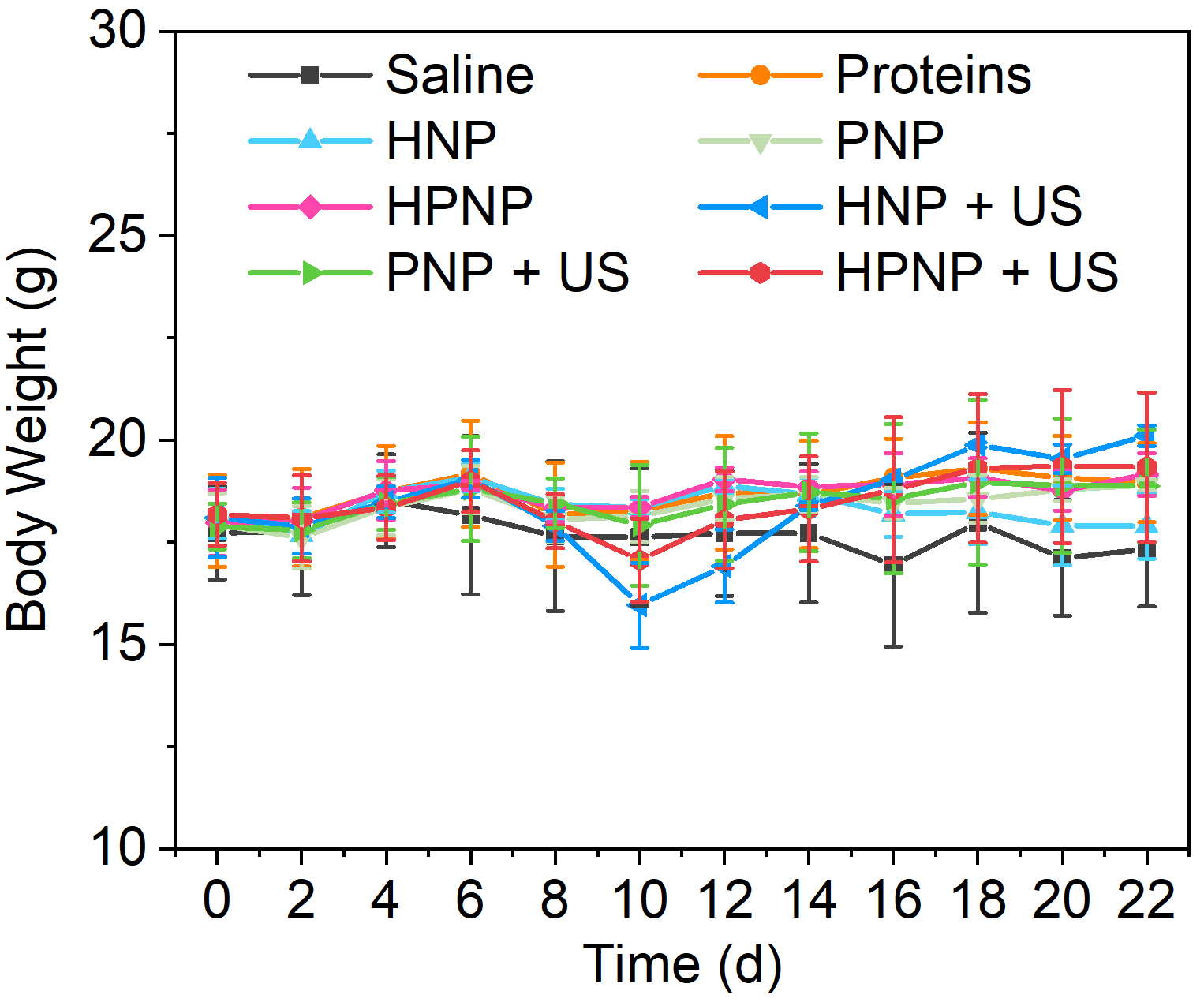


**Supplementary Figure 18.** Body weights of 4T1 tumor-bearing mice after different treatments (*n*=5, refer to Figure 4). The mean values and SD are presented.

**
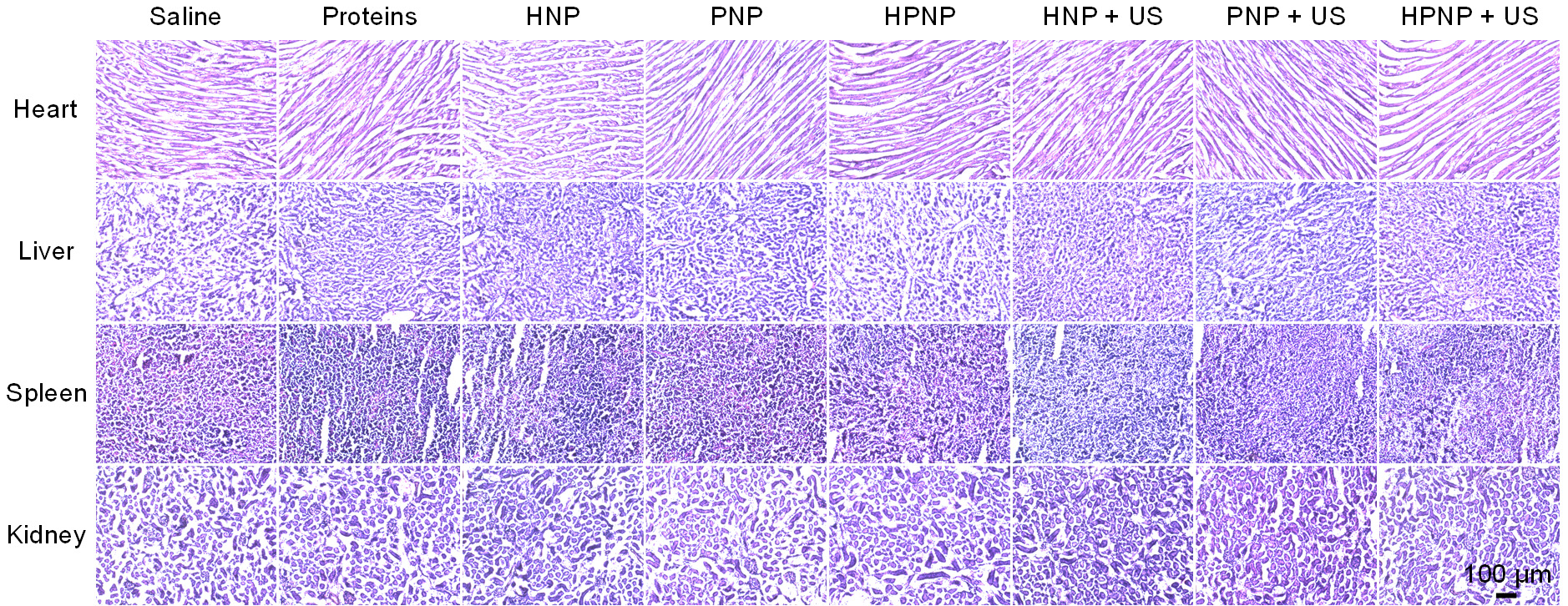
**

**Supplementary Figure 19.** Histological H&E staining of the major organs (heart, liver, spleen, and kidney) from 4T1 tumor-bearing mice at day 14 after intravenous injection of saline, proteins, HNP, PNP, or HPNP (200 μL, [HP]=1 mmol/L, or [ADA]=40 U/mL) with or without sono-irradiation (1.0 MHz, 1.2 W/cm^2^, 50% duty cycle) for 6 min. Images are representative of three biologically independent mice.

**
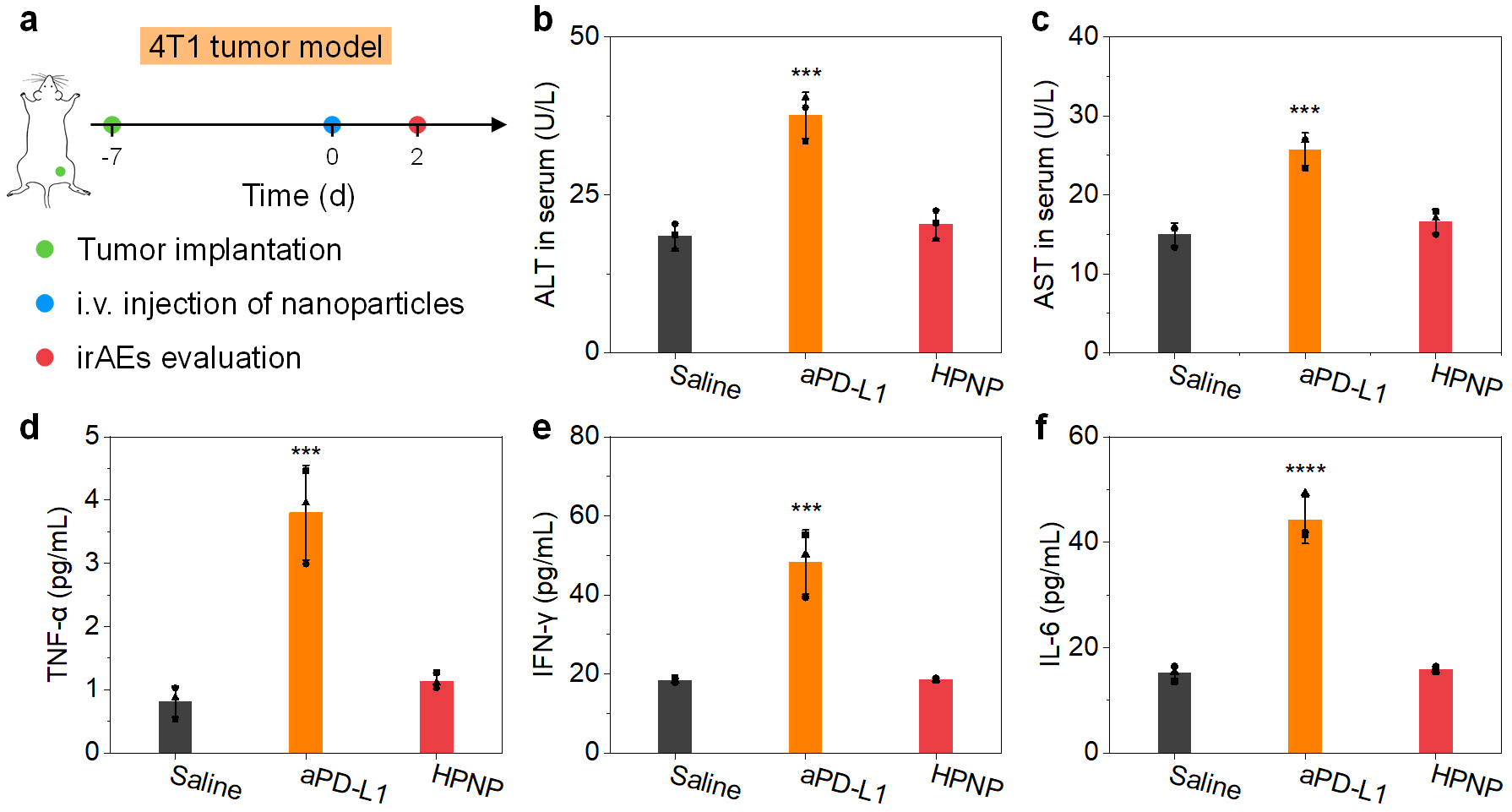
**

**Supplementary Figure 20.** In vivo immune-related adverse events (irAEs) study on 4T1 tumor-bearing mice. **a** Schematic illustration of the schedule for 4T1 tumor implantation and irAEs evaluation. Liver function index levels including ALT (**b**) and AST (**c**) concentrations in serum from mice after different treatments (*n*=3). aPD-L1 versus Saline in **b**: *p* = 0.0004; aPD-L1 versus Saline in **c**: *p* = 0.0006. In vivo cytokine detection of TNF-α (**d**), IFN-γ (**e**), and IL-6 (**f**) in serum from mice after different treatments (*n*=3). aPD-L1 versus Saline in **d**: *p* = 0.0005; aPD-L1 versus Saline in **e**: *p* = 0.0006; aPD-L1 versus Saline in **f**: *p* < 0.0001. Statistical significance was calculated via one-way ANOVA with a Tukey post-hoc test. ****p* < 0.001; *****p* < 0.0001. The mean values and SD are presented.

**
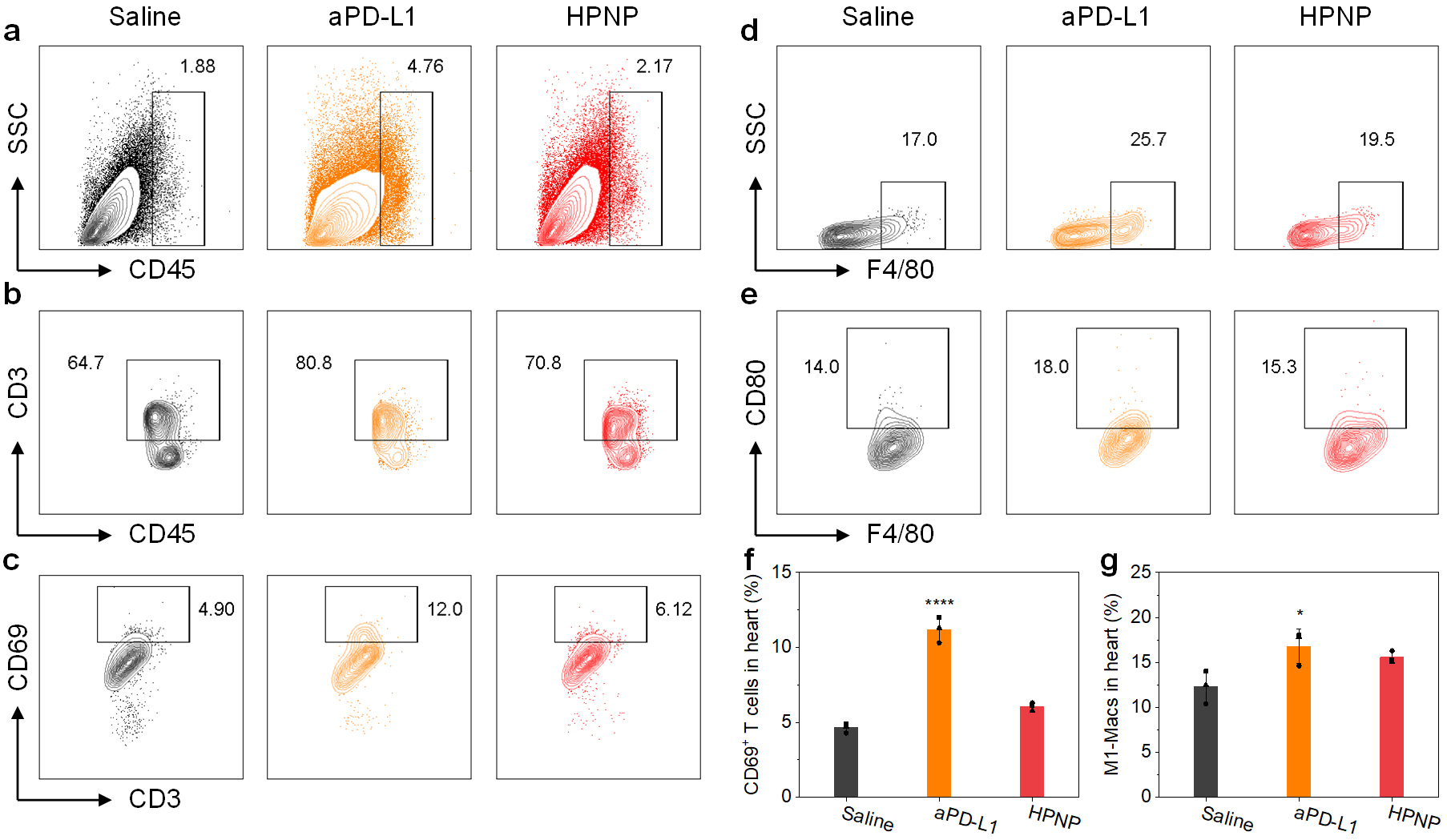
**

**Supplementary Figure 21.** Immune cell infiltrating study in heart tissues after different treatments. Flow cytometry assay of heart-infiltrating CD45^+^ leukocytes (**a**), CD45^+^CD3^+^ T cells (**b**), CD3^+^CD69^+^ activated T cells (**c**), F4/80^+^ macrophages (**d**), and F4/80^+^CD80^+^ M1-Macs (**e**) in 4T1 tumor-bearing mice after different treatments (*n*=3). Quantification of CD3^+^CD69^+^ activated T cells (**f**) and F4/80^+^CD80^+^ M1-Macs (**g**) in heart tissues from 4T1 tumor-bearing mice after different treatments (*n*=3). aPD-L1 versus Saline in **f**: *p* < 0.0001; aPD-L1 versus Saline in **g**: *p* = 0.0029. Statistical significance was calculated via one-way ANOVA with a Tukey post-hoc test. **p* < 0.05; *****p* < 0.0001. The mean values and SD are presented.

**
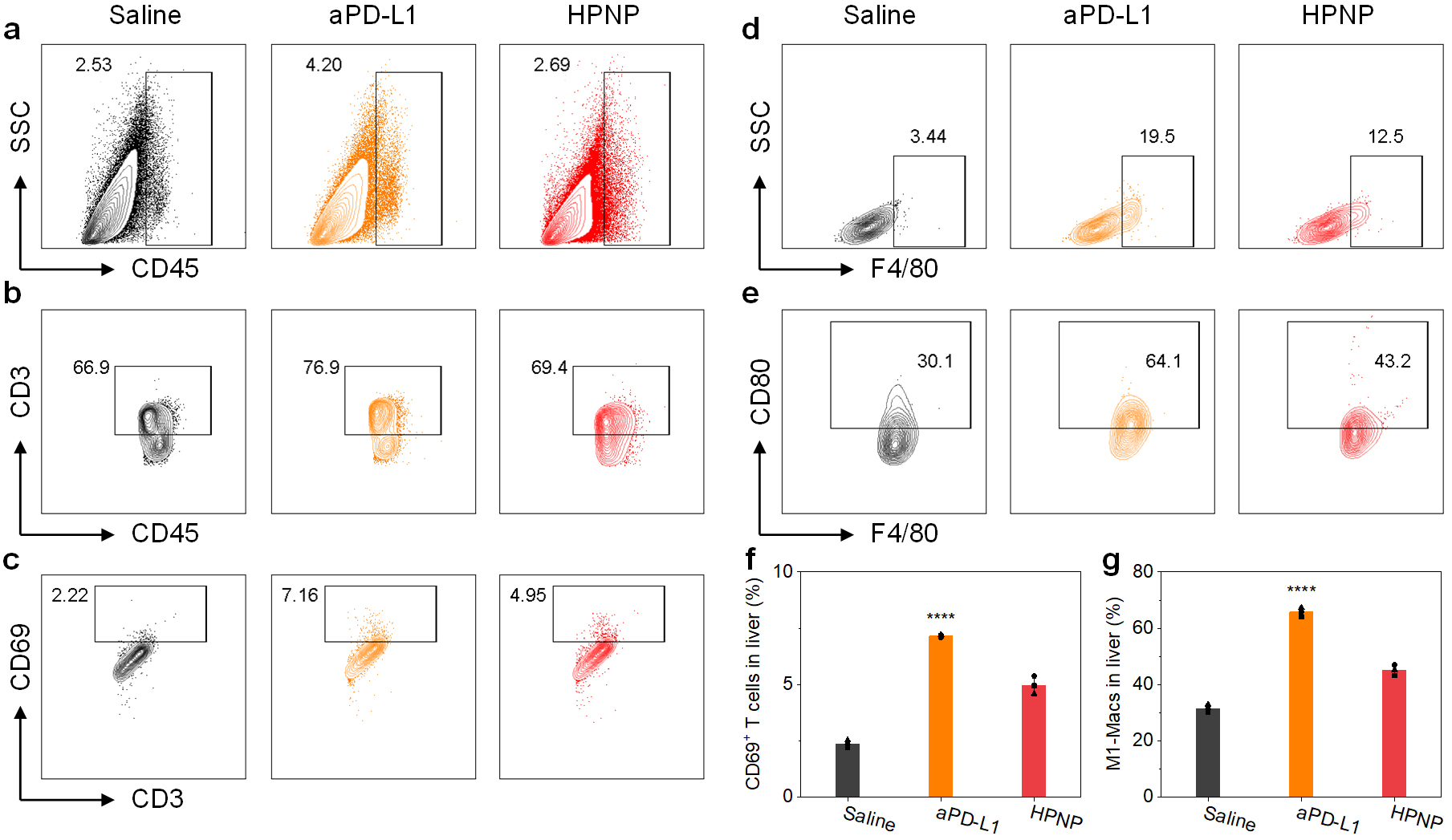
**

**Supplementary Figure 22.** Immune cell infiltrating study in liver tissues after different treatments. Flow cytometry assay of liver-infiltrating CD45^+^ leukocytes (**a**), CD45^+^CD3^+^ T cells (**b**), CD3^+^CD69^+^ activated T cells (**c**), F4/80^+^ macrophages (**d**), and F4/80^+^CD80^+^ M1-Macs (**e**) in 4T1 tumor-bearing mice after different treatments (*n*=3). Quantification of CD3^+^CD69^+^ activated T cells (**f**) and F4/80^+^CD80^+^ M1-Macs (**g**) in liver tissues from 4T1 tumor-bearing mice after different treatments (*n*=3). aPD-L1 versus Saline in **f**: *p* < 0.0001; aPD-L1 versus Saline in **g**: *p* < 0.0001. Statistical significance was calculated via one-way ANOVA with a Tukey post-hoc test. *****p* < 0.0001. The mean values and SD are presented.

**
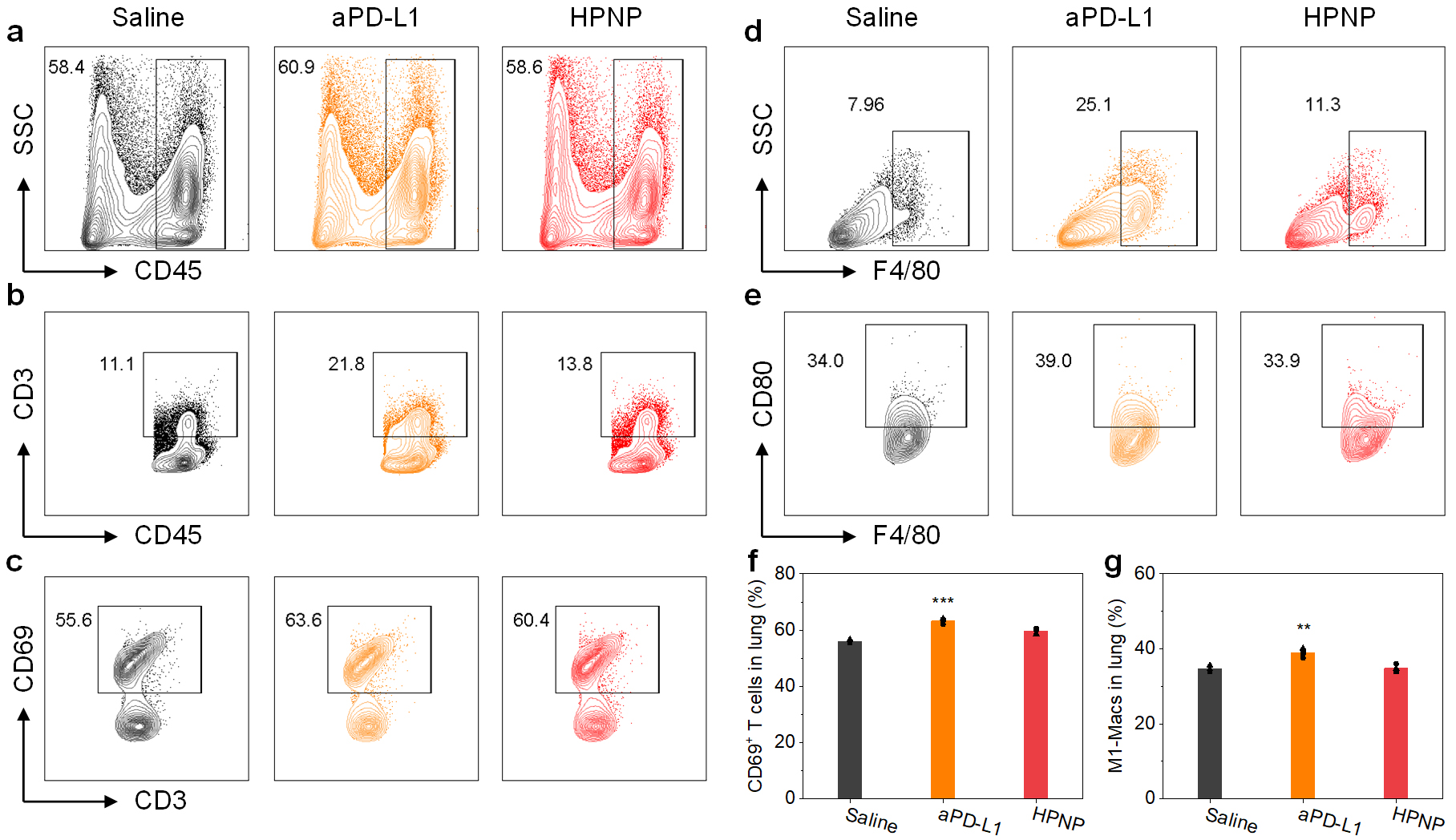
**

**Supplementary Figure 23.** Immune cell infiltrating study in lung tissues after different treatments. Flow cytometry assay of lung-infiltrating CD45^+^ leukocytes (**a**), CD45^+^CD3^+^ T cells (**b**), CD3^+^CD69^+^ activated T cells (**c**), F4/80^+^ macrophages (**d**), and F4/80^+^CD80^+^ M1-Macs (**e**) in 4T1 tumor-bearing mice after different treatments (*n*=3). Quantification of CD3^+^CD69^+^ activated T cells (**f**) and F4/80^+^CD80^+^ M1-Macs (**g**) in lung tissues from 4T1 tumor-bearing mice after different treatments (*n*=3). aPD-L1 versus Saline in **f**: *p* = 0.0001; aPD-L1 versus Saline in **g**: *p* = 0.0065. Statistical significance was calculated via one-way ANOVA with a Tukey post-hoc test. ***p* < 0.01; ****p* < 0.001. The mean values and SD are presented.

**
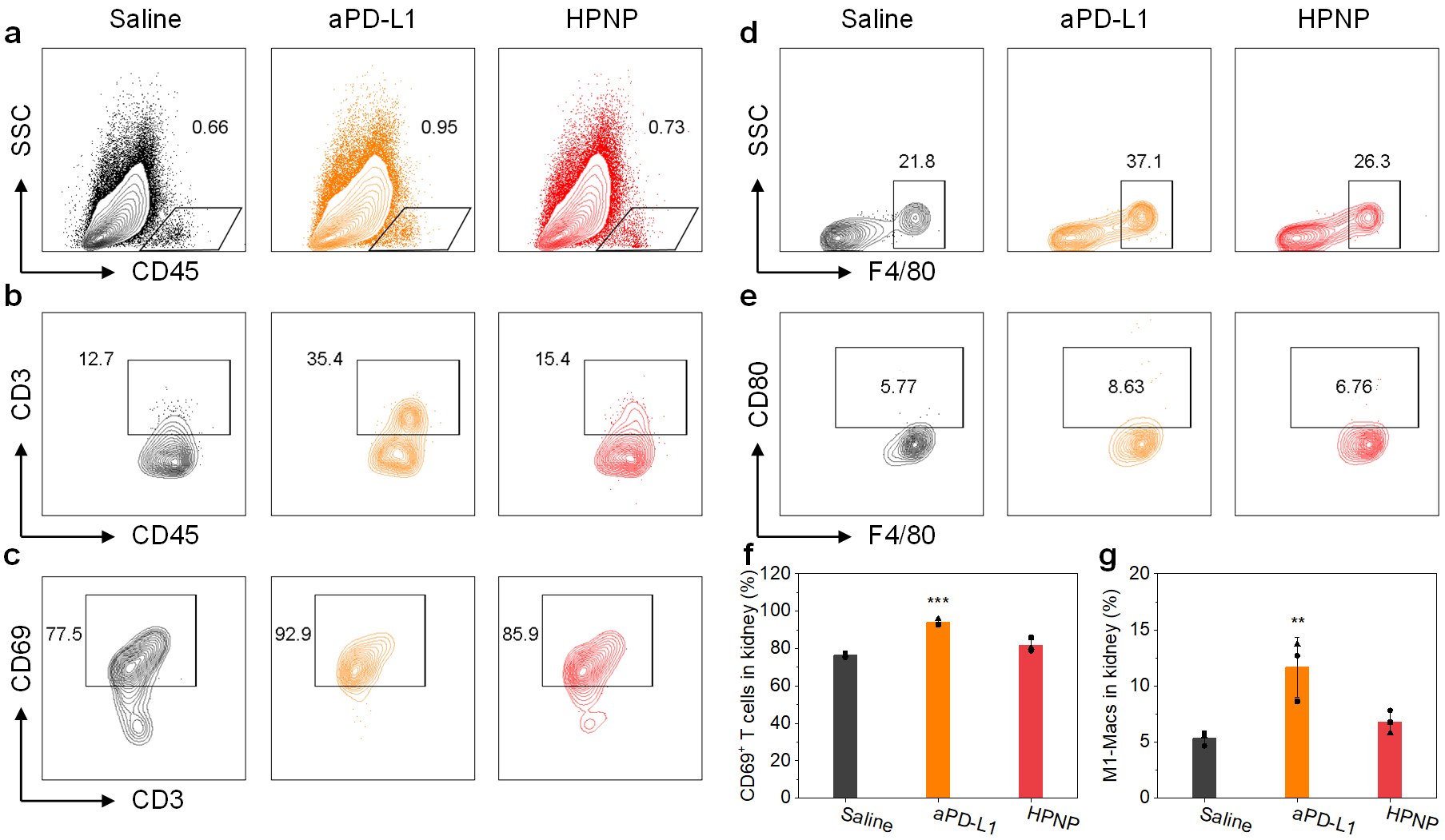
**

**Supplementary Figure 24.** Immune cell infiltrating study in kidney tissues after different treatments. Flow cytometry assay of kidney-infiltrating CD45^+^ leukocytes (**a**), CD45^+^CD3^+^ T cells (**b**), CD3^+^CD69^+^ activated T cells (**c**), F4/80^+^ macrophages (**d**), and F4/80^+^CD80^+^ M1-Macs (**e**) in 4T1 tumor-bearing mice after different treatments (*n*=3). Quantification of CD3^+^CD69^+^ activated T cells (**f**) and F4/80^+^CD80^+^ M1-Macs (**g**) in kidney tissues from 4T1 tumor-bearing mice after different treatments (*n*=3). aPD-L1 versus Saline in **f**: *p* = 0.0003; aPD-L1 versus Saline in **g**: *p* = 0.0088. Statistical significance was calculated via one-way ANOVA with a Tukey post-hoc test. ***p* < 0.01; ****p* < 0.001. The mean values and SD are presented.

**
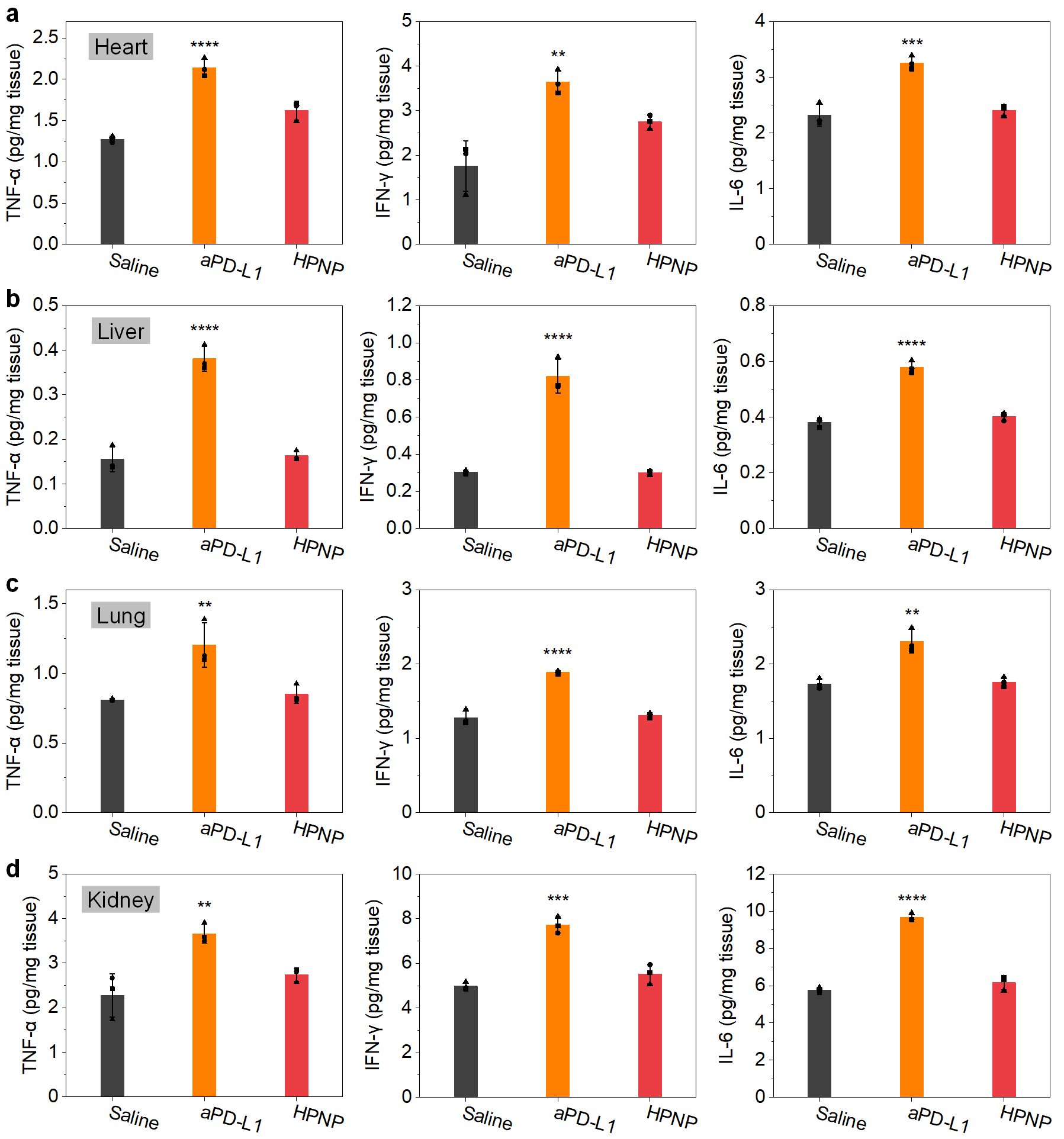
**

**Supplementary Figure 25.** Three major inflammatory cytokines including TNF-α, IFN-γ, and IL-6 in heart (**a**), liver (**b**), lung (**c**), and kidney (**d**) from 4T1 tumor-bearing mice after different treatments (*n*=3). aPD-L1 versus Saline in **a**: *p* < 0.0001 (TNF-α), *p* = 0.0020 (IFN-γ), *p* = 0.0005 (IL-6); aPD-L1 versus Saline in **b**: *p* < 0.0001 (TNF-α), *p* < 0.0001 (IFN-γ), *p* < 0.0001 (IL-6); aPD-L1 versus Saline in **c**: *p* = 0.0070 (TNF-α), *p* < 0.0001 (IFN-γ), *p* = 0.0014 (IL-6); aPD-L1 versus Saline in **d**: *p* = 0.0042 (TNF-α), *p* = 0.0002 (IFN-γ), *p* < 0.0001 (IL-6). Statistical significance was calculated via one-way ANOVA with a Tukey post-hoc test. ***p* < 0.01; ****p* < 0.001; *****p* < 0.0001. The mean values and SD are presented.

**
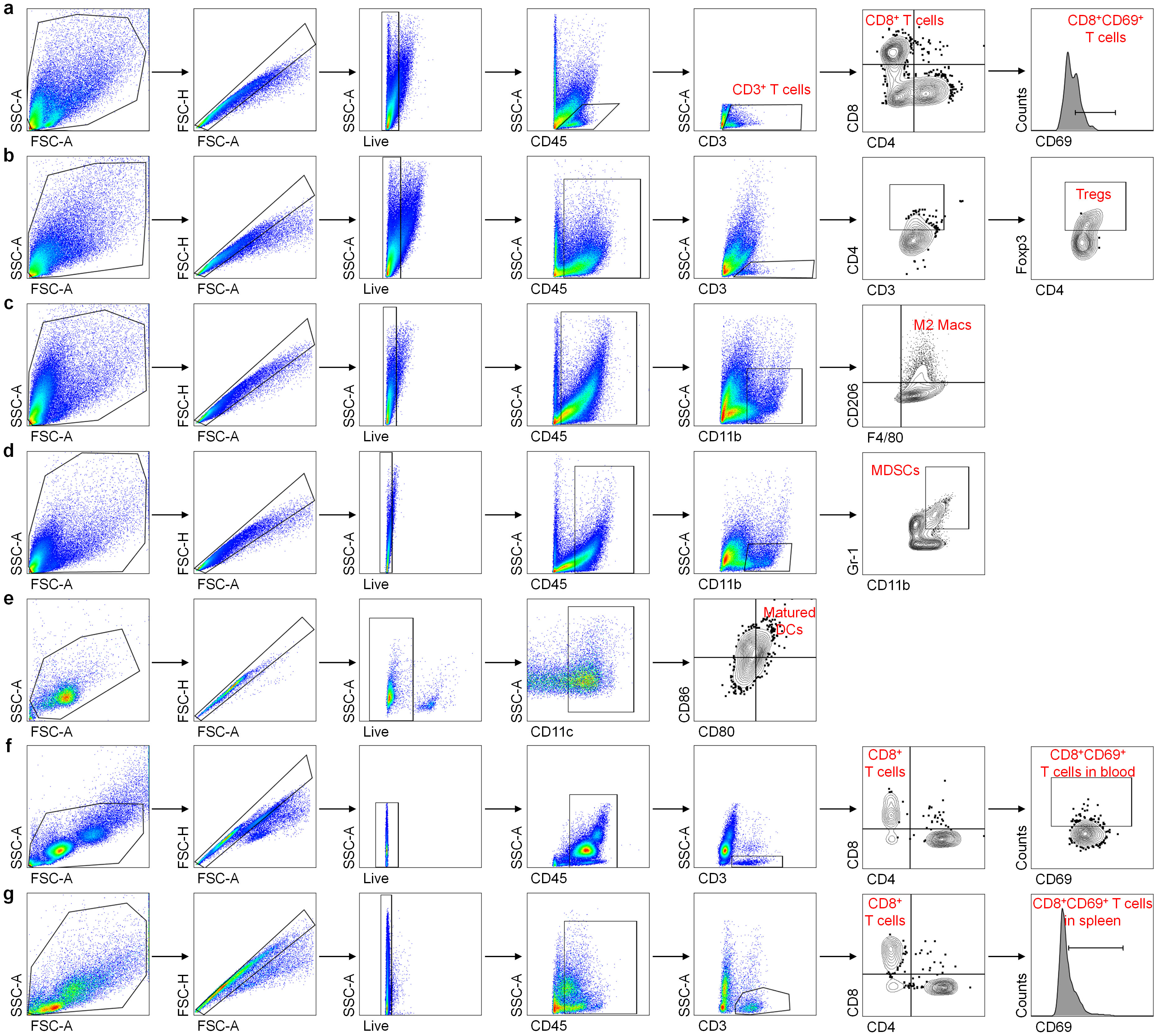
**

**Supplementary Figure 26.** Gating strategies used for flow cytometry analysis of immune cells. Gating strategies to analyze CD3^+^CD8^+^CD69^+^ Teffs (**a**) in Figure 5a-5c, CD3^+^CD4^+^Foxp3^+^ Tregs (**b**) in Figure 5j-5k, CD11b^+^F4/80^+^CD206^+^ M2 Macs (**c**) in Figure 5n-5o, and CD11b^+^Gr-1^+^ MDSCs (**d**) in Figure 5p-5q from the tumors in 4T1 tumor-bearing mice. **e** Gating strategy to analyze matured DCs (CD80^+^CD86^+^) from the tumor-draining lymph nodes in 4T1 tumor-bearing mice in Figure 5i. Gating strategies to analyze CD3^+^CD8^+^CD69^+^ Teffs in the blood (**f**) and spleen (**g**) in 4T1 tumor-bearing mice in Figure 5d.

**
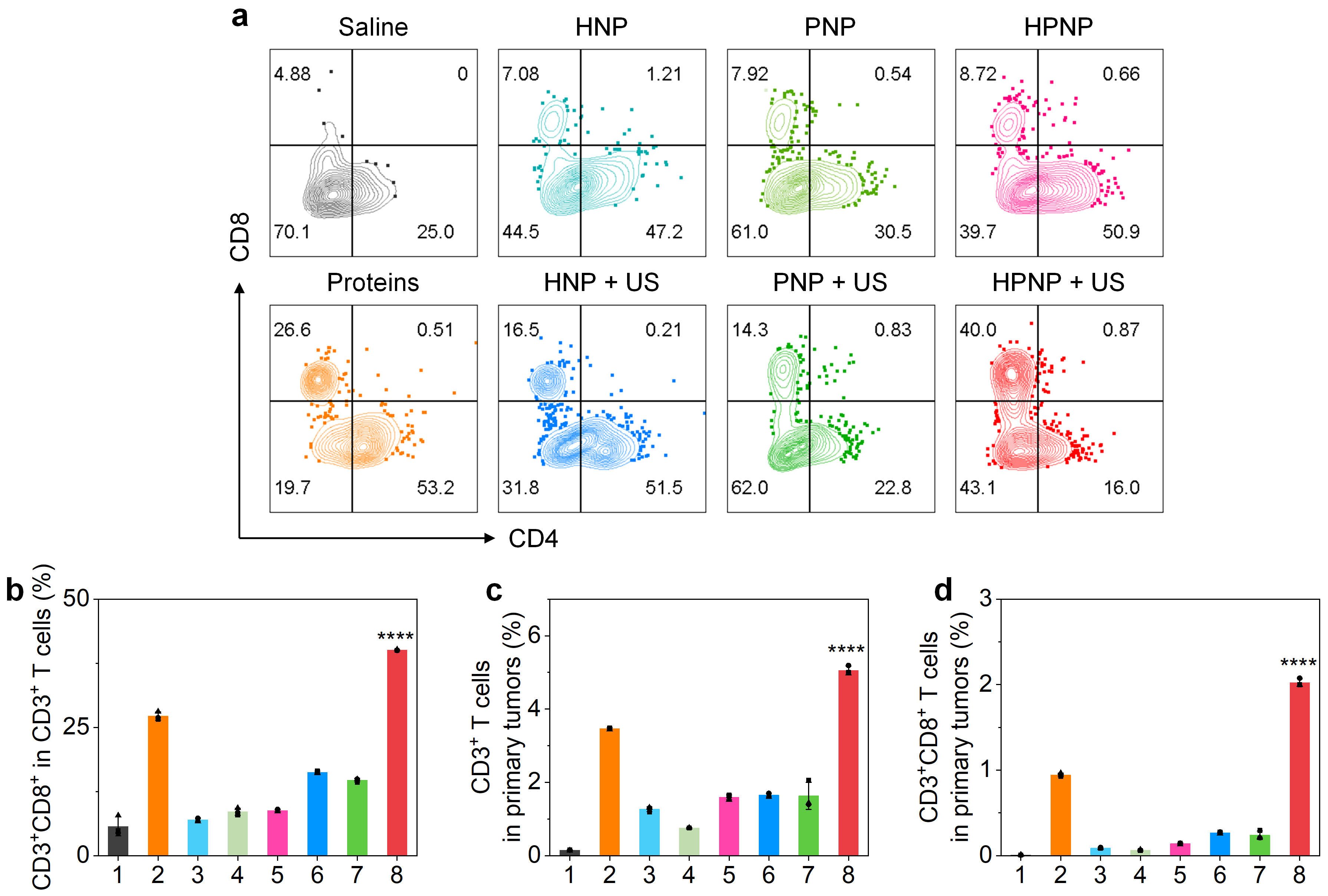
**

**Supplementary Figure 27.** **a** Flow cytometry assay of CD4^+^ and CD8^+^ TILs in primary tumors from 4T1 tumor-bearing mice after different treatments. Quantification of CD3^+^CD8^+^ Teffs in CD3^+^ T cells (**b**), CD3^+^ T cells (**c**) and CD3^+^CD8^+^ Teffs (**d**) in primary tumors from 4T1 tumor-bearing mice after different treatments (*n*=3). 8 versus other groups in **b**, **c**, and **d**: *p* < 0.0001. Statistical significance was calculated via one-way ANOVA with a Tukey post-hoc test. *****p* < 0.0001. The mean values and SD are presented.

**
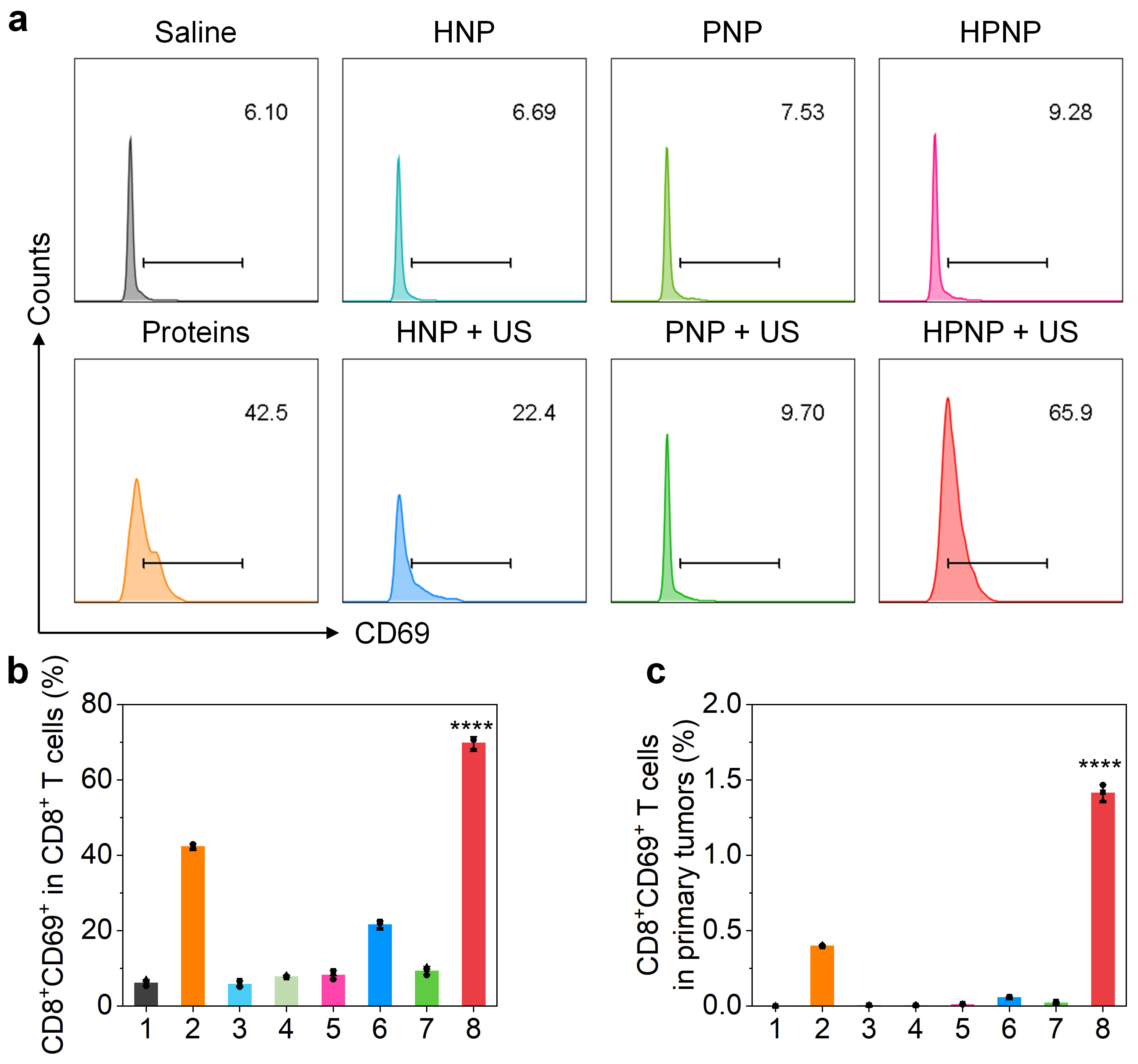
**

**Supplementary Figure 28.** **a** Flow cytometry assay of CD69^+^ Teffs in primary tumors from 4T1 tumor-bearing mice after different treatments. Quantification of CD8^+^CD69^+^ Teffs in CD8^+^ Teffs (**b**) and in primary tumors (**c**) from 4T1 tumor-bearing mice after different treatments (*n*=3). 8 versus 1 in **b** and **c**: *p* < 0.0001. Statistical significance was calculated via one-way ANOVA with a Tukey post-hoc test. *****p* < 0.0001. The mean values and SD are presented.

**
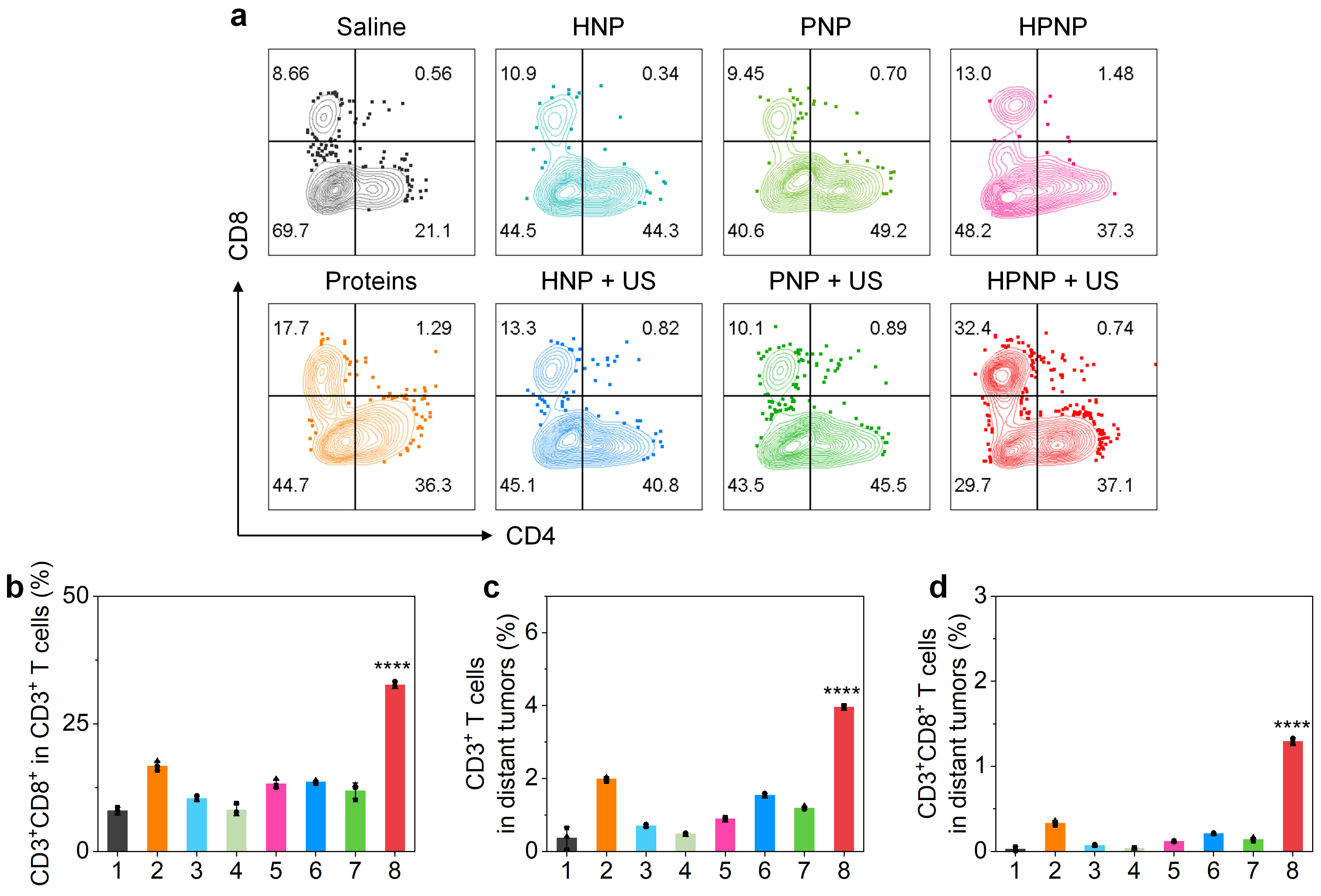
**

**Supplementary Figure 29.** **a** Flow cytometry assay of CD4^+^ and CD8^+^ TILs in distant tumors from 4T1 tumor-bearing mice after different treatments. Quantification of CD3^+^CD8^+^ Teffs in CD3^+^ T cells (**b**), CD3^+^ T cells (**c**) and CD3^+^CD8^+^ Teffs (**d**) in distant tumors from 4T1 tumor-bearing mice after different treatments (*n*=3). 8 versus other groups in **b**, **c**, and **d**: *p* < 0.0001. Statistical significance was calculated via one-way ANOVA with a Tukey post-hoc test. *****p* < 0.0001. The mean values and SD are presented.

**
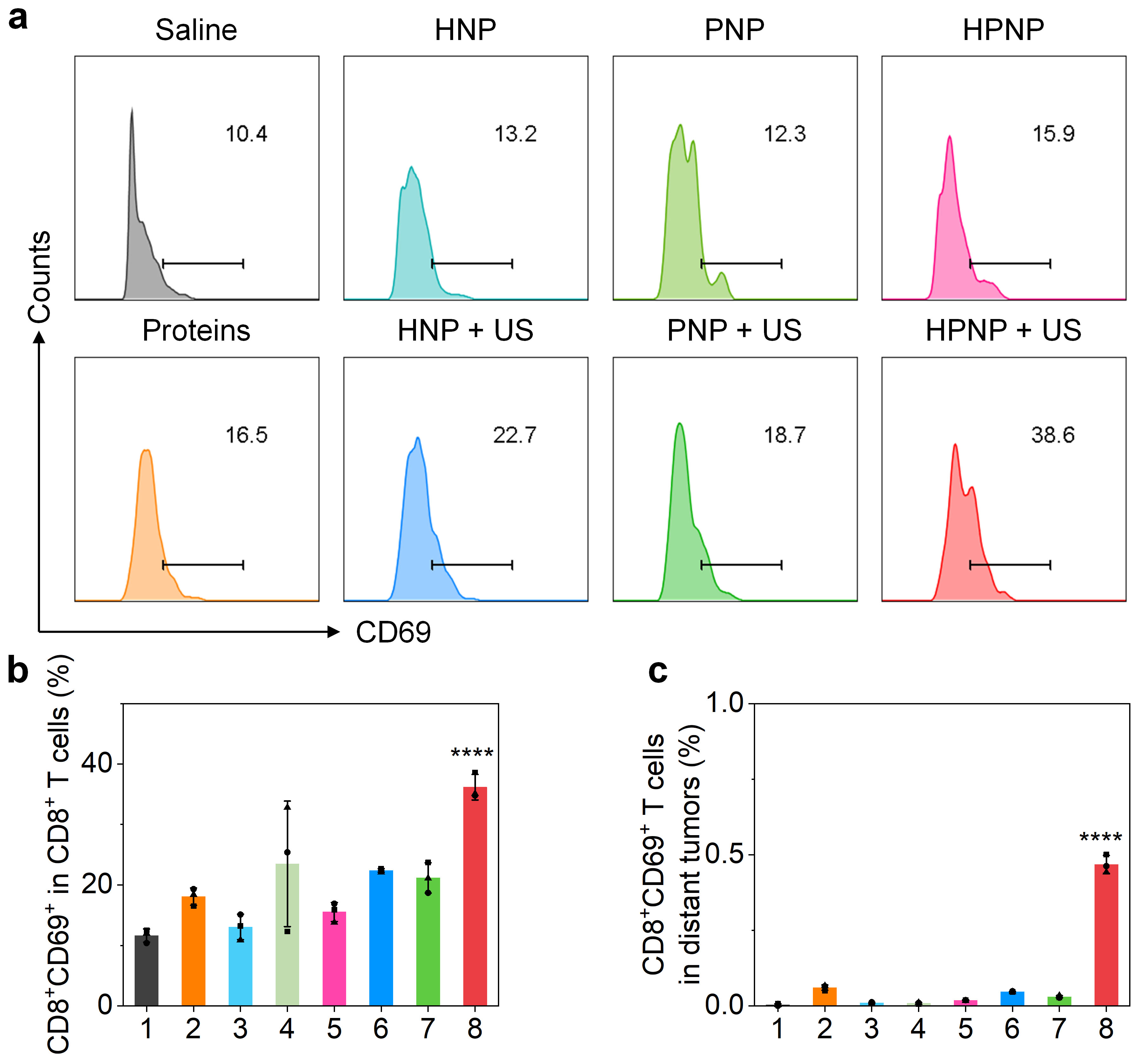
**

**Supplementary Figure 30.** **a** Flow cytometry assay of CD69^+^ Teffs in distant tumors from 4T1 tumor-bearing mice after different treatments. Quantification of CD8^+^CD69^+^ Teffs in CD8^+^ Teffs (**b**) and in distant tumors (**c**) from 4T1 tumor-bearing mice after different treatments (*n*=3). 8 versus 1 in **b** and **c**: *p* < 0.0001. Statistical significance was calculated via one-way ANOVA with a Tukey post-hoc test. *****p* < 0.0001. The mean values and SD are presented.

**
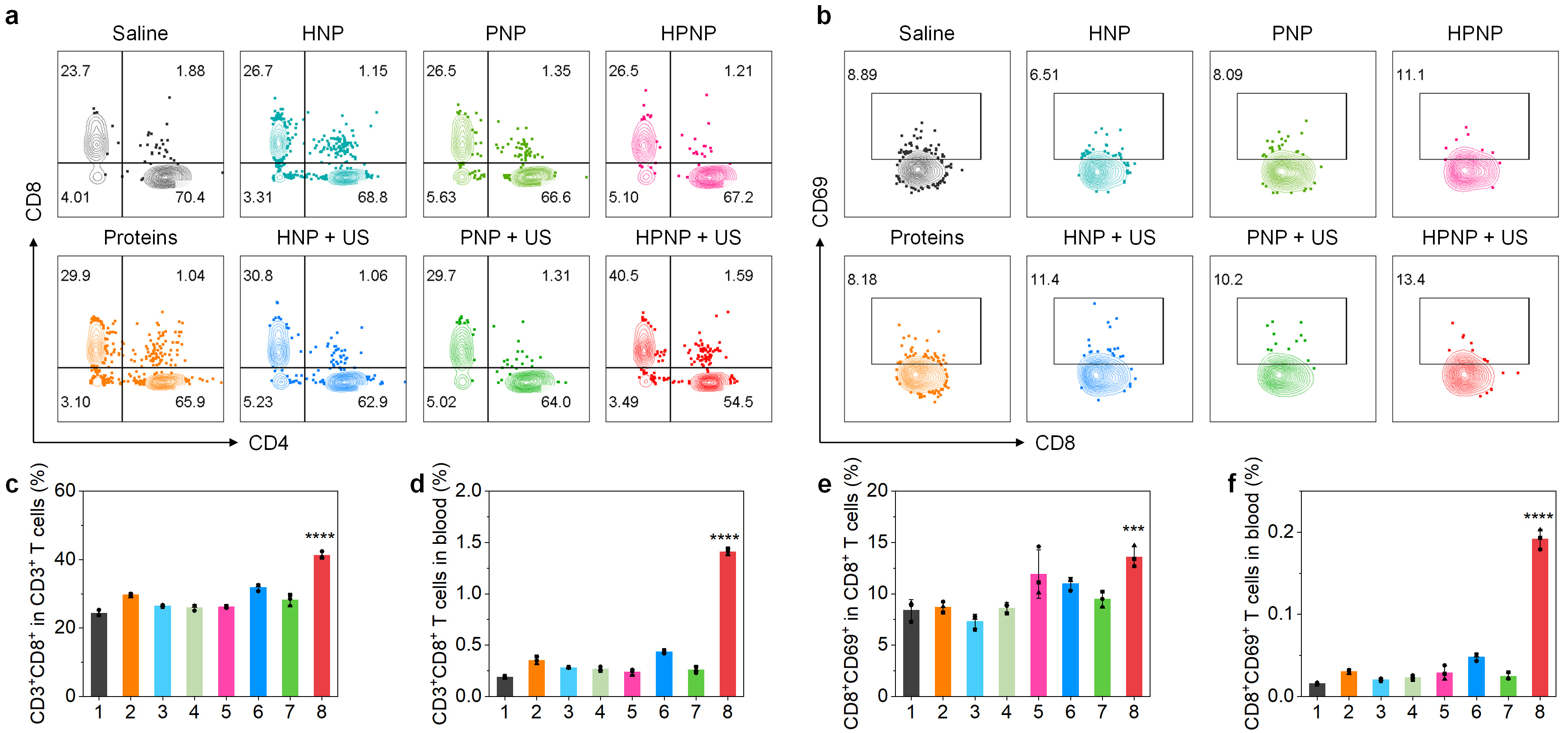
**

**Supplementary Figure 31.** Flow cytometry assay of CD4^+^/CD8^+^ T cells (**a**) and CD8^+^CD69^+^ Teffs (**b**) in blood from 4T1 tumor-bearing mice after different treatments. Quantification of CD3^+^CD8^+^ Teffs in CD3^+^ T cells (**c**), CD3^+^CD8^+^ T cells in blood (**d**), CD8^+^CD69^+^ Teffs in CD8^+^ Teffs (**e**), and CD8^+^CD69^+^ Teffs in blood (**f**) from 4T1 tumor-bearing mice after different treatments (*n*=3). 8 versus 1 in **c**, **d**, and **f**: *p* < 0.0001; 8 versus 1 in **e**: *p* = 0.0005. Statistical significance in **c**, **d**, **e**, and **f** was calculated via one-way ANOVA with a Tukey post-hoc test. ****p* < 0.001 and *****p* < 0.0001. The mean values and SD are presented.

**
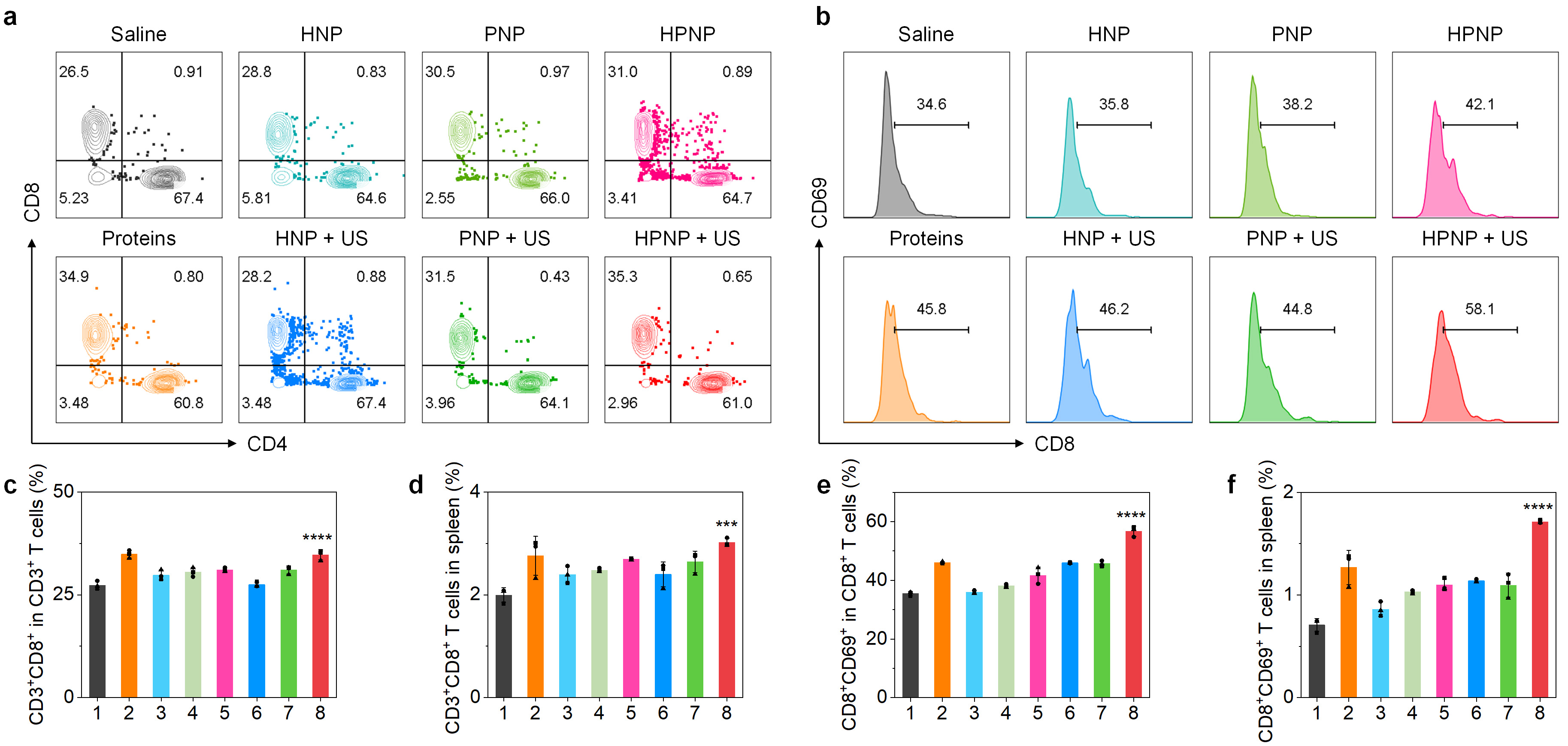
**

**Supplementary Figure 32.** Flow cytometry assay of CD4^+^/CD8^+^ T cells (**a**) and CD8^+^CD69^+^ Teffs (**b**) in spleen from 4T1 tumor-bearing mice after different treatments. Quantification of CD3^+^CD8^+^ Teffs in CD3^+^ T cells (**c**), CD3^+^CD8^+^ T cells in spleen (**d**), CD8^+^CD69^+^ Teffs in CD8^+^ Teffs (**e**), and CD8^+^CD69^+^ Teffs in spleen (**f**) from 4T1 tumor-bearing mice after different treatments (*n*=3). 8 versus 1 in **c**, **e**, and **f**: *p* < 0.0001; 8 versus 1 in **d**: *p* = 0.0002. Statistical significance in **c**, **d**, **e**, and **f** was calculated via one-way ANOVA with a Tukey post-hoc test. ****p* < 0.001 and *****p* < 0.0001. The mean values and SD are presented.

**
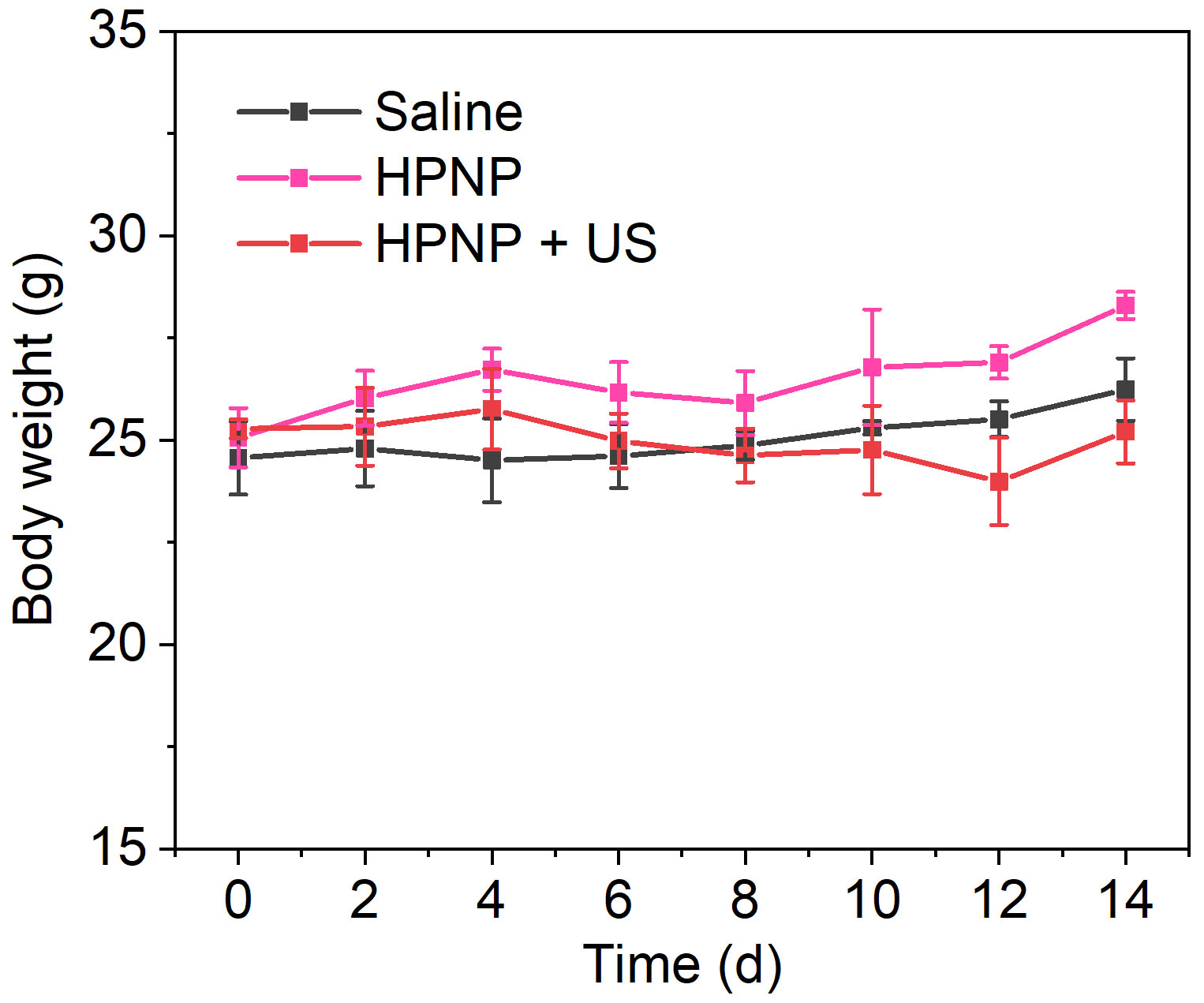
**

**Supplementary Figure 33.** Body weights of 4T1 tumor-bearing immunodeficient NSG mice after different treatments (*n*=5, refer to Figure 5). The mean values and SD are presented.

**
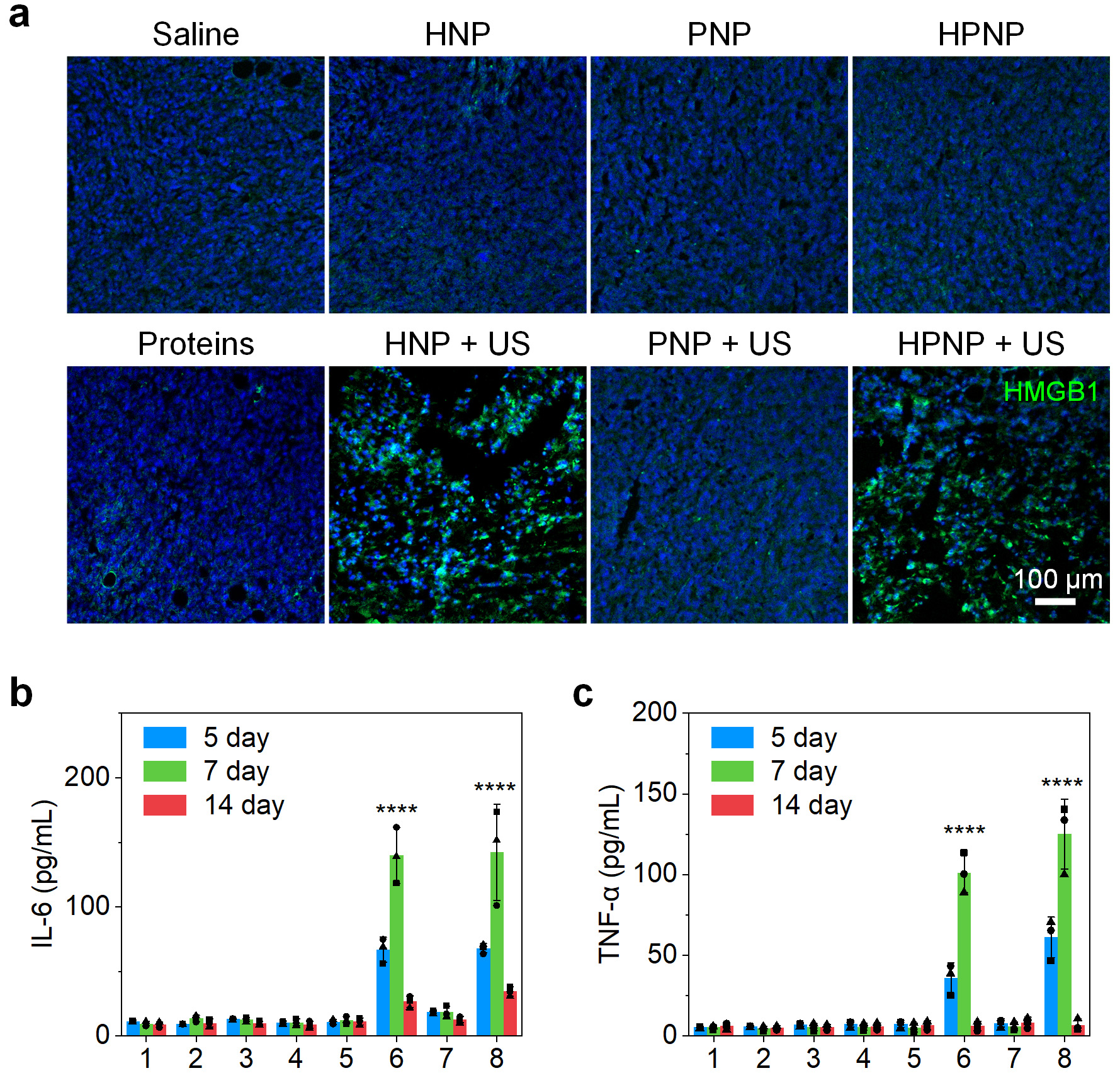
**

**Supplementary Figure 34.** **a** Immunofluorescence staining images of HMGB1 in primary tumor tissues of 4T1 tumor-bearing mice after different treatments. The cell nucleus stained with DAPI and HMGB1 stained with antibodies showed blue and green fluorescence signals, respectively. Images are representative of three biologically independent mice. In vivo cytokine detection of IL-6 (**b**) and TNF-α (**c**) in sera from mice after different treatments at different timepoints (5, 7, and 14 days) (*n*=3). 6 versus 3 at 7 days in **b** and **c**: *p* < 0.0001; 8 versus 5 at 7 days in **b** and **c**: *p* < 0.0001. Statistical significance was calculated via one-way ANOVA with a Tukey post-hoc test. *****p* < 0.0001. The mean values and SD are presented.

**
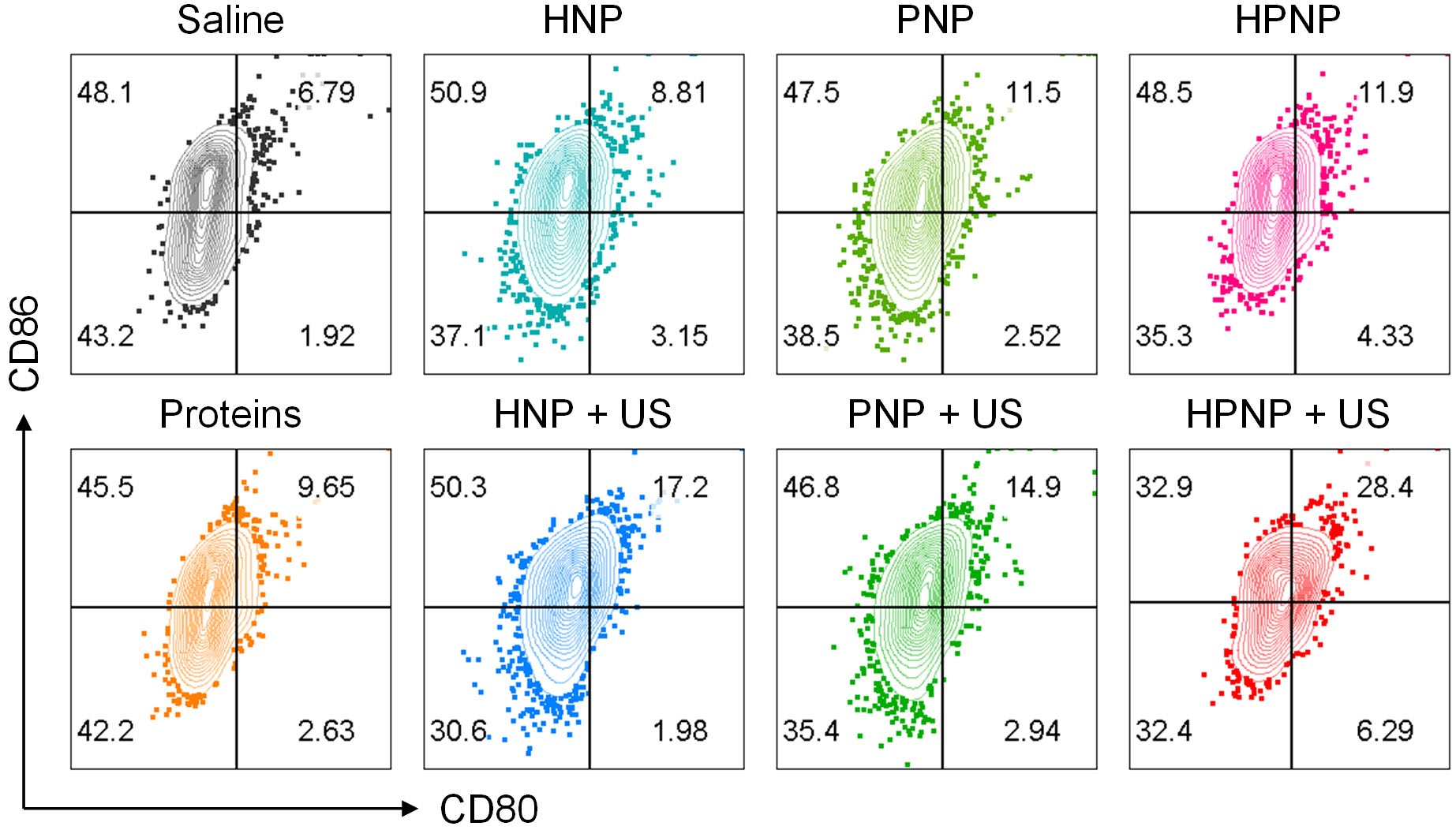
**

**Supplementary Figure 35.** Flow cytometry assay of matured DCs in TDLNs from 4T1 tumor-bearing mice after different treatments.

**
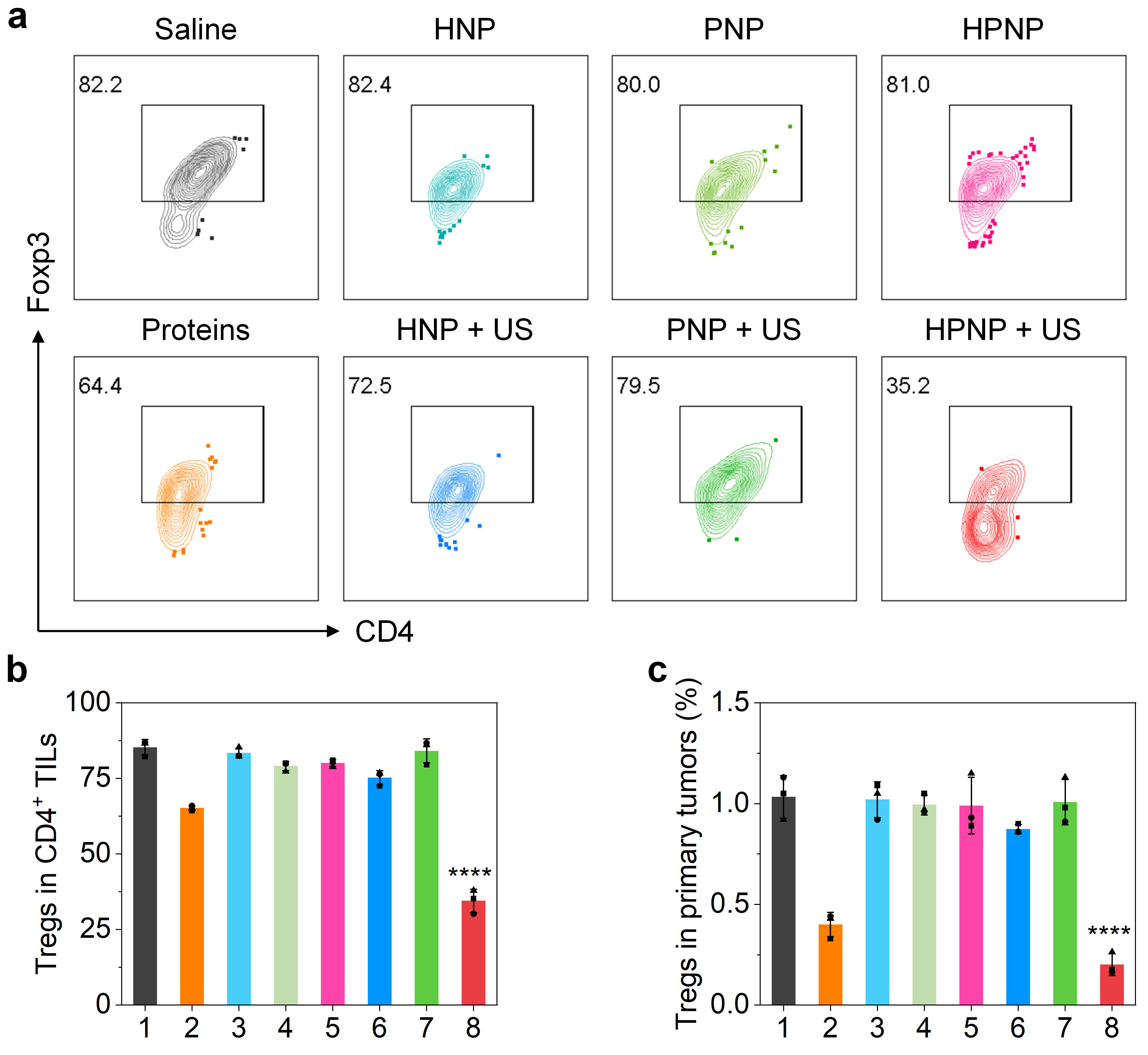
**

**Supplementary Figure 36.** **a** Flow cytometry assay of CD4^+^Foxp3^+^ Tregs in primary tumors from 4T1 tumor-bearing mice after different treatments. Quantification of CD4^+^Foxp3^+^ Tregs in CD4^+^ T cells (**b**) and in primary tumors (**c**) from 4T1 tumor-bearing mice after different treatments (*n*=3). 8 versus 1 in **b** and **c**: *p* < 0.0001. Statistical significance was calculated via one-way ANOVA with a Tukey post-hoc test. *****p* < 0.0001. The mean values and SD are presented.

**
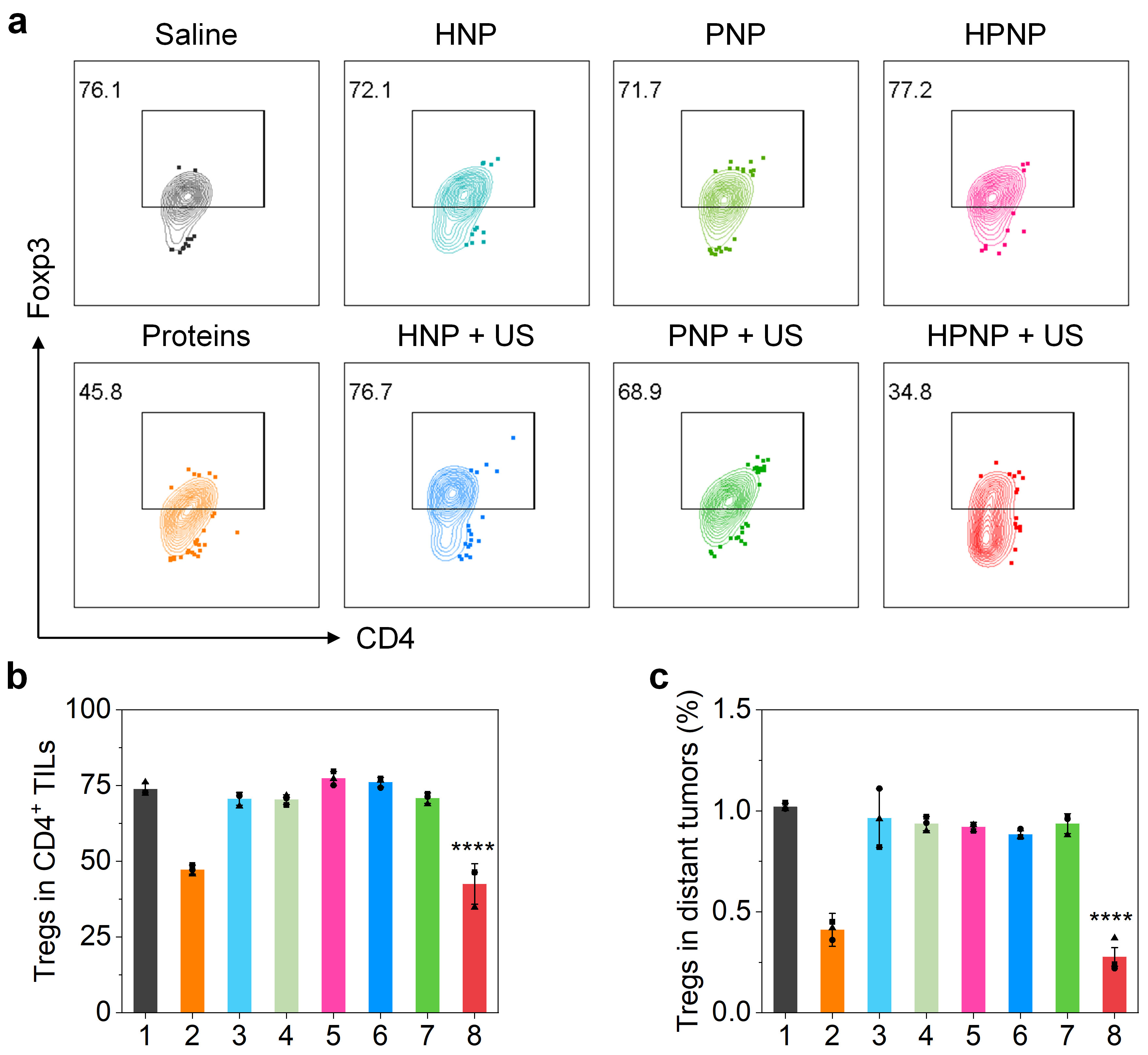
**

**Supplementary Figure 37.** **a** Flow cytometry assay of CD4^+^Foxp3^+^ Tregs in distant tumors from 4T1 tumor-bearing mice after different treatments. Quantification of CD4^+^Foxp3^+^ Tregs in CD4^+^ T cells (**b**) and in distant tumors (**c**) from 4T1 tumor-bearing mice after different treatments (*n*=3). 8 versus 1 in **b** and **c**: *p* < 0.0001. Statistical significance was calculated via one-way ANOVA with a Tukey post-hoc test. *****p* < 0.0001. The mean values and SD are presented.

**
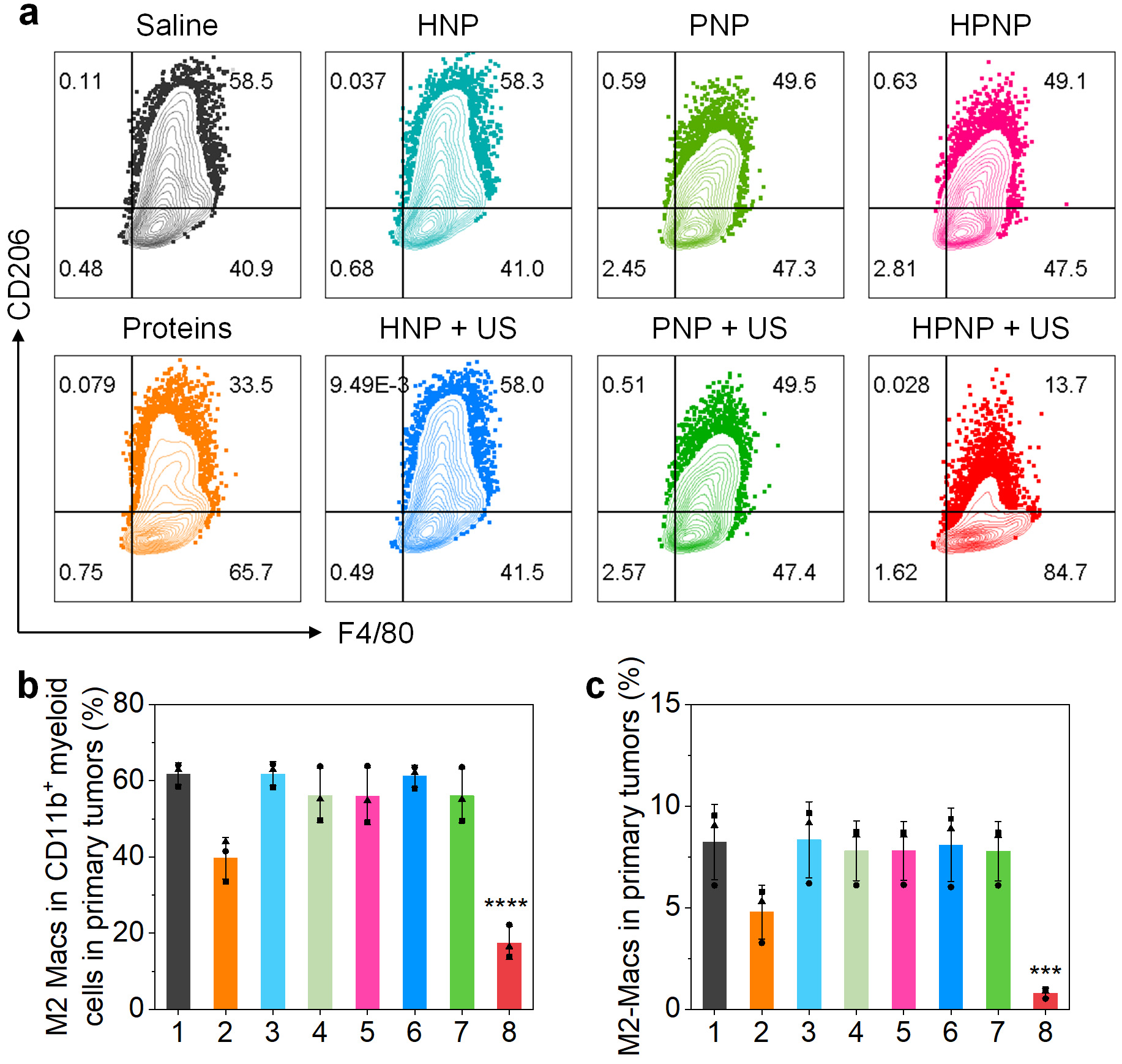
**

**Supplementary Figure 38.** **a** Flow cytometry assay of CD11b^+^F4/80^+^CD206^+^ M2 Macs in primary tumors from 4T1 tumor-bearing mice after different treatments. Quantification of M2 Macs in CD11b^+^ myeloid cells (**b**) and in primary tumors (**c**) from 4T1 tumor-bearing mice after different treatments (*n*=3). 8 versus 1 in **b**: *p* < 0.0001; 8 versus 1 in **c**: *p* = 0.0004. Statistical significance was calculated via one-way ANOVA with a Tukey post-hoc test. ****p* < 0.001 and *****p* < 0.0001. The mean values and SD are presented.

**
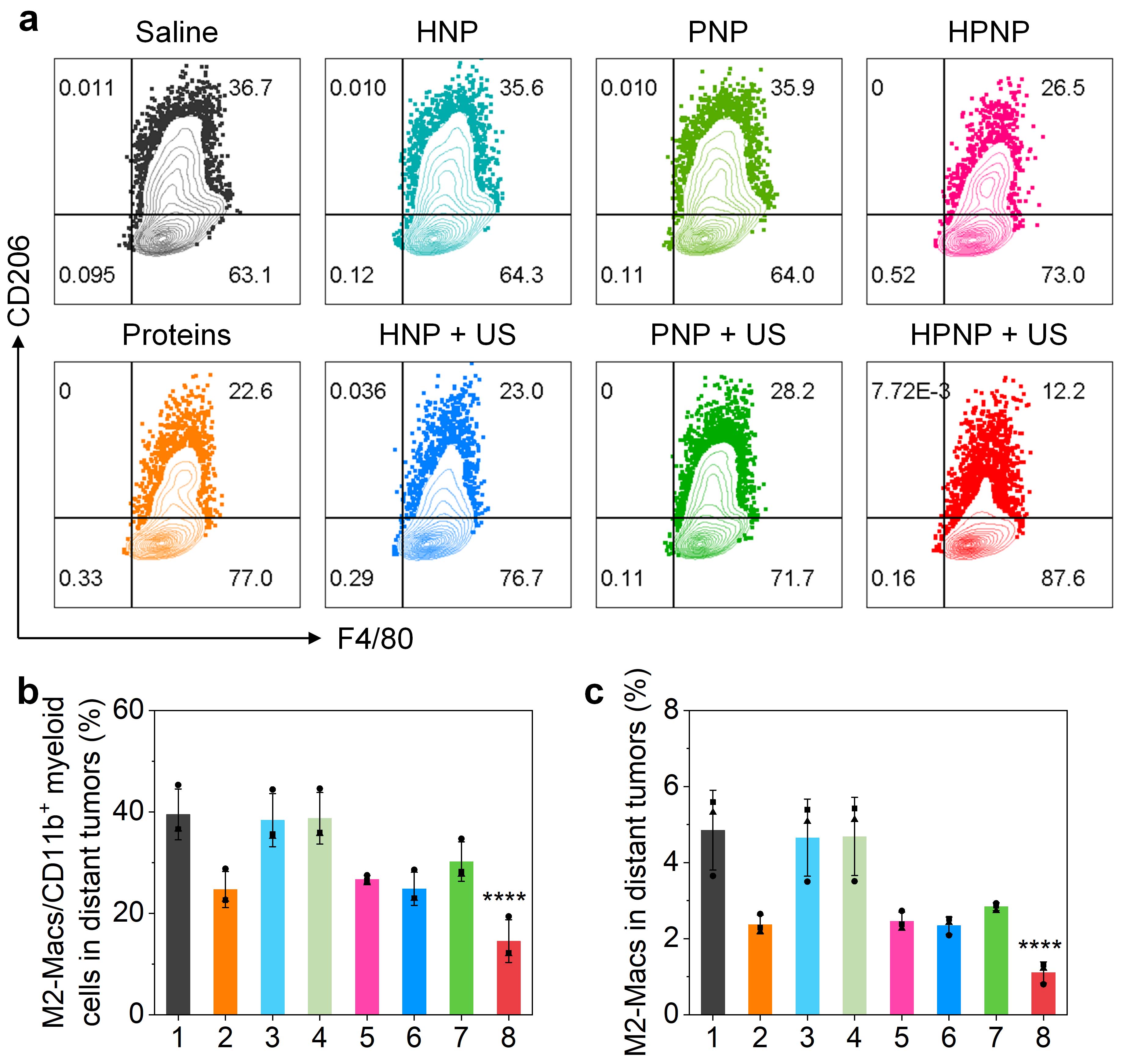
**

**Supplementary Figure 39.** **a** Flow cytometry assay of CD11b^+^F4/80^+^CD206^+^ M2 Macs in distant tumors from 4T1 tumor-bearing mice after different treatments. Quantification of M2 Macs in CD11b^+^ myeloid cells (**b**) and in distant tumors (**c**) from 4T1 tumor-bearing mice after different treatments (*n*=3). 8 versus 1 in **b** and **c**: *p* < 0.0001. Statistical significance was calculated via one-way ANOVA with a Tukey post-hoc test. *****p* < 0.0001. The mean values and SD are presented.

**
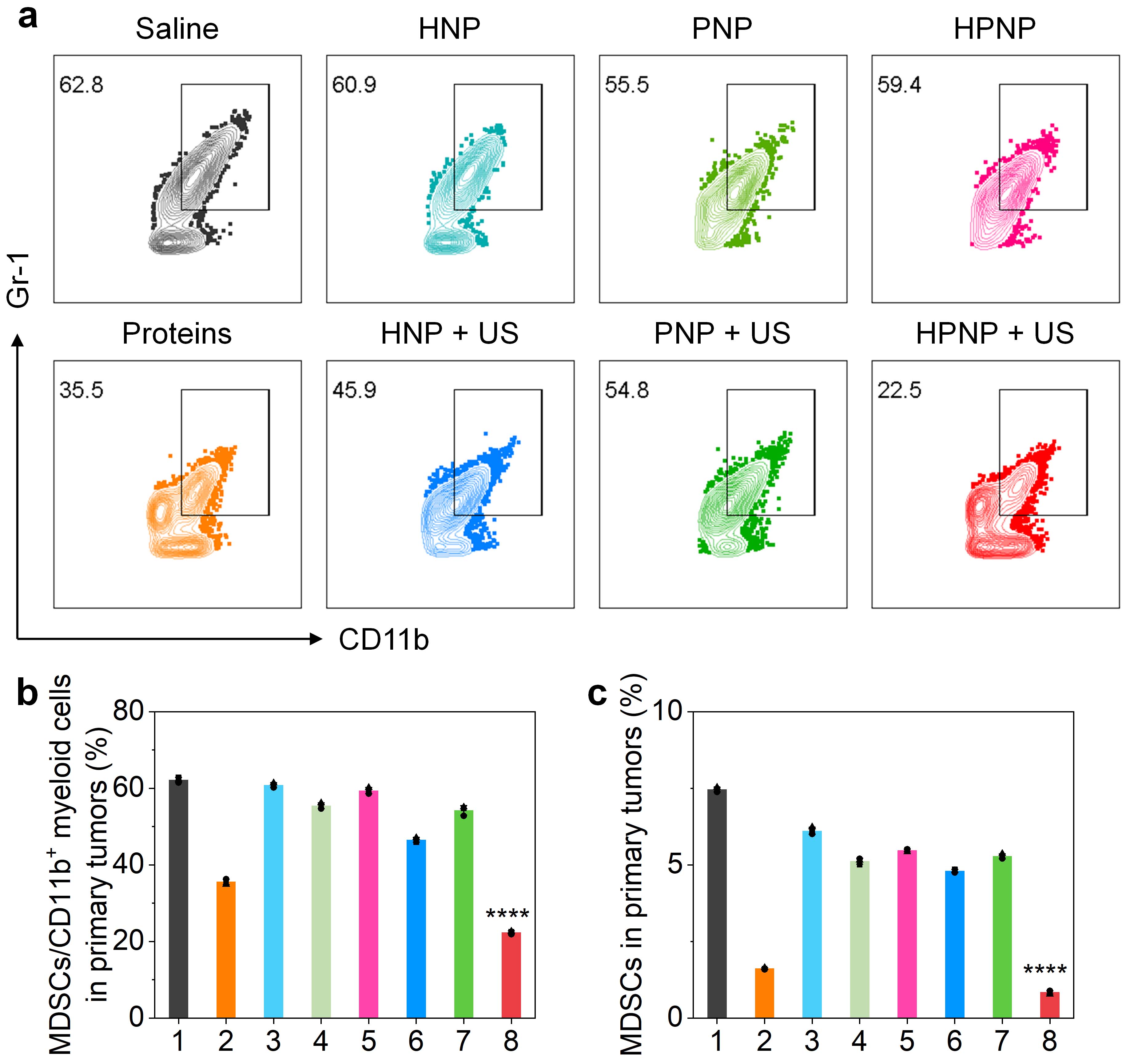
**

**Supplementary Figure 40.** **a** Flow cytometry assay of CD11b^+^Gr-1^+^ MDSCs in primary tumors from 4T1 tumor-bearing mice after different treatments. Quantification of MDSCs in CD11b^+^ myeloid cells (**b**) and in primary tumors (**c**) from 4T1 tumor-bearing mice after different treatments (*n*=3). 8 versus 1 in **b** and **c**: *p* < 0.0001. Statistical significance was calculated via one-way ANOVA with a Tukey post-hoc test. *****p* < 0.0001. The mean values and SD are presented.

**
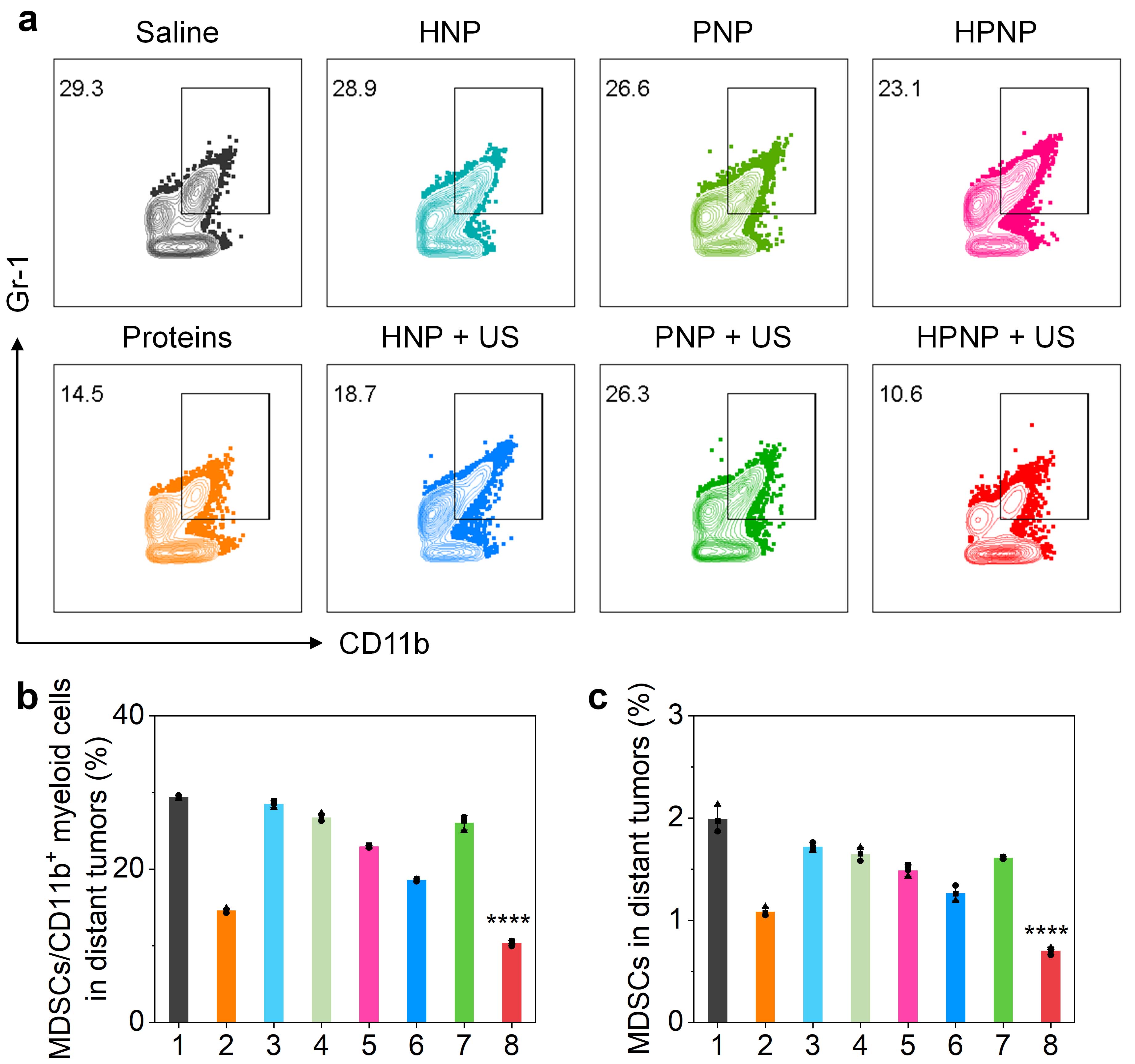
**

**Supplementary Figure 41.** **a** Flow cytometry assay of CD11b^+^Gr-1^+^ MDSCs in distant tumors from 4T1 tumor-bearing mice after different treatments. Quantification of MDSCs in CD11b^+^ myeloid cells (**b**) and in distant tumors (**c**) from 4T1 tumor-bearing mice after different treatments (*n*=3). 8 versus 1 in **b** and **c**: *p* < 0.0001. Statistical significance was calculated via one-way ANOVA with a Tukey post-hoc test. *****p* < 0.0001. The mean values and SD are presented.

**
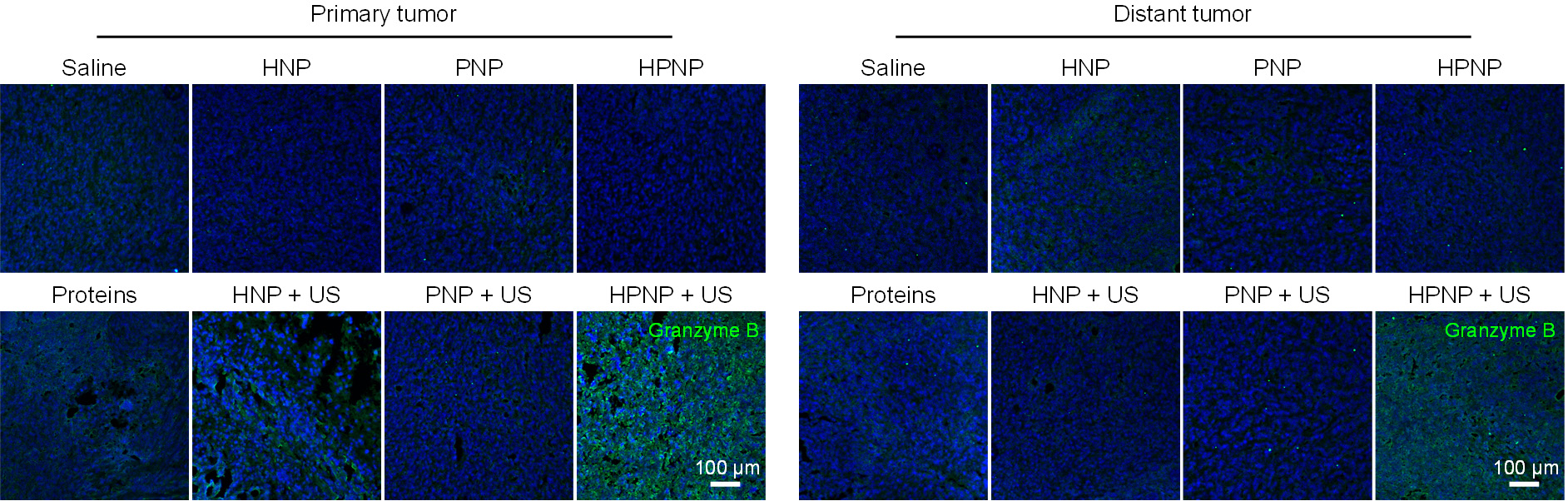
**

**Supplementary Figure 42.** Immunofluorescence staining images of granzyme B in primary and distant tumor tissues of 4T1 tumor-bearing mice after different treatments. The cell nucleus stained with DAPI and granzyme B stained with antibodies showed blue and green fluorescence signals, respectively. Images are representative of three biologically independent mice.

**
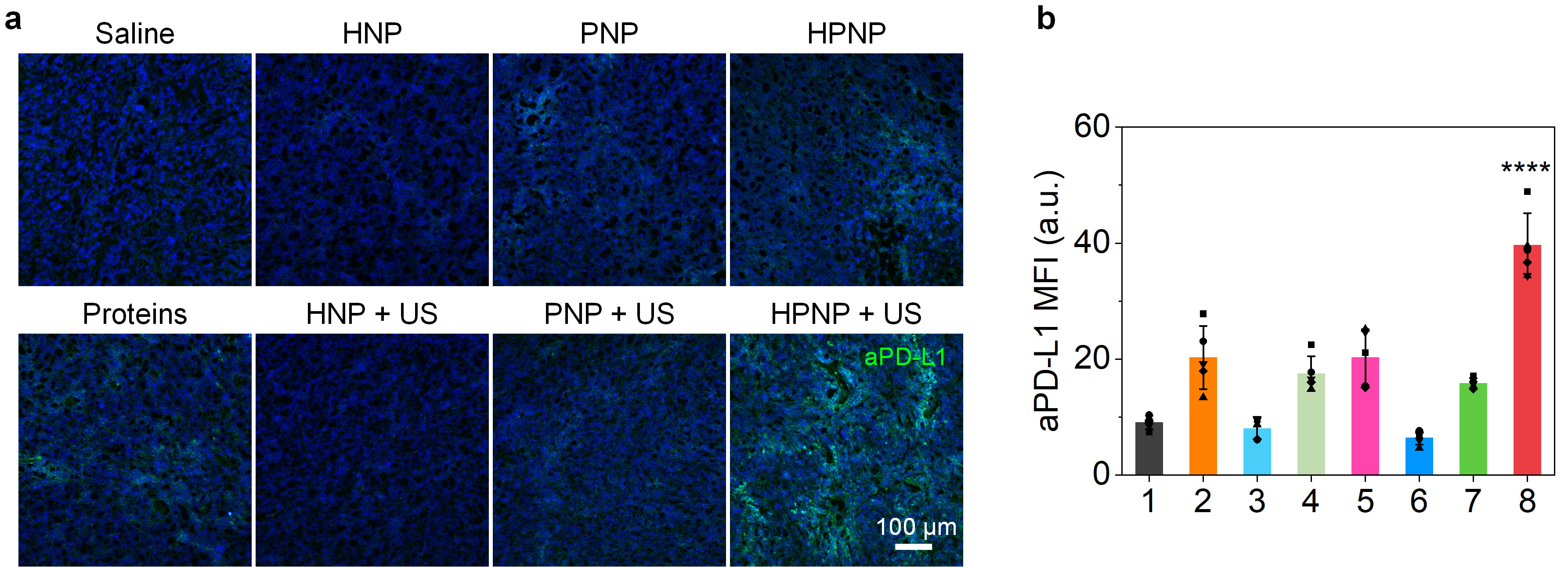
**

**Supplementary Figure 43.** Immunofluorescence staining images (**a**) and quantification (**b**) of aPD-L1 release in primary tumor tissues of 4T1 tumor-bearing mice after different treatments (*n*=5). 8 versus other groups in **b**: *p* < 0.0001. Statistical significance was calculated via one-way ANOVA with a Tukey post-hoc test. *****p* < 0.0001. The mean values and SD are presented.


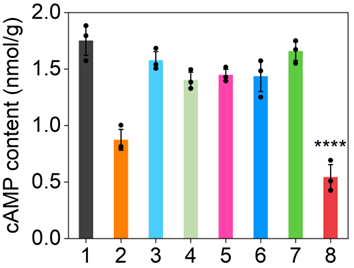


**Supplementary Figure 44.** The Ade content in primary tumors of 4T1 tumor-bearing mice after different treatments (*n*=3). 8 versus saline: *p* < 0.0001. 1, saline; 2, proteins; 3, HNP; 4, PNP; 5, HPNP; 6, HNP + US; 7, PNP + US; 8, HPNP + US (injection dose: 200 μL, [HP]=1 mmol/L, or [ADA]=40 U/mL; sono-irradiation: 1.0 MHz, 1.2 W/cm^2^, 50% duty cycle for 6 min). Statistical significance was calculated via one-way ANOVA with a Tukey post-hoc test. *****p* < 0.0001. The mean values and SD are presented.

**
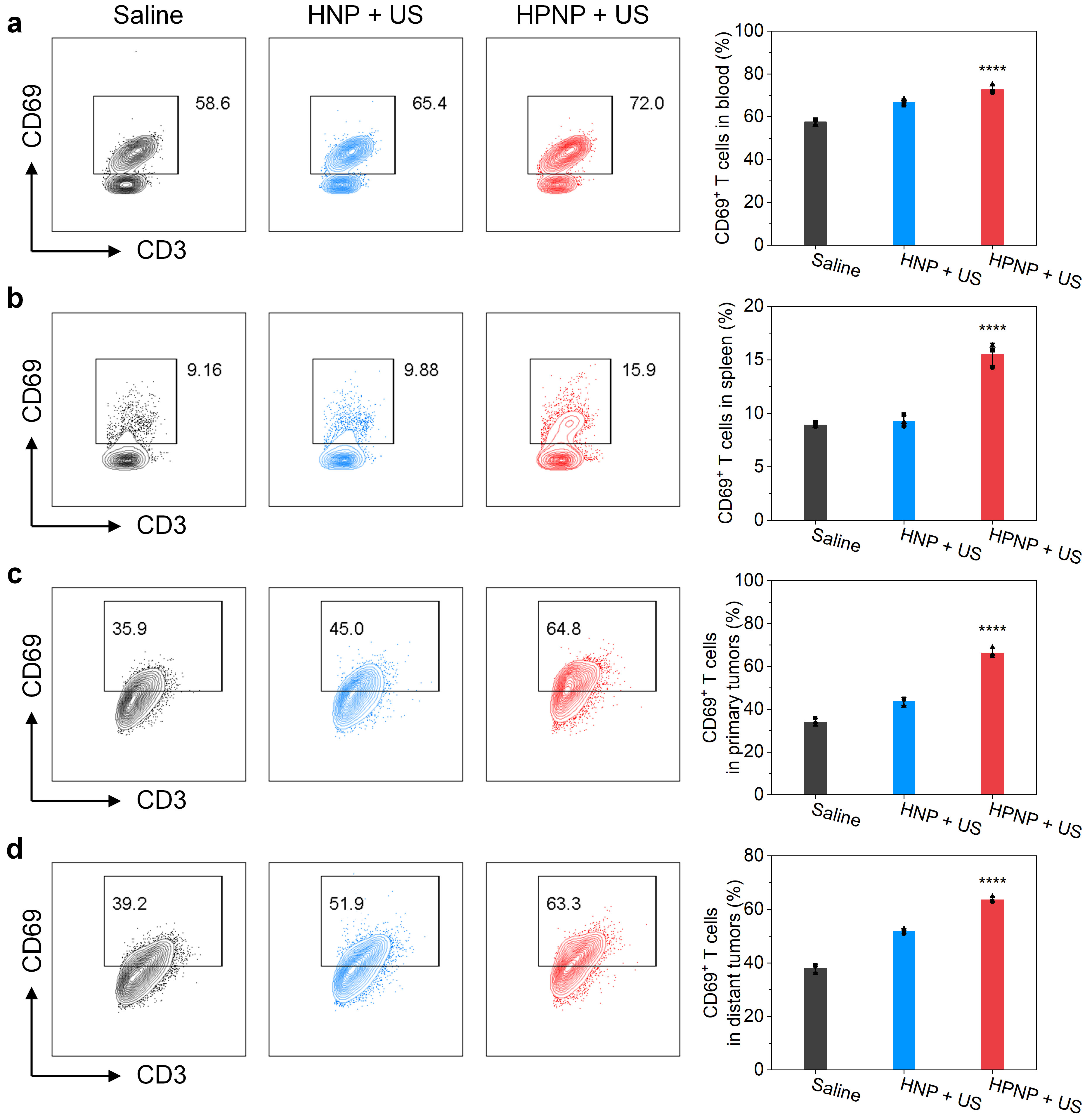
**

**Supplementary Figure 45.** In vivo mechanistic study of nano-immunocomplex-mediated activatable sono-metabolic checkpoint trimodal cancer therapy. Flow cytometry assay and quantification of activated T lymphocytes (CD3^+^CD69^+^) in blood (**a**), spleen (**b**), primary tumors (**c**), and distant tumors (**d**) from CT26 tumor-bearing mice after different treatments (*n*=3). HPNP + US versus Saline in **a**, **b**, **c**, and **d**: *p* < 0.0001. Statistical significance was calculated via one-way ANOVA with a Tukey post-hoc test. *****p* < 0.0001. The mean values and SD are presented.

**
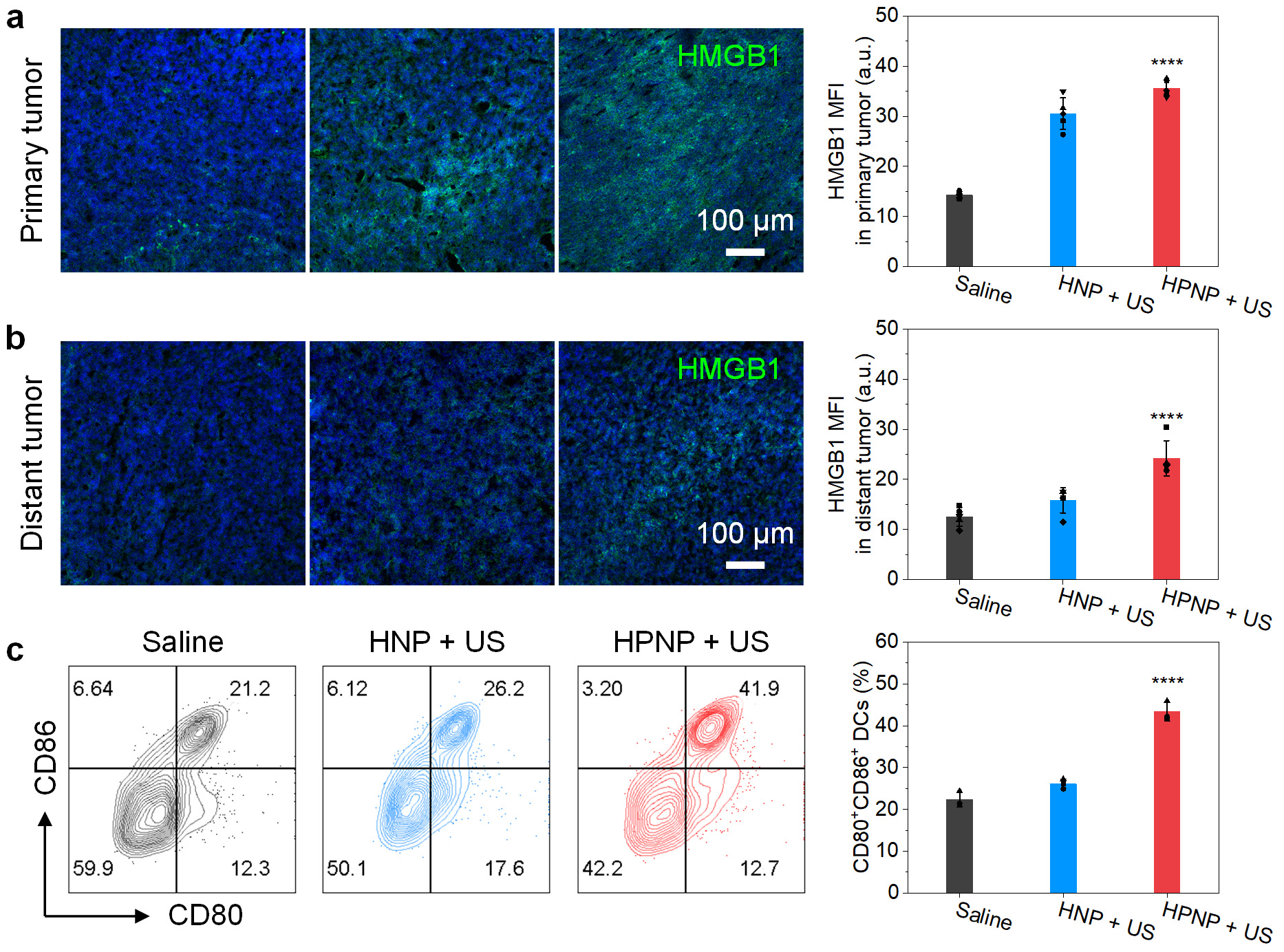
**

**Supplementary Figure 46.** Immunofluorescence staining images and quantification of HMGB1 expression in primary (**a**) and distant (**b**) tumor tissues of CT26 tumor-bearing mice after different treatments (*n*=5). The cell nucleus stained with DAPI and HMGB1 stained with antibodies showed blue and green fluorescence signals, respectively. HPNP + US versus Saline in **a** and **b**: *p* < 0.0001. **c** Flow cytometry assay and quantification of matured DCs (CD80^+^CD86^+^) in TDLNs from CT26 tumor-bearing mice after different treatments (*n*=3). HPNP + US versus Saline in **c**: *p* < 0.0001. Statistical significance was calculated via one-way ANOVA with a Tukey post-hoc test. *****p* < 0.0001. The mean values and SD are presented.

**
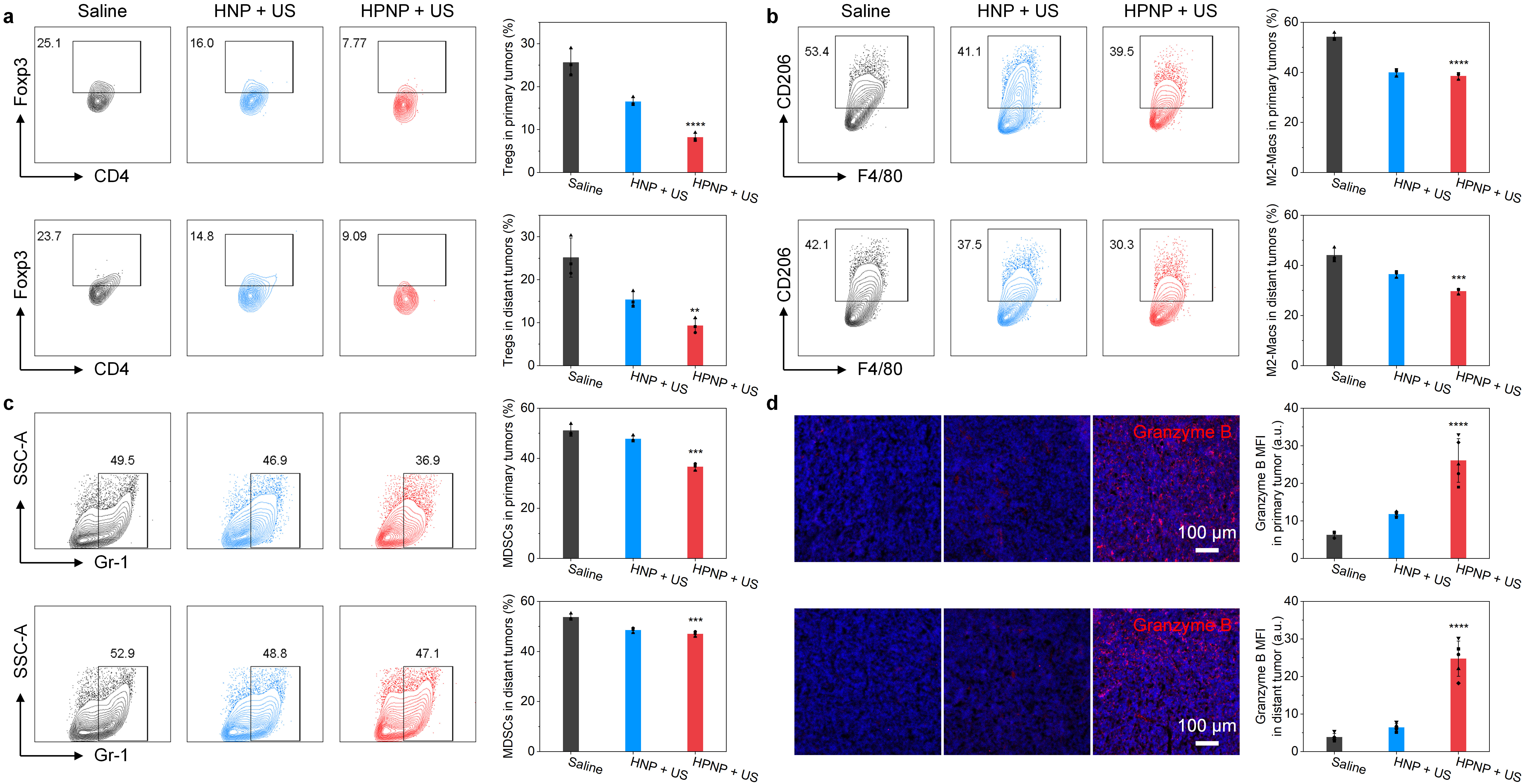
**

**Supplementary Figure 47.** In vivo immunosuppressive TME study of nano-immunocomplex-mediated activatable sono-metabolic checkpoint trimodal cancer therapy. Flow cytometry assay and quantification of CD4^+^Foxp3^+^ Tregs (**a**), F4/80^+^CD206^+^ M2 Macs (**b**), and CD11b^+^Gr-1^+^ MDSCs (**c**) in primary and distant tumors from CT26 tumor-bearing mice after different treatments (*n*=3). HPNP + US versus Saline in **a**: *p* < 0.0001 (primary tumor) and *p* = 0.0016 (distant tumor); HPNP + US versus Saline in **b**: *p* < 0.0001 (primary tumor) and *p* = 0.0002 (distant tumor); HPNP + US versus Saline in **c**: *p* = 0.0001 (primary tumor) and *p* = 0.0007 (distant tumor). **d** Quantification of granzyme B expression in primary and distant tumors from CT26 tumor-bearing mice by immunofluorescence staining after different treatments (*n*=5). HPNP + US versus Saline in **d**: *p* < 0.0001. Statistical significance was calculated via one-way ANOVA with a Tukey post-hoc test. ***p* < 0.01, ****p* < 0.001, *****p* < 0.0001. The mean values and SD are presented.
